# Prenyl Pterocarpans from Algerian *Bituminaria bituminosa* and Their Effects on Neuroblastoma

**DOI:** 10.3390/molecules29153678

**Published:** 2024-08-02

**Authors:** Hakim Benhabrou, Fatma Bitam, Luigia Cristino, Alessandro Nicois, Marianna Carbone, Dibi Ammar, Margherita Gavagnin, Maria Letizia Ciavatta

**Affiliations:** 1 Université de Batna 1, Faculté des Sciences de la Matière, Département de Chimie, Laboratoire de Chimie et Chimie de l’Environnement (LCCE), Batna 05000, Algeria; hakim.benhabrou@univ-batna.dz (H.B.); amdibi@yahoo.fr (D.A.); 2 Université de Batna 2, Faculté de Médecine, Département de Pharmacie, Batna 05000, Algeria; f.bitam@univ-batna2.dz; 3 Consiglio Nazionale delle Ricerche, Istituto di Chimica Biomolecolare, Via Campi Flegrei 34, 80078 Pozzuoli, Italy; anicois@icb.cnr.it (A.N.); mcarbone@icb.cnr.it (M.C.); mgavagnin@icb.cnr.it (M.G.); 4 Università di Urbino ‘Carlo Bo’, Dipartimento di Scienze Biomolecolari, Via Santa Chiara, 27, 61029 Urbino, Italy

**Keywords:** *Bituminaria bituminosa*, natural products chemistry, pterocarpans, biological activity, neuroblastoma

## Abstract

The pterocarpan fraction from aerial parts of *Bituminaria bituminosa* was investigated for both chemical characterization and biological evaluation. Chemical studies were in accordance with the literature data on *Bituminaria* genus resulting in the identification of typical 4,8-prenyl pterocarpans. Three new members, bituminarins A–C (**1**–**3**), were isolated along with main bitucarpin A (**4**), erybraedin C (**5**) and erybraedin D (**6**) already reported from this plant. Further, biological studies evidenced antiproliferative properties of the most abundant pterocarpans **4** and **5** on neuroblastoma SH-SY5Y cell line, in agreement with previously described antiproliferative activity of these compounds against cancer cell lines other than neuroblastoma. The structure and the stereochemistry of the new molecules was determined by extensive spectroscopic analysis and chemical derivatization methods. The biological investigation was carried out by using an assay platform based on a live-cell imaging system revealing an apoptotic cell death induction.

## 1. Introduction

*Bituminaria bituminosa* (L.) Stirt. (syn. *Psoralea bituminosa* L.), commonly known as pitch trefoil, is a scrambling perennial legume species (Fam. Fabaceae) mainly distributed in Mediterranean coastal regions [1]. The plant has pinkish-mauve flowers and trifoliate leaves with a characteristic smell of bitumen [1]. *B. bituminosa* produces a large amount of biomass with significant nutritive value that is mainly utilized for feeding sheep and goats [2] even though some concern on the forage use has been raised due to the presence of photoreactive compounds in the plant secondary metabolite pool [3]. The phytochemicals described in the literature for *B. bituminosa* include different classes of compounds, with pterocarpans and furanocoumarins being the most typical components of the aerial parts of the plant [4,5,6,7]. Pterocarpans and furanocoumarins have also been detected in the volatile fraction from leaves and flowers [8]. Among pterocarpans, bitucarpin A (**4**) and erybraedin C (**5**) are the most abundant compounds detected in the leaves of the plant [5,6,9]. Other main phytochemicals reported from the aerial parts of *B. bituminosa* comprise caffeic and coumaric acids, apigenin glucosyl derivatives, luteolin, daidzein, lignans and soyasaponins [10,11].

Pterocarpans are potent plant defensive metabolites (phytoalexins), mainly produced by species belonging to Fabaceae family, that have been demonstrated to have a variety of interesting pharmacological properties [12]. Naturally occurring pterocarpans are the second largest group of isoflavonoids [13] and are often used in traditional medicine in different countries as an alternative and supplementary therapy. Interestingly, literature data show that pterocarpans from *Bituminaria* species are characterized by the presence of one or two prenyl substituents, which are typically located at C-4 and C-8 on the benzofuran-benzopyran tetracyclic ring system of the pterocarpan nucleus [14].

**Figure 1:**
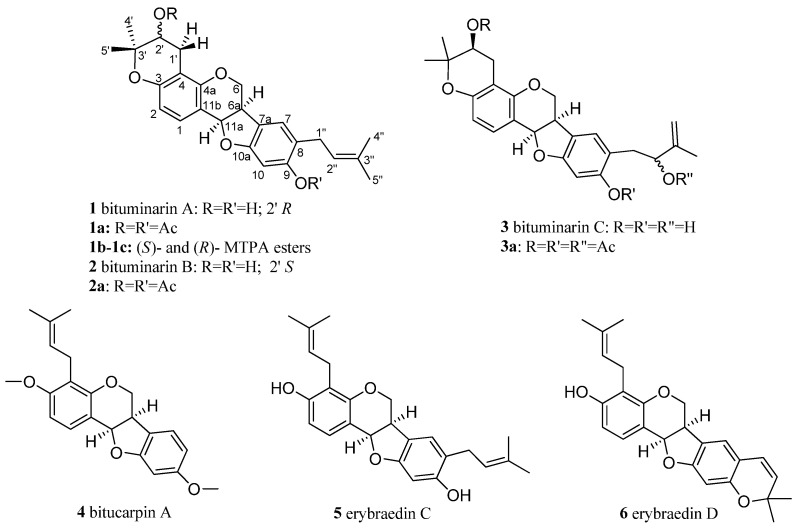
Main pterocarpans isolated from *B. bituminosa*.

**Figure 2:**
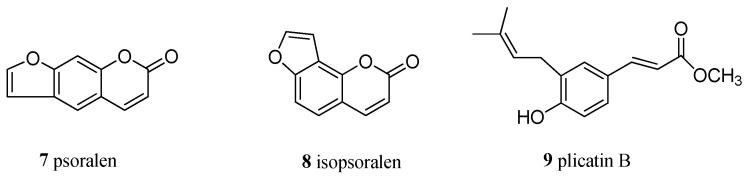
Additional compounds isolated from *B. bituminosa*.

In continuing our studies on Algerian plants used in traditional medicine [15,16,17], we examined the non-polar extract of aerial parts of *B. bituminosa* that was sampled in the region of Fessdis (Batna), in June 2015. The chemical study resulted in the characterization of the pterocarpan fraction including three unprecedented compounds, bituminarins A–C (**1–3**), co-occurring as minor metabolites along with main bitucarpin A (**4**) [4], erybraedin C (**5**) [18,19] and erybraedin D (**6**) [19,20] (Figure 1), already reported from *B. bituminosa*. Additional furanocoumarins, psoralen (**7**) [21,22,23] and isopsoralen [24] (**8**), and plicatin B (**9**) [25] (Figure 2) were also identified in the extract (see Section 4) in agreement with previous reports on *Bituminaria* chemistry. Bitucarpin A has been reported to have anticlastogenic activity in lymphocytes [26], whereas erybraedin C is known to exhibit cytotoxic effects [27] and inhibitory activity on human topoisomerase I [28]. Due to their considerable pharmaceutic interest, additional biological properties of bitucarpin A (**4**) and erybraedin C (**5**) have been explored with regard to their putative antiproliferative activity against SH-SY5Y neuroblastoma cells.

## 2. Results

### 2.1. Chemistry: Structural Elucidation

Known metabolites **4**–**9** were identified by comparison of their spectroscopic data (NMR, MS, [α]_D_) with those reported in the literature [4,18,19,20,21,22,23,24,25]. The structures of new pterocarpans **1**–**3** were determined as follows. Comparison of NMR spectra of bituminarins A–C (**1**–**3**) with those of the co-occurring erybraedin C and D evidenced the presence of the same prenylated pterocarpan core. Compounds **1** and **2** were isomers, whereas compound **3** showed a molecular formula with an additional oxygen atom as was indicated by their molecular peaks in the HR-ESIMS spectra. Even though the NMR assignments reported in the literature for erybraedins were for CDCl_3_ (see Supplementary Materials), bituminarins A–C were also analyzed spectroscopically in other different solvents such as d_6_-acetone (Table 1) and C_6_D_6_ (Section 4) because in CDCl_3_ compounds **1** and **2** were almost unstable and **3** insoluble.

Bituminarin A (**1**) was isolated as an amorphous white powder with a molecular formula C_25_H_28_O_5_ deduced by the molecular peak (M-H)^−^ at *m*/*z* 407.1868 in the HR-ESIMS spectrum (negative mode). The ^1^H NMR spectrum (d_6_-acetone) showed distinctive methine signals at δ_H_ 5.42 (d, *J* = 6.9 Hz, H-11a), 3.52 (m, H-6a) and 4.31 (dd, *J* = 10.5 and 4.6 Hz, H_2_-6α) and 3.57 (dd, *J* = 10.5 and 10.7 Hz, H_2_-6β) ascribable to the protons of the chroman and 2,3-dihydrobenzo[b]furan fused rings of the pterocarpan backbone. Signals at δ_C_ 67.5 (C-6, CH_2_), 40.5 (C-6a, CH), and 79.3 (C-11a, CH) in the ^13^C NMR spectrum supported the presence of a pterocarpan skeleton.

The ^1^H NMR spectrum also showed signals at δ_H_ 7.03 (1H, s, H-7) and 6.31 (1H, s, H-10) and a couple of orto-coupled signals at δ_H_ 7.19 (1H, d, *J* = 8.5 Hz, H-1) and 6.44 (1H, d, *J* = 8.5 Hz, H-2) that were consistent with two tetrasubstituted pterocarpan aromatic rings. The presence of a prenyl chain was indicated by a methylene resonating at δ_H_ 3.21 (1H, dd, *J* = 15.0 and 7.6 Hz, H_2_-1″a) and 3.27 (1H, dd, *J* = 15.0 and 7.2 Hz, H_2_-1″b) coupled to a vinyl proton at δ_H_ 5.31 (1H, br t, *J* = 7.0 Hz, H-2″), which was in turn long-range correlated to two vinyl methyl groups resonating at δ_H_ 1.70 (6H, br s, H_3_-4″ and H_3_-5″). The remaining signals in the ^1^H NMR spectrum accounted for the second prenyl residue attached to the pterocarpan backbone. In particular, a methylene resonating at δ_H_ 2.88 (1H, dd, *J* = 16.9 and 5.5 Hz, H_2_-1′α) and 2.48 (1H, dd, *J* = 16.9 and 7.9 Hz, H_2_-1′β) that was both coupled with a carbinolic methine at δ_H_ 3.74 (1H, m, H-2′) and long-range correlated with two geminal methyl groups at δ_H_ 1.31 (3H, s, H-4′) and 1.21 (3H, s, H-5′) suggested the presence of a 3,3-dimethyl-2-hydroxy pyran moiety, which was evidently derived by cyclization of a prenyl chain, as already reported for prenylated compounds including pterocarpans [29].

**Figure 3:**
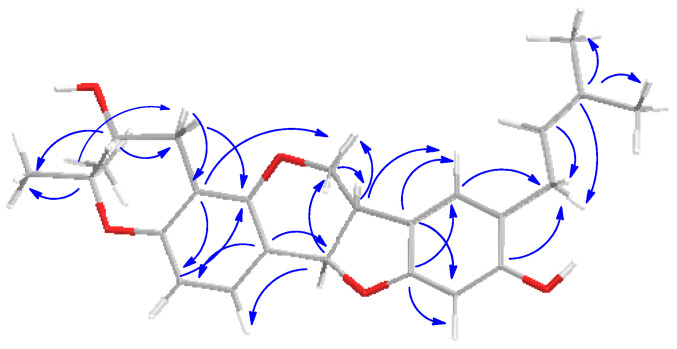
HMBC correlations (from C to H) of bituminarin A (**1**).

**Figure 4:**
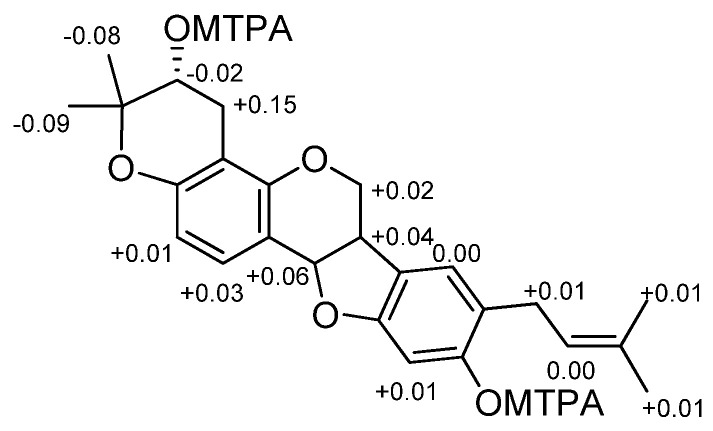
Δδ values (Δδ = δS – δR in ppm) obtained for MTPA esters **1b**/**1c**.

The presence in the ^13^C NMR spectrum of carbon resonances at δ_C_ 78.8 (C, C-3′), 69.6 (CH, C-2′), and 27.1 (CH_2_, C-1′) that could be unequivocally attributed to the carbon atoms of a 3,3-dimethyl-2-hydroxy pyran ring [30] confirmed the structural hypothesis. The HMBC experiment was crucial to establish the position of both pyran ring and prenyl chain (Figure 3). Indeed, diagnostic long-range correlations were observed between H_2_-1′ (δ_H_ 2.88 and 2.48) with C-4 (δ_C_ 108.9) and C-4a (δ_C_ 154.5) as well as between H_2_-1″ (δ_H_ 3.21 and 3.27) with C-7 (δ_C_ 126.0), C-8 (δ_C_ 120.7), and C-9 (δ_C_ 155.9), thus allowing one to locate the pyran ring at C-3/C-4 and the prenyl group at C-8 of the pterocarpan scaffold. Other significant long-range correlations are reported in Figure 1. The coupling constant value (*J* = 6.9 Hz) between angular protons H-6a and H-11a, as well as their NOESY correlation, indicated a cis-junction between the chroman and benzofuran rings as reported for almost all natural pterocarpans. Moreover, the CD spectrum, which showed a positive curve at 286 nm, and the negative specific rotation [α]_D_ −268.0 (c 0.013, CHCl_3_), were consistent with a 6aR,11aR absolute configuration [31]. The absolute configuration of the stereogenic center C-2′ was deduced to be R by applying the modified Mosher method (Figure 4).

**Figure 5:**
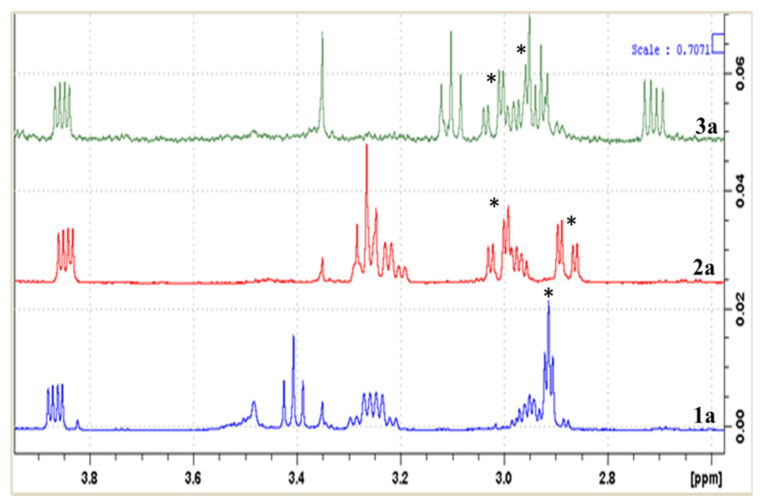
Enlarged ^1^H NMR region (3.95–2.60 ppm) for acetylated bitumarins **1a**–**3a**. The signal pattern of H_2_-1′ methylene is marked with an asterisk in all three spectra.

Bituminarin B (**2**) exhibited strong similarities with bituminarin A (**1**). It had the same molecular formula C_25_H_28_O_5_ as deduced by the peak at *m*/*z* 407.1878 in the HR-ESIMS spectrum (negative mode). Signals in the ^1^H NMR and ^13^C NMR spectra of **2** were almost superimposable with those of **1** implying a strict structural correlation. Analysis of 2D NMR experiments of **2** clearly confirmed that the planar structure is the same as **1**, indicating that the difference between two compounds should be ascribed to stereochemical features. The 6a,11a-cis-junction of **2** was assigned by the J_6a,11a_ (Table 1) as well as NOE interactions analogous to **1**. Further, the CD profile and [α]_D_ = −194.5 (c 0.013, CHCl_3_) indicated the 6aR,11aR absolute configuration also for bituminarin B (**2**) [31]. Consequently, the difference had to be in C-2′ configuration, opposite to that of **1**. In fact, consistent with this, comparison of proton spectra of **1** and **2** showed that the main difference was in the ^1^H NMR pattern of H_2_-1′ methylene signals (Table 1). This difference was more evident by comparing the ^1^H NMR spectra recorded in C_6_D_6_ of the acetyl derivatives of **1** and **2**, compounds **1a** and **2a**, respectively (see Section 4 and Supplementary Materials). In compound **1a**, H_2_-1′ methylene resonated as a broad triplet at δ_H_ 2.91 (2H, *J* = 4.8 Hz), whereas in compound **2a** these proton signals were split into two double doublets at δ_H_ 2.87 (1H, *J* = 17.7, 4.7 Hz) and 3.01 (1H, *J* = 17.7, 5.3 Hz) (Figure 5), in agreement with a different orientation of 2′-OH group. Therefore, bituminarin B (**2**) was the 2′-epimer of bituminarin A (**1**). Complete NMR assignment of **2** is reported in Table 1 (CD_3_COCD_3_) and in C_6_D_6_ (Section 4).

The structure of bituminarin C (**3**) was revealed to be slightly different from **1** and **2**. The molecular formula C_25_H_28_O_6_ that was deduced by the peak M-H^−^ at *m*/*z* 423.1824 in the HR-ESIMS spectrum (negative mode) exhibited an additional oxygen atom with respect to **1** and **2**. The ^1^H NMR spectrum (Table 1) showed signals attributable to the pterocarpan core fused with a 3,3-dimethyl-2-hydroxy pyran ring, the same as **1** and **2**, whereas differences were observed in the resonances due to the second prenyl chain linked at C-8. The spin system observed for the C-1″/C-5″ fragment was constituted by a methylene resonating at δ_H_ 2.78 (2H, m, overlapped signal, H_2_-1″), which was coupled to a carbinolic methine at δ_H_ 4.32 (1H, m, H-2″) that was in turn long-range correlated with both a vinyl methyl at δ_H_ 1.78 (3H, br s, H_3_-5″) and an exomethylene at δ_H_ 4.94 (1H, br s, H_2_-4″a) and 4.77 (1H, br s, H_2_-4″b). These data along with the corresponding ^13^C NMR resonances [δ_C_ 38.6 (CH_2_, C-1″), 77.4 (CH, C-2″), 148.6 (C, C-3″), 110.4 (CH_2_, C-4″), and 18.5 (CH_3_, C-5″)] indicated the presence in the structure of a 3-en-2-ol prenyl chain. Analysis of 2D NMR (^1^H-^1^H COSY, HSQC and HMBC) experiments allowed full proton and carbon assignment as reported in Table 1, also confirming the position of modified prenyl chains as reported in structure **3**. The stereochemistry at C-6a and C-11a stereogenic centers was the same as **1** and **2**, as was expected. The cis-junction was supported by the J_6a,11a_ value (7.0 Hz) and NOE interactions whereas the R,R absolute configurations at C-6a and C-11a were inferred by both the CD curve [31], and the negative specific rotation [α]_D_ −71.5 (c 0.05, CHCl_3_). The absolute configuration at C-2′ in bituminarin C (**3**) was determined by comparing the ^1^H NMR pattern of H_2_-1′ of the corresponding acetyl derivative **3a** with H_2_-1′ signals in compounds **1a** and **2a** exhibiting opposite C-2′ configuration (Figure 5).

The signal multiplicity of the methylene H_2_-1′ in compound **3a** appeared similar with those of compound **2a**, thus suggesting the same S configuration at C-2′, whereas the configuration at C-2″ remained unassigned.

### 2.2. Biological Activity: Antiproliferative Activity of Bitucarpin A (***4***) and Erybraedin C (***5***) on Neuroblastoma SH-SY5Y Cells

**Figure 6:**
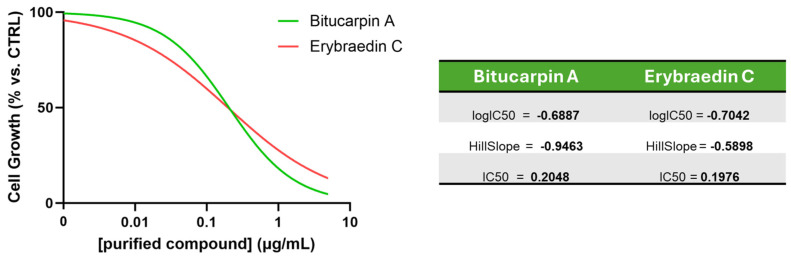
SH-SY5Y cell growth assay. IC_50_ values are measured following 24 h of cell treatment with bitucarpin A or erybraedin C at dose ranging from 0.01 µg/mL to 5 µg/mL in comparison to untreated, control (CTRL) cells.

**Figure 7:**
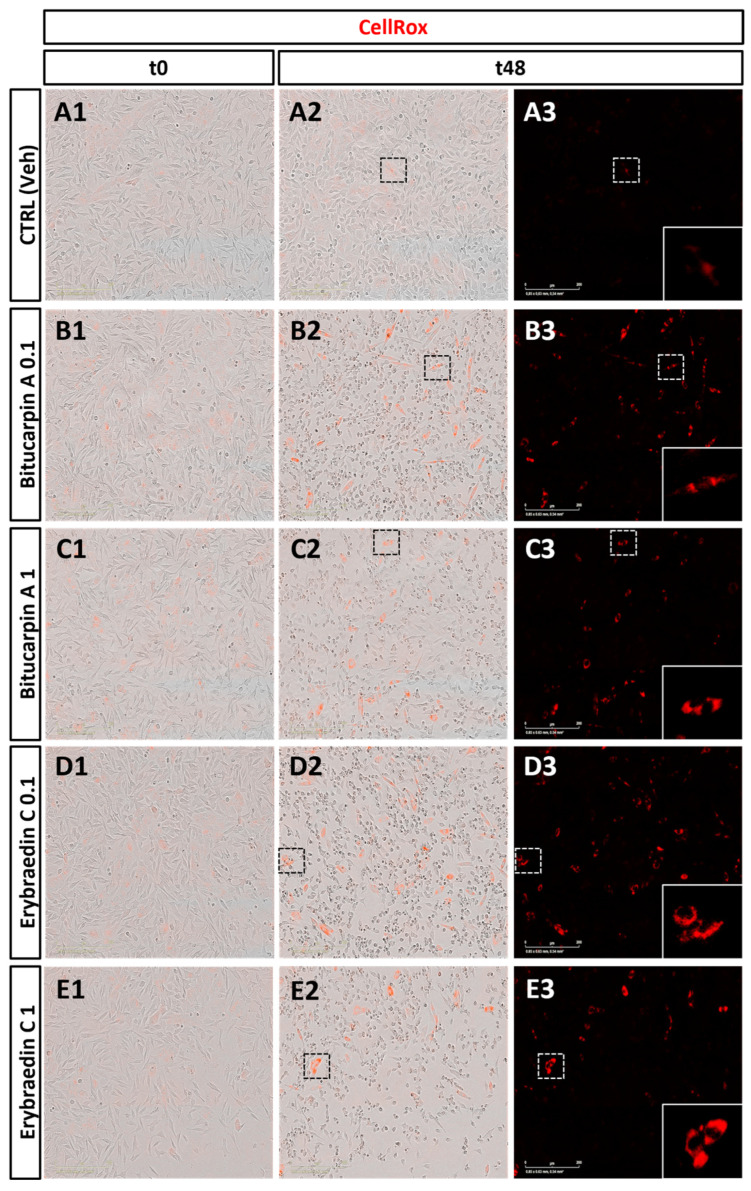
Phase contrast images (20× magnification) of CellRox labelled live SH-SY5Y human neuroblastoma cells at two different time points (t0 and t48). (**A1**–**A3**) Control. (**B1**,**B2**,**C1**,**C2**) Treatment with bitucarpin A 0.1 µg/mL/1 µg/mL. (**D1**,**D2**,**E1**,**E2**) Treatment with erybraedin C 0.1 µg/mL/1 µg/mL. (**A3**,**B3**,**C3**,**D3**,**E3**) Single red channel acquisition showing an increment in the oxidative stress response to 1 µg/mL of bitucarpin A or to both concentrations of erybraedin C compared to vehicle (**A3**). ((**A3**–**E3**) lower right boxes) Magnification of the respective white dotted area indicating a detailed group of SH-SY5Y cells (scale bars = 200 μm).

The biological properties of bitucarpin A (**4**) and erybraedin C (**5**) have been evaluated on neuroblastoma SH-SY5Y cells, that to the best of our knowledge have never been tested with pterocarpan compounds. First, with the aim at establishing the range of effective concentrations for the in vitro studies, a cell growth dose-response bioassay allowed us to determine the IC_50_ of both bitucarpin A and erybraedin C. For this purpose, cell growth percentage was measured by a live-cell scan assay performed through the Zoom Incucyte system after 24 h of treatment with **4** and **5**, both at log-fold concentrations establishing 100% of the cell growth from the untreated cells (Figure 6). This experiment aided in establishing IC_50_ = 0.2048 µg/mL and IC_50_ = 0.1976 µg/mL for bitucarpin A and erybraedin C, respectively. Successively, the effects on ROS production in SH-SY5Y cell line induced by bitucarpin A (**4**) and erybraedin C (**5**), both tested at 1 µg/mL and 0.1 µg/mL, were measured by CellRox assay in the same Zoom Incucyte live-cell scan system. Data were collected every two hours over 48 h of cell incubation by comparing with the time-matched cells incubated without pterocarpans (i.e., vehicle alone), or with LPS as positive control of CellRox induction (Figure 7).

**Figure 8:**
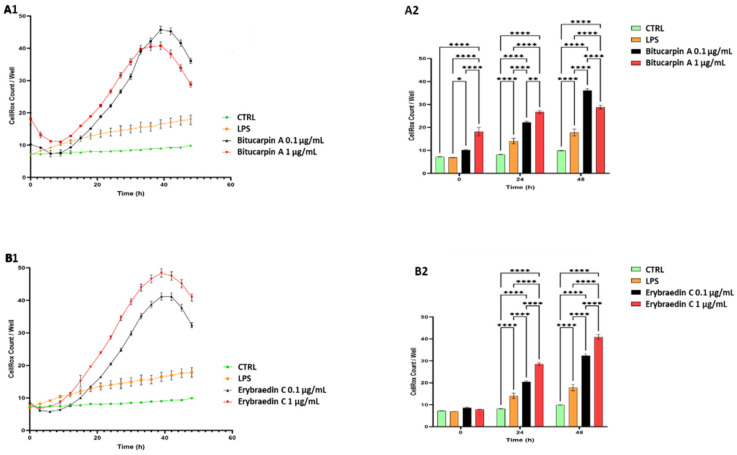
Time course analysis (48 h) of CellRox response to oxidative stress in neuroblastoma SH-SY5Y cell line. (**A1**) Line-plot representing CellRox reactivity of cells treated with or without bitucarpin A (0.1 and 1 µg/mL) in comparison to LPS treatment. (**B1**) Cells treated with or without erybraedin C (0.1 and 1 µg/mL) in comparison to LPS treatment. (**A2**) Bar-plot (t0, t24, and t48 timepoints) quantitative analysis of CellRox reactivity of bitucarpin A treatment (at 0.1 µg/mL and 1 µg/mL) vs. vehicle. (**B2**) Bar-plot quantitative analysis (t0, t24 and t48) of CellRox reactivity of erybraedin C treatment (0.1 µg/mL and 1 µg/mL) vs. vehicle. The *y* axis is the number of CellRox-positive cells per well (cells/well), while the *x* axis is the time in hours. Two-way ANOVA with Tukey post-hoc test was used to assess statistical significance (* *p* < 0.05, ** *p* < 0.01, **** *p* < 0.0001; error bars represent mean ± SEM; *n* = 48).

According to the literature [12,27,32], a time- and dose-dependent induction of the CellRox production was observed in SH-SY5H human neuroblastoma cell line treated with **4** and **5** at both concentrations (Figure 8). In particular, the analysis revealed a significant increase in the percentage of CellRox-positive cells after the first 10 h of incubation for both compounds at the higher tested dose. It is noteworthy that, after 32 h of incubation, bitucarpin A showed at 0.1 µg/mL a percentage of positive cells higher than that induced at 1 µg/mL (Figure 8).

**Figure 9:**
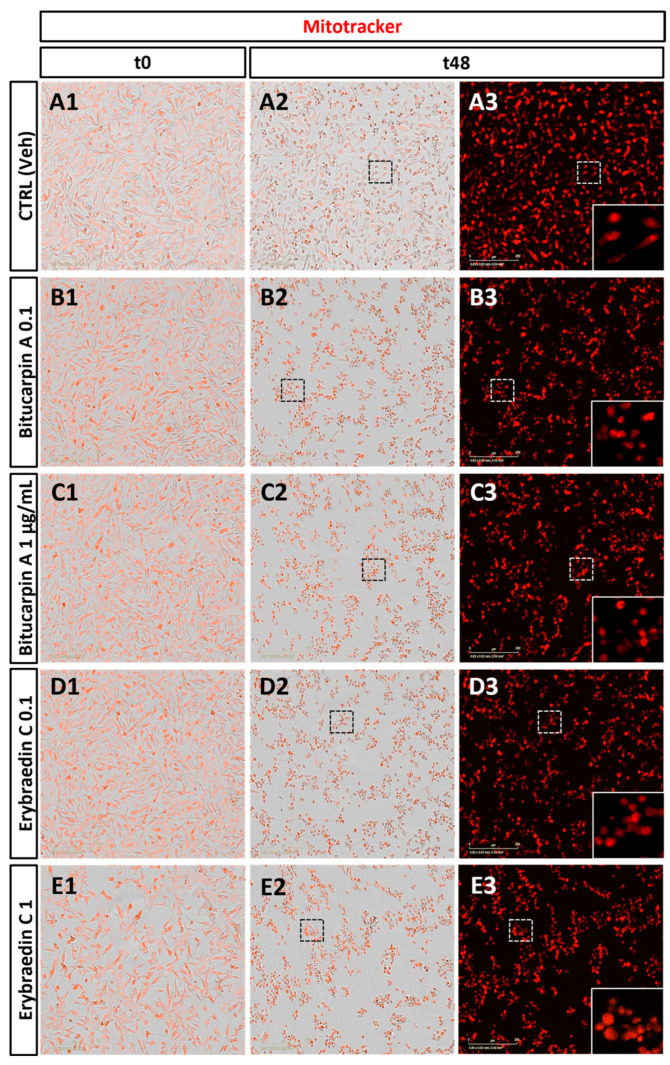
Phase contrast images (20× magnification) of Mitotracker labeled live SH-SY5Y human neuroblastoma cells at two different time points (t0 and t48). (**A1**–**A3**) control; (**B1**,**B2**,**C1**,**C2**) bitucarpin A treatment 0.1 µg/mL/1 µg/mL; (**D1**,**D2**,**E1**,**E2**) erybraedin C treatment 0.1 µg/mL/1 µg/mL; (**A3**,**B3**,**C3**,**D3**,**E3**) single red channel acquisition revealing an increment of oxidative stress response to 1 µg/mL of bitucarpin A or to both concentrations of erybraedin C compared to the vehicle (**A3**); (**A3**–**E3**) lower right boxes: magnification of the respective white dotted area indicating a detailed group of SHSY-5Y cells (scale bars = 200 μm).

**Figure 10:**
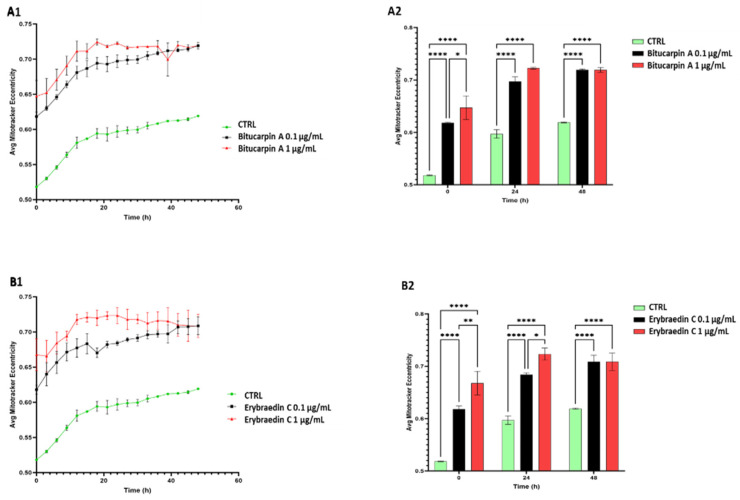
Time course analysis (48 h) of Mitotracker response to oxidative stress in SH-SY5Y cell line. (**A1**) Line-plot graph representing the time-course Mitotracker reactivity of cells treated with or without bitucarpin A (0.1 and 1 µg/mL) in comparison with LPS treatment. (**B1**) Line-plot graph of Mitotracker reactivity of cells treated with or without erybraedin C (0.1 and 1 µg/mL) in comparison with LPS treatment. (**A2**) Bar-plot quantitative analysis (t0, t24 and t48) of mitochondrial eccentricity changes induced by bitucarpin A treatment (at 0.1 µg/mL and 1 µg/mL) vs. control. (**B2**) Bar-plot quantitative analysis (t0, t24 and t48) of mitochondrial eccentricity changes induced by erybraedin C treatment (at 0.1 µg/mL and 1 µg/mL) vs. control. The *y* axis is eccentricity defined in an ellipse as the ratio of the distance between its center and either of its two foci (from 1 for circular shapes to 0 for straight lines); the *x* axis is the time in hours. Two-way ANOVA with Tukey post-hoc test was used to assess statistical significance (* *p* < 0.05, ** *p* < 0.01, **** *p* < 0.0001; error bars represent mean ± SEM; *n* = 48).

**Figure 11:**
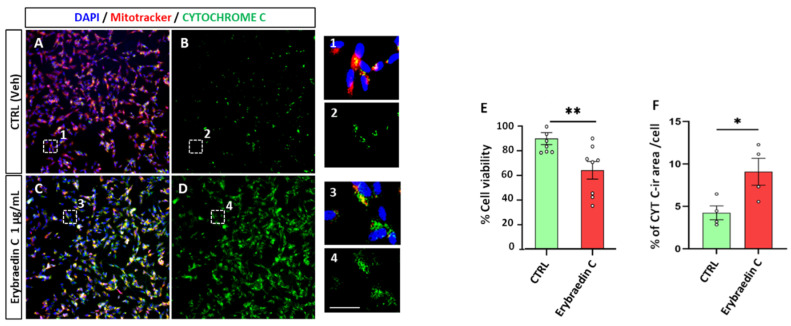
Erybraedin C affects Mitotracker and cytochrome C expression in SH-SY5Y cells. (**A**–**D**) Images of triple Mitotracker (Red)/cytochrome C (Green)/DAPI (Blue) labeled vehicle-treated cells (A merge, and B single cytochrome C immunolabeling) in comparison to 24 h treatment with erybraedin C (1 µg/mL) (C merge, and D single cytochrome C immunolabeling). Dotted boxes inside each picture represent the respective field acquired with high magnification showing cellular details of SH-SY5Y cells in 1–4. Scale bar = 100 μm in A–D, and 10 μm in 1–4. (**E**) Bar-plot analysis of the mean of percentage of live cells before and after erybraedin C treatment as revealed by live and dead assay. (**F**) Bar-plot analysis of the mean of percentage of the cytochrome C-positive immunolabeled area/cell. Two-way ANOVA with Tukey post-hoc test was used to assess statistical significance (* *p* < 0.05; ** *p* < 0.01; error bars represent mean ± SEM).

With the aim of deeply investigating putative apoptotic induced effects by bitucarpin A (**4**) and erybraedin C (**5**), mitochondrial dynamics that have significance in the apoptotic signal transduction associated with cytochrome C release [33,34,35] were also analyzed. For this purpose, the “eccentricity index” variations induced by compounds **4** and **5** were measured. An eccentricity index value of 0.5 represents the morphological measure of the ideal circular shape typical of mitochondria in healthy cells [36,37] and deviation from this reference value indicates a cellular morphological hallmark predictive of apoptotic events [38,39]. Mitochondrial dynamics were characterized, with single-organelle resolution, by live-cell microscopy associated with both time-lapse Zoom Incucyte image analysis software (version 20181.1.6628.28170) and supervised machine learning (Figure 9). Rhodamine-based staining dye MitoTracker was used in live cells treated with bitucarpin A and erybraedin C, over 48 h of incubation at 0.1 µg/mL and 1.0 µg/mL for both compounds. This analysis revealed a significant increase in the eccentricity mitochondrial index, highlighting an enhancement of the value close to 0.75 from the first 15 h of incubation for both bitucarpin A and erybraedin C at 1.0 µg/mL (Figure 10A1,B1). Additionally, putative apoptotic pathways involvedin cell damage induced by erybraedin C were further investigated in SH-SY5Y. Immunostaining of cytochrome C expression highlighted a morphological change of cytochrome C localization in cells treated with 1.0 μg/mL erybraedin C, observing a puncta-like distribution in the vehicle-treated cells vs. a widespread cytosolic localization in treated cells (Figure 11A–D and high magnification of boxed areas 1–4). These morphological changes were accompanied by both a significant reduction in the percentage of live cells (Figure 11E) and quantitative increase in cellular area expressing cytochrome C immunolabeling (Figure 11F).

**Figure 12:**
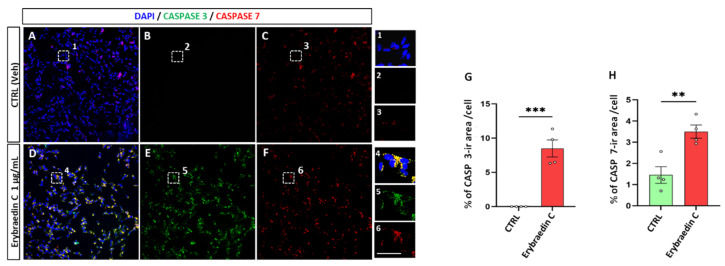
Erybraedin C (1 µg/mL) affects Caspase 3 and 7 expressions in SH-SY5Y cell culture. (**A**–**F**) Dotted boxes inside each picture represent the respective field acquired with high magnification showing increase in caspase 3 (Green) or caspase 7 (Red) immunolabeling in 1–6. Scale bar = 100 μm in (**A**–**F**) and 10 μm in 1–6. (**G**,**H**) Bar-plot analysis of the mean of percentage of caspase 3 or 7 immunolabeled area/cell. Two-way ANOVA with Tukey post-hoc test was used to assess statistical significance (** *p* < 0.01; *** *p* < 0.001; error bars represent mean ± SEM).

As under normal conditions cytochrome C resides in the mitochondria, exodus into the cytoplasm is considered as a marker of mitochondrial damage and apoptotic cell death by activation of key players, e.g., caspase-9 and the apoptotic effector caspases-3, 6, and 7 [33,34,35]. To assess cell death by apoptosis, caspase-3 (Cas-3) and caspase-7 (Cas-7) expression was measured by immunolabeling SH-SY5Y cells treated with erybraedin C. A significant increase in the percentage of the immunolabeled cellular area was observed for both caspases (Figure 12).

**Figure 13:**
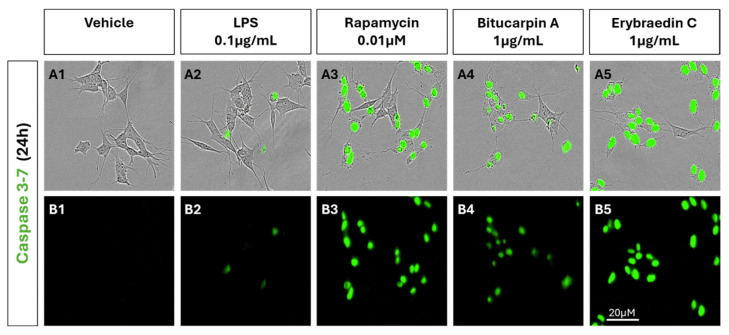
Representative micrographs from Zoom Incucyte live scan analysis of SH-SY5Y cells, untreated (**A1**) or treated with LPS 0.1 μg/mL (**A2**), rapamycin 0.01 μM (**A3**), bitucarpin A (**A4**) and erybraedin C (**A5**) both at 1 μg/mL for 24 h. Caspases 3 and 7 were evaluated using Incucyte live dyes Caspase-3/7 for Apoptosis in green, highlighted in the bottom part of the image (**B1**–**B5**).

The induction of apoptosis in neuroblastoma cells was analysed by live cell scanning assay of caspase 3–7 reactivity (Figure 13). The effect of erybraedin C or bitucarpin A (1 µg/mL) was compared with rapamycin (0.01 µM) in order to confirm the induction of the pro-apoptotic cascade with a known anticancer drug. While in untreated (VEH) and in treated LPS the levels of capsase 3 and 7 remain low, in the treatment with erybraedin C and bitucarpin A, as well as in that with rapamycin, we observe an increase in cell death and a strong activation of caspases 3 and 7.

## 3. Discussion and Conclusions

The chemical characterization of the pterocarpan fraction from the non-polar extract of *B. bituminosa* from Algeria resulted in the isolation and identification of a series of prenyl pterocarpans, including the new bituminarins A–C (**1**–**3**), as minor metabolites, and the main bitucarpin A (**4**) [4], erybraedin C (**5**) [18,19], and erybraedin D (**6**) [19,20] already reported from this species. The new compounds, bituminarins A–C (**1**–**3**), could be enzymatically derived from erybraedin C (**5**) by cyclization of C-4 prenyl group and further oxidation on the double bond to give the 2-hydroxy-3,3-dimethyldihydropyran ring. However, even though they were detected in the early crude extract fraction of the plant, a possible work-up origin could not be excluded for compounds **1**–**3**. Prenyl substituents at C-4 and/or at C-8 of the pterocarpan framework are a distinctive structural feature of this group of metabolites that is scarcely distributed in nature and seems to be typical of *Bituminaria* plants [14]. However, pterocarpans with similar prenyl residues, either linear or cyclized, have been recently reported also from *Erythrina lysistemon* [40] even though the substitution pattern of the pterocarpan nucleus was different from that described for *Bituminaria* metabolites. The presence of prenyl groups seems to enhance the bioactivities of the pterocarpan scaffold [27,41] as indicated by the broad spectrum of biological activities reported for prenylated pterocarpans [14]. In this regard, the bioactivity evaluation of bitucarpin A (**4**) and erybraedin C (**5**) carried out on neuroblastoma SH-SY5Y cells adds further insight into the biomedical potential of these well-known bioactive compounds. Both bitucarpin A and erybraedin C were found to induce a significant reduction of the cell viability in neuroblastoma SH-SY5Y cells, revealing by time-lapse observation a time-dependent efficiency of both compounds to promote ROS production, mitochondria aggregation in a budding-like shape (i.e., eccentricity), and cytochrome C dislocation and spreading from mitochondria to cytoplasm [33,34]. Pterocarpans constitute intermediate metabolites produced by specific redox reactions along the isoflavones biosynthesis [42]. Erybraedin C, due to its phenolic hydroxyl and 4,8 prenyl substitutes, may exhibit a high chain-breaking antioxidant potential. Here, we report that erybraedin C induced a comparable dose-dependent cytotoxicity and dose- and time-dependent apoptosis in human glioblastoma cell line, unrelated to an apparent cell cycle checkpoint arrest. At the molecular level, this process may occur possibly by increasing the concentration of single- and double-stranded DNA breaks according to the previously reported effect of etoposide to stabilize a normally transient covalent DNA-topoisomerase II complex. This process triggers cell cycle arrest and activation of the biochemical cascade of terminal apoptotic events [43,44]. Here, by using immunofluorescence staining, the induction of caspase-3 and caspase 7 was evidenced in concomitance with the pterocarpan-induced release of cytochrome C into the cytosol in cells undergoing apoptosis. These data are in accordance with previous studies reporting the antiproliferative effects of bitucarpin A and erybraedin C on human colon adenocarcinoma cells. In particular, erybraedin C has been proposed to induce DNA damage by stabilizing a transient covalent DNA-topoisomerase II complex [45], that is within diverse cellular stresses known to kill via the mitochondrial pathway of apoptosis [36]. However, further studies on the mode of action underlying pterocarpan-induced apoptosome formation are required to better clarify their effects on the mitochondrial outer membrane permeabilization (MOMP)-induced apoptosis.

## 4. Materials and Methods

### 4.1. General Experimental Procedures

Optical rotations were measured on a Jasco DIP 370 digital polarimeter (Jasco, Lecco, Italy). The CD curves were recorded on a JASCO F815 spectropolarimeter (Jasco, Lecco, Italy). High-resolution mass spectra (HRESIMS) were acquired on a Q-Exactive hybrid quadrupole-orbitrap mass spectrometer (Thermo Scientific, San Jose, CA, USA). NMR experiments were recorded at the ICB-NMR Service Centre. Chemical shift values are reported in ppm and referenced to internal signals of residual protons (CD_3_COCD_3_, δ 2.04 for H-atom, δ 29.8 for carbon; C_6_D_6_, δ 7.15 for H-atom, δ 128.0 for carbon). The 1D and 2D NMR spectra were acquired on a Bruker Avance-400 spectrometer (Bruker, Milan, Italy) using an inverse probe fitted with a gradient along the *Z* axis, on a Bruker Avance III HD 400 MHz spectrometer equipped with a CryoProbe Prodigy, and on a DRX 600 spectrometer equipped with a three-channel inverse (TCI) CryoProbe (Bruker, Milan, Italy). Analytical and preparative TLC were performed on precoated SiO_2_ plates (Merck Kieselgel 60 F254, 0.25 and 0.5 mm) (Merck, Milan, Italy), with detection provided by UV light (254 nm) and by spraying with CeSO_4_ reagent followed by heating (120 °C). SiO_2_ column chromatography was performed using Merck Kieselgel 60 powder (0.063–0.200 mm) (Merck, Milan, Italy). HPLC separation was carried out on a C18 semipreparative column (Ascentis, 250 × 10 mm, 5 μm, Supelco, Milan, Italy) using a Shimadzu HPLC system equipped with an LC-10AD Shimadzu liquid chromatograph and an SPD-10A wavelength detector (210 and 254 nm) (Shimadzu, Tokyo, Japan).

### 4.2. Plant Material

*Bituminaria bituminosa* was collected in the region of Fessdis (Batna), in June 2015. The plant was identified by Prof. Bachir Oudjehih of the Department of Agronomy of the Institute of Veterinary and Agronomic Science of the University of Batna 1. A voucher specimen (PB) was deposited at the Herbarium of the University of Batna-1, Batna, Algeria.

### 4.3. Extraction and Isolation

Air-dried aerial parts of *B. bituminosa* (1 kg) were powdered and macerated with 70% aqueous EtOH (2 × 10 L) for 72 h at room temperature. After filtration, the combined organic eluates were evaporated under reduced pressure to give a crude residue, which was suspended in water (500 mL) and successively extracted with 400 mL of petroleum ether (PE) three times, then 400 mL of ethyl acetate (EtOAc) and finally with 400 mL of *n*-butanol (*n*-BuOH). The organic phases were concentrated under reduced pressure to give the corresponding extracts: PE (4.0 g), EtOAc (10.8 g) and *n*-BuOH (24.0 g). TLC chromatography analysis of these three extracts revealed a very rich metabolite content in the PE extract. Therefore, a portion (2 g) of this latter was fractionated by silica-gel column chromatography by eluting first with a gradient of diethyl ether (Et_2_O) in petroleum ether (from 0 to 100%) to obtain 13 fractions (C2-1–C2-13), and after with a gradient of methanol (MeOH) in CHCl_3_ (from 0 to 50%) to obtain a further five fractions (C2-14–C2-18). Selected fractions (C2-4, C2-6, C2-7, C2-8) were taken into consideration after TLC chromatography analysis and preliminary ^1^H NMR inspection. A portion (10 mg) of fraction C2-4 (110 mg) was loaded into a semipreparative TLC plate and developed with PE/Et_2_O, 7:3 to afford 5.2 mg of a UV absorbing pure compound (R*f* 0.85) that was identified, by NMR and MS analysis, as bitucarpin A (**4**). Purification of fraction C2-6 (305 mg) was performed on SiO_2_ gel column, using as eluent system a gradient of Et_2_O in PE (from 0 to 90%) and finally a mixture of CHCl_3_/MeOH 9:1, obtaining 18 fractions (C5-1–C5-18). An aliquot (50 mg) of fraction C5-5 (81.5 mg) was further purified by a SiO_2_ gel column eluting with a gradient of CHCl_3_ in PE (from 10 to 80%) to obtain seven fractions (C6-1–C6-7). A half portion of fraction C6-4 (40 mg) was loaded into two semipreparative TLC plates and developed with CHCl_3_/acetone 95:5 to give two UV bands at R*f* 0.9 and 0.8 that were identified by NMR and MS as isopsoralen (**8**, 4.5 mg) and plicatin B (**9**, 2.3 mg), respectively. Fraction C2-7 (240 mg) was subjected to a further fractionation on the SiO_2_ gel column using as the eluent system a gradient of Et_2_O in PE (from 10 to 100%) and finally CHCl_3_/MeOH 95:5. Twenty fractions (C8-1–C8-20) were collected and analyzed by TLC. Selected fractions from this column were submitted to ^1^H NMR. Fraction C8-5 (4.0 mg) contained a pure compound that was identified as erybraedin D (**6**), whereas fractions from C8-6 to C8-9 contained psoralen (**7**) in mixture with fatty acids. Erybraedin C (**5**) was the main compound present in fractions C8-11 (16.6 mg) and C8-12 (30.9 mg). Purification of 10.0 mg of fraction C8-12 by semipreparative TLC (PE/Et_2_O, 1:1) afforded 4.5 mg of pure erybraedin C (**5**, R*f* 0.45). Fraction C2-8 (91.6 mg) was fractionated by SiO_2_ gel column chromatography using a gradient of Et_2_O in hexane (from 10 to 60%) and successively of MeOH in CHCl_3_ (from 0 to 5%) to afford fourteen fractions (C10-1–C10-14). Fraction C10-2 (2.0 mg) contained an additional amount of erybraedin D (**6**, 1.0 mg) whereas fraction C10-4 (5.5 mg) was further purified in semipreparative TLC (PE/Et_2_O, 6:4) to obtain 1.1 mg of psoralen (**7**). Fraction C10-7 (12.0 mg) was subjected to HPLC purification using an RP18 semipreparative column eluting in isocratic mode with CH_3_CN/H_2_O 75:25 (flow 2.0 mL/min) to give three main peaks. The peak eluted at R*t* 21.4 min was identified after NMR analysis and MS as the new compound bituminarin A (**1**, 0.7 mg), the peak eluted at R*t* 23.9 min was identified as the new compound bituminarin B (**2**, 0.7 mg), whereas the peak at R*t* 37.9 min was identified as erybraedin C (**5**, 0.5 mg). Fraction 10-10 (6.7 mg) was purified by HPLC on the RP18 semipreparative column in isocratic mode with CH_3_CN/H_2_O 60: 40 (flow 2.0 mL/min) to give three main peaks. Only the peak eluted at R*t* 15.7 min was pure and identified as the new compound bituminarin C (**3**, 1.0 mg).

Bituminarin A (**1**)

White powder; [α]_D_ −268.0 (*c* 0.013, CHCl_3_); ECD (*c* 4.08 × 10^−4^ M, MeOH) *λ*_max_ (Δ*ε*) 290 (+1.3), 238 (−3.93) nm; ^1^H and ^13^C NMR data in acetone-*d*_6_ see Table 1. ^1^H NMR (C_6_D_6_), δ 7.33 (1H, d, *J* = 8.5 Hz, H-1), 6.77 (1H, s, H-7), 6.75 (1H, d, *J* = 8.5 Hz, H-2), 6.20 (1H, s, H-10), 5.36 (1H, br t, *J* = 7.2 Hz, H-2″), 5.25 (1H, d, *J* = 7.0 Hz, H-11a), 3.98 (1H, dd, *J* = 10.9, 5.0 Hz, H_2_-6α), 3.58 (1H, dd, *J* = 10.9, 10.9 Hz, H_2_-6β), 3.35 (1H, app t, *J* = 5.0 Hz, H-2′), 3.32 (1H, dd, *J* = 15.7, 7.3 Hz, H-1″a), 3.27 (1H, dd, *J* = 15.7, 7.2 Hz, H-1″b), 3.06 (1H, ddd, *J* = 10.9, 7.0, 5.0 Hz, H-6a), 2.78 (1H, dd, *J* = 17.0, 5.5 Hz, H_2_-1′α), 2.64 (1H, dd, *J* = 17.0, 6.8 Hz, H_2_-1′β), 1.61 (3H, s, H-4″), 1.58 (3H, s, H-5″), 1.18 (3H, s, H-5′), 1.08 (3H, s, H-4′); ^13^C NMR (C_6_D_6_), δ 159.4 (C-10a, C), 155.3 (C-9, C), 154.3 (C-4a, C), 153.8 (C-3, C), 132.4 (C-3″, C), 128.9 (C-1, CH), 124.6 (C-7, CH), 123.2 (C-2″, CH), 118.8 (C-6b and C-11b, both C), 119.8 (C-8, C), 111.8 (C-11b, C), 110.7(C-2, CH), 108.1 (C-4, C), 98.3 (C-10, CH), 78.7 (C-11a, CH), 76.9 (C-3′, C), 69.2 (C-2′, CH), 66.4 (C-6, CH_2_), 39.7 (C-6a, CH), 28.8 (C-1″, CH_2_), 26.7 (C-1′,CH_2_), 25.4 (C-4″, CH_3_), 24.7 (C-4′, CH_3_), 20.8 (C-5′, CH_3_), 17.4 (C-5″, CH_3_); HR-(-)-ESIMS *m*/*z* 407.1868 [M-H]^−^ (calcd 407.1864 for C_25_H_27_O_5_).

Bituminarin B (**2**)

White powder; [α]_D_ −194.5 (*c* 0.013, CHCl_3_); ECD (*c* 1.63 × 10^−4^ M, MeOH) *λ*max (Δ*ε*) 290 (+3.1), 238 (−9.24) nm; ^1^H and ^13^C NMR data in acetone-*d*6 see Table 1. ^1^H NMR (C_6_D_6_), δ 7.33 (1H, d, *J* = 8.5 Hz, H-1), 6.76 (1H, s, H-7), 6.75 (1H, d, *J* = 8.5 Hz, H-2), 6.20 (1H, s, H-10), 5.36 (1H, br t, *J* = 7.0 Hz, H-2″), 5.25 (1H, d, *J* = 7.0 Hz, H-11a), 3.99 (1H, dd, *J* = 10.9, 5.2 Hz, H_2_-6α), 3.55 (1H, dd, *J* = 10.9, 10.9 Hz, H_2_-6β), 3.39 (1H, app t, *J* = 5.7 Hz, H-2′), 3.32 (1H, dd, *J* = 15.6, 7.0 Hz, H-1″a), 3.26 (1H, dd, *J* = 15.6, 7.8 Hz, H-1″b), 3.06 (1H, ddd, *J* = 10.9, 7.0, 5.2 Hz, H-6a), 2.84 (1H, dd, *J* = 17.3, 5.4 Hz, H_2_-1′α), 2.62 (1H, dd, *J* = 17.3, 6.2 Hz, H_2_-1′β), 1.61 (3H, s, H-4″), 1.58 (3H, s, H-5″), 1.17 (3H, s, H-5′), 1.08 (3H, s, H-4′); ^13^C NMR (C_6_D_6_), δ 159.7 (C-10a, C), 155.4 (C-9, C), 154.3 (C-4a, C), 153.6 (C-3, C), 132.7 (C-3″, C), 129.2 (C-1, CH), 125.1 (C-7, CH), 123.0 (C-2″, CH), 119.4 (C-8, C), 118.8 (C-6b), 112.2 (C-11b, C), 110.6 (C-2, CH), 107.9 (C-4, C), 98.3 (C-10, CH), 78.9 (C-11a, CH), 76.9 (C-3′, C), 69.1 (C-2′, CH), 66.5 (C-6, CH_2_), 39.9 (C-6a, CH), 29.1 (C-1″, CH_2_), 26.5 (C-1′,CH_2_), 25.9 (C-4″, CH_3_), 24.7 (C-4′, CH_3_), 20.9 (C-5′, CH_3_), 17.5 (C-5″, CH_3_); HR-(-)-ESIMS *m*/*z* 407.1878 [M-H]^−^ (calcd 407.1864 for C_25_H_27_O_5_).

Bituminarin C (**3**)

White powder; [α]_D_ −71.5 (*c* 0.05, CHCl_3_); ECD (*c* 5.5 × 10^−4^ M, MeOH) *λ*max (Δ*ε*) 290 (+2.55), 239 (−6.48) nm; ^1^H and ^13^C NMR data in acetone-*d*_6_ see Table 1. ^1^H NMR (C_6_D_6_), δ 7.29 (1H, d, *J* = 8.4 Hz, H-1), 6.51 (1H, s, H-7), 6.74 (1H, d, *J* = 8.5 Hz, H-2), 6.85 (1H, s, H-10), 5.22 (1H, d, *J* = 7.1 Hz, H-11a), 4.72 (1H, s, H-4″a), 4.64 (1H, s, H-4″b), 4.06 (1H, dd, *J* = 11.1, 5.2 Hz, H_2_-6α), 3.75 (1H, br d, *J* = 8.0 Hz, H-2″), 3.61 (1H, dd, *J* = 11.1, 11.1 Hz, H_2_-6β), 3.39 (1H, app t, *J* = 5.7 Hz, H-2′), 3.09 (1H, ddd, *J* = 11.1, 7.1, 5.2 Hz, H-6a), 2.87 (1H, dd, *J* = 17.3, 5.2 Hz, H_2_-1′α), 2.73 (1H, dd, *J* = 15.6, 7.0 Hz, H-1″a), 2.63 (1H, dd, *J* = 17.3, 6.2 Hz, H_2_-1′β), 2.54 (1H, dd, *J* = 15.6, 7.8 Hz, H-1″b), 1.50 (3H, s, H-5″), 1.17 (3H, s, H-5′), 1.08 (3H, s, H-4′); ^13^C NMR (C_6_D_6_), δ 160.3 (C-10a, C), 158.4 (C-9, C), 155.5 (C-4a, C), 147.6 (C-3″, C), 130.0 (C-1, C), 126.7 (C-7, CH), 118.8 (C-6b, C), 118.0 (C-8, C), 112.2 (C-11b, C), 110.8 (C-2, CH), 111.4 (C-4″, CH_2_), 108.2 (C-4, C), 100.3 (C-10, CH), 78.9 (C-2″, CH), 78.3 (C-11a, CH), 77.8 (C-3′, C), 69.3 (C-2′, CH), 66.8 (C-6, CH_2_), 40.2 (C-6a, CH), 38.1 (C-1″, CH_2_), 26.8 (C-1′, CH_2_), 24.8 (C-4′, CH_3_), 21.3 (C-5′, CH_3_), 18.1 (C-4″, CH_3_); HR-(-)-ESIMS *m*/*z* 423.1824 [M-H]^−^ (calcd 423.1813 for C_25_H_27_O_6_).

### 4.4. Acetylation of Bituminarins ***A***–***C***

Compounds **1**–**3** (0.2 mg) were separately reacted with acetic anhydride in 1 mL of anhydrous CH_2_Cl_2_ and a catalytic amount of DMAP, and the solutions were stirred overnight at room temperature. After removing the solvent under vacuum, the reaction products were loaded into three SiO_2_ pipette Pasteur packed in petroleum ether and eluted with increasing amount of petroleum ether/diethyl ether to obtain compounds **1a**–**3a**.

Compound **1a**: ^1^H NMR (C_6_D_6_), δ 7.27 (1H, d, overlapped, H-1), 6.85 (1H, s, H-7), 6.75 (1H, d, *J* = 8.5 Hz, H-2), 6.74 (1H, s, H-10), 5.34 (1H, br t, *J* = 7.2 Hz, H-2″), 5.13 (1H, d, *J* = 7.2 Hz, H-11a), 5.07 (1H, app t, *J* = 5.0 Hz, H-2′), 3.87 (1H, dd, *J* = 11.0, 5.2 Hz, H_2_-6α), 3.41 (1H, dd, *J* = 11.0, 11.0 Hz, H_2_-6β), 3.28 (1H, dd, *J* = 15.7, 7.0 Hz, H-1″b), 3.23 (1H, dd, *J* = 15.7, 7.4 Hz, H-1″a), 2.95 (1H, ddd, *J* = 11.0, 7.2, 5.2 Hz, H-6a), 2.91 (2H, br t, *J* = 4.8 Hz, H_2_-1′), 1.73 (3H, s, COCH_3_-9), 1.65 (3H, s, H-4″), 1.59 (3H, s, H-5″), 1.53 (3H, s, (3H, s, COCH_3_-3′), 1.22 (3H, s, H-5′), 0.98 (3H, s, H-4′); ^13^C NMR (C_6_D_6_), δ 169.8 (COCH_3_-3′, C), 168.5 (COCH_3_-9, C), 158.8 (C-10a, C), 154.4 (C-4a, C), 150.1 (C-9, C), 132.2 (C-3″, C), 129.5 (C-1, CH), 125.9 (C-6b, C), 125.2 (C-7, CH), 122.8 (C-2″, CH), 111.8 (C-11b, C), 110.7(C-2, CH), 107.5 (C-4, C), 104.9 (C-10, CH), 78.8 (C-11a, CH), 75.1 (C-3′, C), 70.3 (C-2′, CH), 66.1 (C-6, CH_2_), 39.9 (C-6a, CH), 28.9 (C-1″, CH_2_), 25.5 (C-4″, CH_3_), 24.2 (C-4′, CH_3_), 23.8 (C-1′,CH_2_), 22.8 (C-5′, CH_3_), 20.3 (COCH_3_-2′, CH_3_), 20.0 (COCH_3_-9, CH_3_), 17.7 (C-5″, CH_3_); HR-(+)-ESIMS *m*/*z* 515.2027 [M + Na]^+^ (calcd 515.2051 for C_29_H_32_O_7_Na).

Compound **2a**: ^1^H NMR (C_6_D_6_), δ 7.29 (1H, d, overlapped, H-1), 6.84 (1H, s, H-7), 6.76 (1H, d, *J* = 8.5 Hz, H-2), 6.73 (1H, s, H-10), 5.33 (1H, br t, *J* = 7.2 Hz, H-2″), 5.15 (1H, d, *J* = 7.2 Hz, H-11a), 5.09 (1H, app t, *J* = 5.1 Hz, H-2′), 3.85 (1H, dd, *J* = 11.0, 5.2 Hz, H_2_-6α), 3.27 (1H, dd, *J* = 15.7, 7.0 Hz, H_2_-1″b), 3.26 (1H, dd, *J* = 11.0, 11.0 Hz, H_2_-6β), 3.21 (1H, dd, *J* = 15.7, 7.4 Hz, H_2_-1″a), 3.01 (1H, dd, *J* = 17.7, 5.3 Hz, H_2_-1′α), 2.97 (1H, ddd, *J* = 11.0, 7.2, 5.2 Hz, H-6a), 2.87 (2H, dd, *J* = 17.7, 4.7 Hz, H_2_-1′β), 1.74 (3H, s, COCH_3_-9), 1.65 (3H, s, H-4″), 1.58 (3H, s, H-5″), 1.54 (3H, s, (3H, s, COCH_3_-3′), 1.20 (3H, s, H-5′), 1.04 (3H, s, H-4′); ^13^C NMR (C_6_D_6_), δ 169.8 (COCH_3_-2′, C), 168.5 (COCH_3_-9, C), 159.5 (C-10a, C), 154.2 (C-4a, C), 150.4 (C-9, C9), 132.3 (C-3″, C), 130.0 (C-1, CH), 125.9 (C-6b, C), 125.5 (C-7, CH), 123.5 (C-2″, CH), 111.3 (C-11b, C), 110.0 (C-2, CH), 108.0 (C-4, C), 104.6 (C-10, CH), 79.2 (C-11a, CH), 75.3 (C-3′, C), 70.7 (C-2′, CH), 66.2 (C-6, CH_2_), 40.6 (C-6a, CH), 29.1 (C-1″, CH_2_), 25.2 (C-4″, CH_3_), 24.7 (C-4′, CH_3_), 24.4 (C-1′,CH_2_), 23.0 (C-5′, CH_3_), 20.6 (COCH_3_-9, CH_3_), 19.4 (COCH_3_-2′, CH_3_), 17.9 (C-5″, CH_3_); HR-(+)-ESIMS *m*/*z* 515.2054 [M + Na]^+^ (calcd 515.2051 for C_29_H_3_2O_7_Na).

Compound **3a**: ^1^H NMR (C_6_D_6_), δ 7.27 (1H, d, overlapped, H-1), 6.77 (1H, d, *J* = 8.5 Hz, H-2), 6.72 (1H, s, H-10), 6.68 (1H, s, H-7), 5.62 (1H, app t, *J* = 7.3 Hz, H-2″), 5.11 (1H, app t, *J* = 4.8 Hz, H-2′), 5.10 (1H, d, *J* = 7.0 Hz, H-11a), 4.94 (1H, s, H-4″a), 4.79 (1H, s, H-4″b), 3.95 (1H, dd, *J* = 11.0, 5.4 Hz, H_2_-6α), 3.10 (1H, dd, *J* = 11.0, 11.0 Hz, H_2_-6β), 3.01 (1H, dd, *J* = 17.7, 5.1 Hz, H_2_-1′α), 2.99 (1H, overlapped, H-6a), 2.95 (1H, overlapped, H_2_-1′β), 2.93 (1H, overlapped, H_2_-1″b), 2.71 (1H, dd, *J* = 15.7, 7.4 Hz, H_2_-1″a), 1.90 (3H, s, COCH_3_-9), 1.66 (3H, s, H-5″), 1.66 (3H, s, COCH_3_-2″), 1.58 (3H, s, (3H, s, COCH_3_-2′), 1.24 (3H, s, H-5′), 1.04 (3H, s, H-4′); ^13^C NMR (C_6_D_6_), δ 169.6 (COCH_3_-2′, C), 169.0 (COCH_3_-2″, C), 168.5 (COCH_3_-9, C), 159.6 (C-10a, C), 153.9 (C-4a, C), 151.1 (C-9, C9), 142.4 (C-3″, C), 128.2 (C-1, CH), n.d (C-6b, C), 125.9 (C-7, CH), 113.1 (C-2″, CH_2_), n.d. (C-11b, C), 110.9 (C-2, CH), 107.4 (C-4, C), 104.5 (C-10, CH), 78.7 (C-11a, CH), 77.0 (C-2″, CH), 75.0 (C-3′, C), 70.1 (C-2′, CH), 66.0 (C-6, CH_2_), 39.2 (C-6a, CH), 34.0 (C-1″, CH_2_), 24.1 (C-4′, CH_3_), 23.9 (C-1′, CH_2_), 22.3 (C-5′, CH_3_), 20.6 (COCH_3_-2′, CH_3_), 20.1 (COCH_3_-9, CH_3_), 19.5 (COCH_3_-2″, CH_3_). 16.4 (C-5″, CH_3_); HR-(+)-ESIMS *m*/*z* 573.2087 [M + Na]^+^ (calcd 573.2106 for C_31_H_34_O_9_Na).

### 4.5. Preparation of MTPA Esters of ***1***

To a CH_2_Cl_2_ solution (0.5 mL) of bituminarin A (**1**, 0.2 mg) a catalytic amount of DMPA and (*R*)-MTPA-Cl (3.5 μL) were added, and the mixture was stirred overnight at r.t. The reaction mixture was evaporated under vacuum to give a residue, which was purified by a pipette Pasteur silica gel column eluting with PE/DE (from 9:1 to 7:3) to give the corresponding (*S*)-MTPA ester (**1b**, 0.1 mg) of **1**. Similarly, the (*R*)-MTPA ester (**1c**, 0.1 mg) of **1** was prepared following the same procedure using as reagent (*S*)-MTPA-Cl.

(*S*)-MTPA-ester (**1b**): selected ^1^H NMR values (CD_3_COCD_3_), δ 7.29 (1H, d, *J* = 8.5 Hz, H-1), 6.51 (1H, d, *J* = 8.5 Hz, H-2), 5.23 (1H, app t, *J* = ~5.6 Hz, H-2′), 3.06 (1H, dd, *J* = 16.0, 7.5 Hz, H_2_-1′a), 2.92 (1H, dd, *J* = 16.0, 7.0 Hz, H_2_-1′b), 1.24 (3H, s, H-5′), 1.28 (3H, s, H-4′).

(*R*)-MTPA-ester (**1c**): selected ^1^H NMR values (CD_3_COCD_3_), δ 7.21 (1H, d, *J* = 8.5 Hz, H-1), 6.46 (1H, d, *J* = 8.5 Hz, H-2), 5.24 (1H, app t, *J* = ~5.6 Hz, H-2′), 3.05 (1H, dd, *J* = 16.0, 7.5 Hz, H_2_-1′a), 2.78 (1H, dd, *J* = 16.0, 7.0 Hz, H_2_-1′b), 1.34 (3H, s, H-5′), 1.34 (3H, s, H-4′).

(*S*)-MTPA-ester (**1b**): selected ^1^H NMR values (C_6_D_6_), δ 7.25 (1H, d, *J* = 8.5 Hz, H-1), 6.69 (1H, d, *J* = 8.5 Hz, H-2), 5.02 (1H, app t, *J* = ~5.6 Hz, H-2′), 3.20 (1H, dd, *J* = 16.0, 7.5 Hz, H_2_-1′a), 3.15 (1H, dd, *J* = 16.0, 7.0 Hz, H_2_-1′b), 1.05 (3H, s, H-5′), 0.93 (3H, s, H-4′).

(*R*)-MTPA-ester (**1c**): selected ^1^H NMR values (C_6_D_6_), δ 7.22 (1H, d, *J* = 8.5 Hz, H-1), 6.68 (1H, d, *J* = 8.5 Hz, H-2), 5.04 (1H, app t, *J* = ~5.6 Hz, H-2′), 2.83 (1H, dd, *J* = 16.0, 7.5 Hz, H-1′a), 3.01 (1H, dd, *J* = 16.0, 7.0 Hz, H-1′b), 1.15 (3H, s, H-5′), 1.01 (3H, s, H-4′).

### 4.6. Biological Activity

#### 4.6.1. Cell Cultures

The SH-SY5Y human neuroblastoma cell line (CRL-2266; American Type Culture Collection, Manassas, VA, USA) was cultured in Dulbecco minimum essential medium (DMEM) supplemented with 20% fetal bovine serum (FBS), 1% antibiotic–antimycotic mixture, 2 mmol/L L-glutamine in 5% CO_2_ humified incubator at 37 °C (all reagents from GIBCO, Invitrogen, Carlsbad, CA, USA). The cell medium was changed every 2–3 days. Cells were split and then plated after reaching 80% confluency, with densities from 4500 to 6000 cells/cm^2^ at each passage.

#### 4.6.2. Immunofluorescence

SH-SY5Y cells were fixed using 4% paraformaldehyde in 0.1 M phosphate buffer (PB), pH 7.4. Following fixation, samples were permeabilized 10 min with PB 0.3% Triton X 100 (Sigma-Aldrich, St. Louis, MI, USA) and finally incubated overnight with the primary antibodies Anti-Cytochrome C (1:100, mouse monoclonal Sc-13561 Santa Cruz Biotechnology, Paso Robles, CA, USA), anti-Caspase 3 (1:200, rabbit polyclonal AbCam, Cambridge, UK), and anti-Caspase 7 (1:100, Sc-56063, mouse monoclonal Santa Cruz Biotechnology, Paso Robles, CA, USA). After washing, the samples were then incubated for 2 h with a mixture of appropriate secondary antibodies Alexa Fluor 488 or 546 donkey anti-mouse or donkey anti-rabbit (Invitrogen) and the nuclei stained with DAPI (1 mg/mL; Sigma-Aldrich). Finally, the cells were mounted with an aqueous mounting medium (Aquatex, Merck, Darmstadt, Germany). Fluorescence-labeled cells were analyzed with a Leica DMI6000 fluorescence microscope equipped with an x-y-z motorized stage and cooled digital camera Leica K5 (Leica Microsystems, Buccinasco, Milan, Italy). Images were digitally acquired at the same magnification and processed for quantification of fluorescence by Leica LAS X software (Leica Application Suite X 3.7.4.23463). The optimal focus was chosen for each fluorescence channel of the z-stacked images and merged to obtain the best multichannel image for each well analyzed to measure the percentage of immunolabeled area/well for each immunostaining. All the measurements were performed in quadruplicate. Statistical and quantification analyses were conducted by an observer blinded to the experimental design using Fiji, an image-processing package of ImageJ software (version 2.14.0) developed by the National Institutes of Health, USA. The LIVE/DEAD^®^ Viability/Cytotoxicity two-color assay (Invitrogen) was applied to the cells before and after erybraedin C 1 µg/mL treatment to determine the percentage of cell viability by fluorescence microscopy.

#### 4.6.3. Incucyte Live Cell Scanning

To observe the temporal development of the oxidative stress, and mitochondrial morphology of SH-SY5Y cells in response to bitucarpin A and erybraedin C treatment, a live action imaging assay was performed using the Zoom Incucyte scanner (Sartorius). Human neuroblastoma SH-SY5Y cells were plated in 24× multi wells with a density of 10,000/15,000 cells per well. The multi wells were inserted into the scanner placed in the incubator in order to preserve the ideal environmental conditions and were scanned at progressive time points by covering a framework of 48 h. Each well was scanned by keeping images from 16 different areas/well; that is the maximum number of images acquired for the best proxy-live representation for wells. Each well was scanned every 30 min; that is the time required for cooling the mechanical components of the scanner. The image acquisition was performed with 20× magnification objective in phase contrast and red fluorescence. All images were then analyzed using the integrated Zoom Incucyte software (version 20181.1.6628.28170) after appropriate calibration of analysis. Quantitative analysis provided the time-based trend of the percentage of cells labelled with CellRox or Mitotracker which were identified in the scanned areas for each well within the time lapse scanning.

The SH-SY5Y cells were seeded 4000 cells × well in 24 wells plates to be tested by growth inhibition assay, identifying the IC_50_ value of growth inhibition for bitucarpin A and erybraedin C.

#### 4.6.4. Reagents

The carbocyanine-based MitoTracker™ Deep and the Red CellROX™ Deep Red were exploited as live-cell fluorescent probes having the advantages of not interfering with cellular vitality during the analysis of the cell responses to the oxidative stress. The carbocyanine-based MitoTracker™ Deep Red (Thermo Fisher Scientific Inc. Waltham, MA, USA) has active mitochondrial stains in live cells with an absorption/emission maxima of 644/665 nm. It contains a mildly thiol-reactive chloromethyl portion so that mitochondrial staining is retained even if the mitochondrial membrane potential is lost. The CellROX™ Deep Red Reagent (Thermo Fisher Scientific Inc., Waltham, MA, USA) is a fluorogenic probe for measuring cellular oxidative stress, in both live and fixed cell imaging, with absorption/emission maxima at ~644/665 nm. The cell-permeant dye is non-fluorescent while in a reduced state and exhibits bright fluorescence upon oxidation by reactive oxygen species (ROS). MitoTracker™ Deep Red was used at a working concentration of 200 nM while CellROX™ Deep Red Reagent was applied at a working concentration of 2.5 µM. Bitucarpin A and erybraedin C were evaluated at two concentrations of 0.1 and 1 µg/mL. The LIVE/DEAD^®^ Viability/Cytotoxicity two-color assay (Invitrogen) was used according to the Invitrogen recommended protocol.

#### 4.6.5. Statistical Analysis

Statistical analysis was performed using Graphpad prism 10. All the grouped datasets were evaluated performing a two-way ANOVA test with a Sidak or Bonferroni post-hoc test. All the data were assessed as significative with a *p* value ≤ 0.05.

## Figures and Tables

**Table 1:** ^1^H and ^13^C NMR data ^a^ for bituminarins A–C (**1–3**) in CD_3_COCD_3_.

	1		2		3	
n.	δ*_C_*, Type	δ*_H_* (*J* in Hz)	δ*_C_*, Type	δ*_H_* (*J* in Hz)	δ*_C_*, Type	δ*_H_* (*J* in Hz)
1	130.2, CH	7.19, d (8.5)	130.2, CH	7.19, d (8.6)	130.2, CH	7.20, d (8.4)
2	111.1, CH	6.44, d (8.5)	111.1, CH	6.44, d (8.6)	111.2, CH	6.45, d (8.4)
3	155.9 *, C		154.2 *, C		155.3 *, C	
4	108.9 *, C		108.8 *, C		109.0 *, C	
4a	154.5 *, C		154.4 *, C		155.2 *, C	
6	67.5, CH_2_	4.31α, dd (10.5, 4.6) 3.57β, dd (10.5, 10.5)	67.4, CH_2_	4.31 α, dd (10.6, 4.8) 3.60 β, dd (10.6,10.6)	67.4, CH_2_	4.30 α, dd (10.9, 4.8) 3.62 α, dd (10.9,10.9)
6a	40.5, CH	3.52, m	40.6, CH	3.51, m	40.5, CH	3.53, ddd (10.1, 7.0, 4.8)
6b	119.2 *, C		118.8 *, C		119.1 *, C	
7	126.0, CH	7.03, s	126.0, CH	7.03, s	127.9, CH	7.06, s
8	120.7 *, C		120.1 *, C		120.0 *, C	
9	155.9 *, C		156.0 *, C		158.5 *, C	
10	98.2, CH	6.31, s	98.3, CH	6.31, s	99.1, CH	6.29, s
10a	163.7 *, C		159.5 *, C		160.2 *, C	
11a	79.3, CH	5.42, d (6.9)	79.3, CH	5.43, d (7.0)	79.4, CH	5.45, d (7.0)
11b	113.5 *, C		112.7, C		112.8 *, C	
1′	27.1, CH_2_	2.88α, dd (16.9, 5.5) 2.48β, dd (16.9, 7.9)	27.1, CH_2_	2.86α, dd (17.0, 5.4) 2.52β, dd (17.0, 7.4)	27.1, CH_2_	2.87α, dd (17.3, 5.6) 2.53β, dd (17.3, 7.4)
2′	69.6, CH	3.74, m	69.5, CH	3.75, m	69.5, CH	3.75, m
3′	78.8 *, C		77.8 *, C		79.3 *, C	
4′	26.0, CH_3_	1.31α, s	25.8, CH_3_	1.31α, s	25.8, CH_3_	1.31 α, s
5′	20.3, CH_3_	1.21β, s	20.8, CH_3_	1.21β, s	20.8, CH_3_	1.21β, s
1″	28.7, CH_2_	3.27, dd (15.0, 7.2) 3.21, dd (15.0, 7.6)	28.7, CH_2_	3.27, dd (15.8, 7.2) 3.21, dd (15.8, 7.4)	38.6, CH_2_	2.78 (overlapped)
2″	124.5, CH	5.31, br t (7.0)	124.4, CH	5.31, br t (7.0)	77.4, CH	4.32, m
3″	131.7 *, C		131.9 *, C		148.6 *, C	
4″	25.9, CH_3_	1.70, s	25.9, CH_3_	1.71, s	110.4, CH_2_	4.94, s 4.77, s
5″	17.5, CH_3_	1.70, s	17.3, CH_3_	1.71, s	18.5, CH_3_	1.78, s

^a^ Assignments aided by COSY, ed-HSQC and HMBC (*J* = 7 Hz) experiments; * values deduced by HMBC.

## Data Availability

Data supporting reported results are available from authors.

## Supplementary Materials

The following supporting information can be downloaded at: https://www.mdpi.com/article/10.3390/molecules29153678/s1. Spectroscopic data (1D and 2D NMR, HRESIMS, ECD) for bituminarins A–C, acetyl bituminarins **1a**–**3a**, Mosher’s esters **1b**–**1c**, bitucarpin A and erybraedin C.

## References

[1] WFO Plant List. https://wfoplantlist.org/plant-list/.

[2] Ventura M.R., Mendez P., Flores M.P., Rodriguez R., Castanon J.I.R. (2000). Energy and protein content of *Tedera* (*Biturninaria bituminosa*). Cha. Opt. Med..

[3] Ventura M.R., Flores M.P., Castanon J.I.R. (1999). Nutritive value of forage shrubs: *Bituminaria bituminosa*, *Acacia salicina* and *Medicago arborea*. Cah. Options Méditerranéennes.

[4] Pistelli L., Noccioli C., Appendino G., Bianchi F., Sterner O., Ballero M. (2003). Pterocarpans from *Bituminaria morisiana* and *Bituminaria bituminosa*. Phytochemistry.

[5] Pecetti L., Tava A., Pagnotta M.A., Russi L. (2007). Variation in forage quality and chemical composition among Italian accessions of *Bituminaria bituminosa* (L.) Stirt. J. Sci. Food Agric..

[6] Pecetti L., Mella M., Tava A. (2016). Variation in Herbage Biochemical Composition among Pitch Trefoil (*Bituminaria bituminosa*) Populations from Elba Island, Italy. J. Agric. Food Chem..

[7] Koul B., Taak P., Kumar A., Kumar A., Sanyal I. (2019). Genus Psoralea: A review of the traditional and modern uses, phytochemistry and pharmacology. J. Ethnopharm..

[8] Tava A., Pecetti L., Ricci M., Pagnotta M.A., Russi L. (2007). Volatile compounds from leaves and flowers of *Bituminaria bituminosa* (L.) Stirt. (Fabaceae) from Italy. Flav. Fragr. J..

[9] Innocenti G., Piovan A., Filippini R., Caniato R., Cappelletti E.M. (1997). Quantitative recovery of furanocoumarins from *Psoralea bituminosa*. Phytochem. Anal..

[10] Azzouzi S., Zaabat N., Medjroubi K., Akkal S., Benlabed K., Smati F., Dijoux-Franca M.G. (2014). Phytochemical and biological activities of *Bituminaria bituminosa* L. (Fabaceae). Asian Pac. J. Trop. Med..

[11] Llorent-Martinez E.J., Spinola V., Gouveia S., Castilho P.P. (2015). HPLC-ESI-MS^n^ characterization of phenolic compounds, terpenoid saponins, and other minor compounds in *Bituminaria bituminosa*. Ind. Crop Prod..

[12] Selvam C., Jordan B.C., Prakash S., Mutisya D., Thilagavathi R. (2017). Pterocarpan scaffold: A natural lead molecule with diverse pharmacological properties. Eur. J. Med. Chem..

[13] Al-Maharik N. (2019). Isolation of naturally occurring novel isoflavonoids: An update. Nat. Prod. Rep..

[14] Veitch N.C. (2013). Isoflavonoids of the Leguminosae. Nat. Prod. Rep..

[15] Smadi A., Bitam F., Ciavatta M.L., Carbone M., Bertella A., Gavagnin M. (2020). Chemical constituents of the aerial parts of Algerian *Galium brunneum*: Isolation of new hydroperoxy sterol glucosyl derivatives. Phytochem. Lett..

[16] Kebbi S., Ciavatta M.L., Mahmoud A.M., Carbone M., Ligresti A., Seghiri R., Gavagnin M. (2021). Sesquiterpene lactones with the 12,8-guaianolide skeleton from Algerian *Centaurea omphalotricha*. Biomolecules.

[17] Bensaid S.O., Carbone M., Palomba L., Bicha S., Bentamene A., Carannante A., Gavagnin M., Ciavatta M.L. (2022). First occurrence of megastigmane glucosides in a plant of *Retama* genus. Chem. Biodivers..

[18] Mitscher L.A., Okwute S.K., Gollapudi S.R., Drake S., Avona E. (1988). Antimicrobial pterocarpans of Nigerian *Erythrina mildbraedii*. Phytochemistry.

[19] Nkengfack A.E., Vardamides J.C., Fomum Z.T., Meyers M. (1995). Prenylated isoflavanone from *Erythrina eriotricha*. Phytochemistry.

[20] Mitscher L.A., Okwute S.K., Gollapudi S.R., Keshavarz-Shokri A. (1988). Antimicrobial agents from higher plants. The isolation and structural characterization of two additional pterocarpan antimicrobial agents from Nigerian *Erythrina mildbraedii*. Heterocycles.

[21] Jois H.S., Manjunath B.L., Rao S.V. (1933). Chemische untersuchung der samen von *Psoralea corylifolia*, Linn. I. Chem. Zentralblatt..

[22] Jois H.S., Manjunath B.L., Rao S.V. (1933). Chemical examination of seeds of *Psoralea coryfolia*. J. Ind. Chem. Soc..

[23] Xiao G., Li G., Chen L., Zhang Z., Yin J.J., Wu T., Cheng Z., Wei X., Wang Z. (2010). Isolation of antioxidants from *Psoralea corylifolia* fruits using high-speed counter-current chromatography guided by thin layer chromatography-antioxidant autographic assay. J. Chromat. A.

[24] Späth E., Pesta O. (1934). Die constitution des angelicin. Ber. Dtsch. Chem. Ges..

[25] Schmitt A., Telikepalli H., Mitscher L.A. (1991). Plicatin B, the antimicrobial principle of *Psoralea juncea*. Phytochemistry.

[26] Eventi A., Lubrano V., Scarpato R., Turchi G. (2009). Protective effects of plicatin B on micronucleus induction in cultured human lymphocytes by different mutageno. Food Chem. Toxicol..

[27] Cottiglia F., Casu L., Bonsignore L., Casu M., Floris C., Leonti M., Gertsch J., Heilmann J. (2005). New cytotoxic prenylated isoflavonoids from *Bituminaria morisiana*. Planta Med..

[28] Tesauro C., Fiorani P., D’Annessa I., Chillemi G., Turchi G., Desideri A. (2010). Erybraedin C, a natural compound from the plant *Bituminaria bituminosa*, inhibits both the cleavage and religation activities of human topoisomerase I. Biochem. J..

[29] Sakurai Y., Sakurai N., Taniguchi M., Naganishi Y., Bastow K.F., Wang X., Cragg G.M., Lee K.-H. (2006). Rautandiols A and B, pterocarpans and cytotoxic constituents from *Neorautanenia mitis*. J. Nat. Prod..

[30] Ren F.-C., Wang L.-X., Lv Y.-F., Hu J.-M., Zhou J. (2021). Structure revision of four classes of prenylated aromatic natural products based on a rule for diagnostic 13C NMR chemical shifts. J. Org. Chem..

[31] Goel A., Kumar A., Raghuvanshi A. (2013). Synthesis, Stereochemistry, Structural Classification, and Chemical Reactivity of Natural Pterocarpans. Chem. Rev..

[32] Tarbeeva D.V., Pislyagin E.A., Menchinskaya E.S., Berdyshev D.V., Kalinovskiy A.I., Grigorchuk V.P., Mishchenko N.P., Aminin D.L., Fedoreyev S.A. (2022). Polyphenolic compounds from *Lespedeza bicolor* protect neuronal cells from oxidative stress. Antioxidants.

[33] Haga N., Fujita N., Tsuruo T. (2003). Mitochondrial aggregation precedes cytochrome c release from mitochondria during apoptosis. Oncogene.

[34] Zamzami N., Marchetti P., Castedo M., Zanin C., Vayssiere J.L., Petit P.X., Kroemer G. (1995). Reduction in mitochondrial potential constitutes an early irreversible step of programmed lymphocyte death in vivo. J. Exp. Med..

[35] Bock F.J., Tait S.W.J. (2020). Mitochondria as multifaceted regulators of cell death. Nat. Rev. Mol. Cell Biol..

[36] Karbowski M., Youle R. (2003). Dynamics of mitochondrial morphology in healthy cells and during apoptosis. Cell Death Differ..

[37] Gilkerson R.W., Margineantu D.H., Capaldi R.A., Selker J.M.L. (2000). Mitochondrial DNA depletion causes morphological changes in the mitochondrial reticulum of cultures human cells. FEBS Lett..

[38] Westrate L.M., Drocco J.A., Martin K.R., Hlavacek W.S., MacKeigan J.P. (2014). Mitochondrial morphological features are associated with fission and fusion events. PLoS ONE.

[39] Ngo J., Choi D.W., Stanley I.A., Stiles L., Molina A.J.A., Chen P.H., Lako A., Sung I.C.H., Goswami R., Kim M.Y. (2023). Mitochondrial morphology controls fatty acid utilization by changing CPT1 sensitivity to malonyl-CoA. EMBO J..

[40] Nassief S.M., Amer M.E., Shawky E., Sishtla K., Mas-Claret E., Muniyandi A., Corson T.W., Mulholland D.A., Sawsan El-Masry S. (2023). Antiangiogenic pterocarpan and flavonoid constituents of *Erythrina lysistemon*. J. Nat. Prod..

[41] D’Angiolillo F., Pistelli L., Noccioli C., Ruffoni B., Piaggi S., Scarpato R., Pistelli L. (2014). In vitro Cultures of *Bituminaria bituminosa*: Pterocarpan, furanocoumarin and isoflavone production and cytotoxic activity evaluation. Nat. Prod. Commun..

[42] Dewick M., Harborne J.B. (1988). The Flavonoids: Advances in Research.

[43] Wu J., Gu L., Geacintov N.E., Li G.-M. (1999). Mismatch repair processing of carcinogen-DNA adducts triggers apoptosis. Mol. Cell. Biol..

[44] Kaufmann S.H. (1998). Cell death induced by topoisomerase-targeted drugs—More questions than answers. Biochem. Biophys. Acta.

[45] Fofana S., Ouédraogo M., Esposito R.C., Ouedraogo W.P., Delporte C., Van Antwerpen P., Mathieu V., Guissou I.P. (2021). Systematic review of potential anticancerous activities of *Erythrina senegalensis* DC (Fabaceae). Plants.

