# Supplementary material for: Prenyl Pterocarpans from Algerian Bituminaria bituminosa and Their Effects on Neuroblastoma
Source: Molecules. 2024 Aug 2;29(15):3678. doi: 10.3390/molecules29153678 (PMC11313871; doi:10.3390/molecules29153678)
Supplement: Supplementary file 1 [file molecules-29-03678-s001.zip › molecules-3103194-supplementary.pdf]

Supplementary Material

Prenyl pterocarpans from aerial parts of Algerian *Bituminaria bituminosa* and their effects on neuroblastoma

Hakim Benhabrou,[1] Fatma Bitam,[2] Luigia Cristino,[3]* Alessandro Nicois,[3,4] Marianna Carbone,[3] Dibi Ammar,[1] Margherita Gavagnin,[3] and Maria Letizia Ciavatta[3]*

[1]Faculté des Sciences de la Matière, Département de Chimie, Laboratoire de Chimie et Chimie de l'Environnement (LCCE), Université de Batna 1, 05000, Batna, Algeria.

[2]Faculté de Médecine, Département de Pharmacie, Université de Batna 2, 05000, Batna, Algeria.

[3]Consiglio Nazionale delle Ricerche, Istituto di Chimica Biomolecolare, Via Campi Flegrei 34, 80078 Pozzuoli (Na), Italy.

[4]University of Urbino 'Carlo Bo', Department of Biomolecular Sciences, Via Santa Chiara, 27, 61029 Urbino, Italy.

***Corresponding authors**

Email: lciavatta@icb.cnr.it; Phone +39 081 867 52 43 Fax +39 081 804 17 70

Email: lcristino@icb.cnr.it; Phone +39 081 867 5

Figure S1. ^{1}H NMR spectrum of bituminarin A (**1**) (600 MHz, Acetone-*d*6)
Figure S2. COSY spectrum of bituminarin A (**1**) (600 MHz, Acetone-*d*6)
Figure S3. ed-HSQC spectrum of bituminarin A (**1**) (600 MHz, Acetone-*d*6)
Figure S4. HMBC spectrum of bituminarin A (**1**) (600 MHz, Acetone-*d*6)
Figure S5. NOESY spectrum of bituminarin A (**1**) (600 MHz, Acetone-*d*6)
Figure S6. ^{13}C NMR spectrum of bituminarin A (**1**) (150 MHz, Acetone-*d*6)
Figure S7. HR ESIMS spectrum of bituminarin A (**1**)
Figure S8. ECD curve of bituminarin A (**1**)
Figure S9. ^{1}H NMR spectrum of bituminarin B (**2**) (600 MHz, Acetone-*d*6)
Figure S10. COSY spectrum of bituminarin B (**2**) (600 MHz, Acetone-*d*6)
Figure S11. ed-HSQC spectrum of bituminarin B (**2**) (600 MHz, Acetone-*d*6)
Figure S12. HMBC spectrum of bituminarin B (**2**) (600 MHz, Acetone-*d*6)
Figure S13. NOESY spectrum of bituminarin B (**2**) (600 MHz, Acetone-*d*6)
Figure S14. ^{13}C NMR spectrum of bituminarin B (**2**) (150 MHz, Acetone-*d*6)
Figure S15. HR ESIMS spectrum of bituminarin B (**2**)
Figure S16. ECD curve of bituminarin B (**2**)
Figure S17. ^{1}H NMR spectrum of bituminarin C (**3**) (600 MHz, Acetone-*d*6)
Figure S18. COSY spectrum of bituminarin C (**3**) (600 MHz, Acetone-*d*6)
Figure S19. ed-HSQC spectrum of bituminarin C (**3**) (600 MHz, Acetone-*d*6)
Figure S20. HMBC spectrum of bituminarin C (**3**) (600 MHz, Acetone-*d*6)
Figure S21. ^{13}C NMR spectrum of bituminarin C (**3**) (150 MHz, Acetone-*d*6)
Figure S22. HR ESIMS spectrum of bituminarin C (**3**)
Figure S23. ECD curve of bituminarin C (**3**)
Figure S24. ^{1}H NMR spectrum of bituminarin A (**1**) (600 MHz, C_6D_6)
Figure S25. COSY spectrum of bituminarin A (**1**) (600 MHz, C_6D_6)
Figure S26. ed-HSQC spectrum of bituminarin A (**1**) (600 MHz, C_6D_6)
Figure S27. HMBC spectrum of bituminarin A (**1**) (600 MHz, C_6D_6)
Figure S28. ^{1}H NMR spectrum of bituminarin B (**2**) (600 MHz, C_6D_6)
Figure S29. COSY spectrum of bituminarin B (**2**) (600 MHz, C_6D_6)
Figure S30. ed-HSQC spectrum of bituminarin B (**2**) (600 MHz C_6D_6)
Figure S31. HMBC spectrum of bituminarin B (**2**) (600 MHz, C_6D_6)
Figure S32. ^{1}H NMR spectrum of bituminarin C (**3**) (600 MHz, C_6D_6)
Figure S33. COSY spectrum of bituminarin C (**3**) (400 MHz, C_6D_6)
Figure S34. ed-HSQC spectrum of bituminarin C (**3**) (400 MHz, C_6D_6)
Figure S35. HMBC spectrum of bituminarin C (**3**) (600 MHz, C_6D_6)
Figure S36. ^{1}H NMR spectrum of acetylated bituminarin A (**1a**) (600 MHz, C_6D_6)
Figure S37. NOESY spectrum of acetylated bituminarin A (**1a**) (600 MHz, C_6D_6)
Figure S38. ed-HSQC spectrum of acetylated bituminarin A (**1a**) (600 MHz, C_6D_6)
Figure S39. HMBC spectrum of acetylated bituminarin A (**1a**) (600 MHz, C_6D_6)
Figure S40. ^{1}H NMR spectrum of acetylated bituminarin B (**2a**) (600 MHz, C_6D_6)
Figure S41. NOESY spectrum of acetylated bituminarin B (**2a**) (600 MHz, C_6D_6)

Figure S42. ed-HSQC spectrum of acetylated bituminarin B (**2a**) (600 MHz C_6D_6)
Figure S43. HMBC spectrum of acetylated bituminarin B (**2a**) (600 MHz, C_6D_6)
Figure S44. ^{1}H NMR spectrum of acetylated bituminarin C (**3a**) (600 MHz, C_6D_6)
Figure S45. COSY spectrum of acetylated bituminarin C (**3a**) (400 MHz, C_6D_6)
Figure S46. ed-HSQC spectrum of acetylated bituminarin C (**3a**) (400 MHz, C_6D_6)
Figure S47. HMBC spectrum of acetylated bituminarin C (**3a**) (600 MHz, C_6D_6)
Figure S48. ^{1}H NMR spectrum of (*S*)-MTPA-ester of bituminarin A (**1b**) (600 MHz, Acetone-*d*6)
Figure S49. ed-HSQC spectrum of (*S*)-MTPA-ester of bituminarin A (**1b**) (600 MHz, Acetone-*d*6)
Figure S50. ^{1}H NMR spectrum of (*R*)-MTPA-ester of bituminarin A (**1c**) (600 MHz, Acetone-*d*6)
Figure S51. ed-HSQC spectrum of (*R*)-MTPA-ester of bituminarin A (**1c**) (600 MHz, Acetone-*d*6)
Figure S52. ^{1}H NMR spectrum of (*S*)-MTPA-ester of bituminarin A (**1b**) (600 MHz, C_6D_6)
Figure S53. ed-HSQC spectrum of (*S*)-MTPA-ester of bituminarin A (**1b**) (600 MHz, C_6D_6)
Figure S54. ^{1}H NMR spectrum of (*R*)-MTPA-ester of bituminarin A (**1c**) (600 MHz, C_6D_6)
Figure S55. ed-HSQC spectrum of (*R*)-MTPA-ester of bituminarin A (**1c**) (600 MHz, C_6D_6)
Figure S56.^{1}H NMR spectrum of bituminarin A (**1**) (400 MHz, $CDCl_3$)
Figure S57. COSY spectrum of bituminarin A (**1**) (400 MHz, $CDCl_3$)
*Figure S58.*ed-HSQC spectrum of bituminarin A (**1**) (400 MHz, $CDCl_3$)
Figure S59. HMBC spectrum of bituminarin A (**1**) (400 MHz, $CDCl_3$)
Figure S60. ^{1}H NMR spectrum of bituminarin B (**2**) (400 MHz, $CDCl_3$)
Figure S61. COSY spectrum of bituminarin B (**2**) (400 MHz, $CDCl_3$)
Figure S62. ed-HSQC spectrum of bituminarin B (**2**) (400 MHz, $CDCl_3$)
Figure S63. HMBC spectrum of bituminarin B (**2**) (400 MHz, $CDCl_3$)
Figure S64.^{1}H NMR spectrum of erybraedin C (**5**) (600 MHz, $CDCl_3$)
Figure S65.^{13}C NMR spectrum of erybraedin C (**5**) (150 MHz, $CDCl_3$)
*Figure S66.*COSY spectrum of erybraedin C (**5**) (600 MHz, $CDCl_3$)
*Figure S67.*ed-HSQC spectrum of erybraedin C (**5**) (600 MHz, $CDCl_3$)
Figure S68. HMBC spectrum of erybraedin C (**5**) (600 MHz, $CDCl_3$)
Figure S69. ^{1}H NMR spectrum of bitucarpin A (**4**) (400 MHz, $CDCl_3$)
Figure S70. COSY spectrum of bitucarpin A (**4**) (400 MHz, $CDCl_3$)
Figure S71. ed-HSQC spectrum of bitucarpin A (**4**) (400 MHz, $CDCl_3$)
Figure S72. HMBC spectrum of bitucarpin A (**4**) (400 MHz, $CDCl_3$)

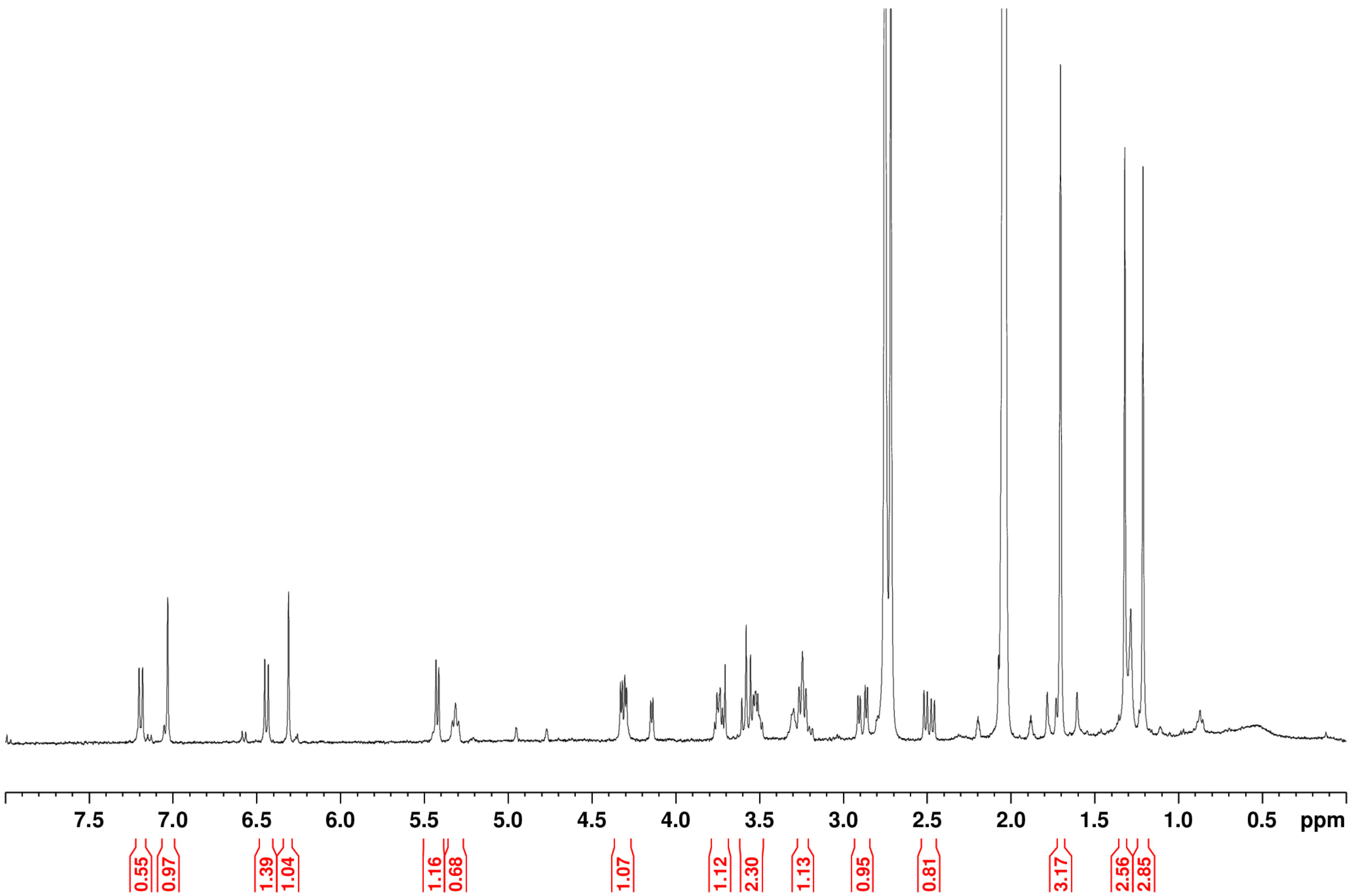

^{1}H NMR spectrum of bituminarin A (**1**) (600 MHz, Acetone-*d*6)

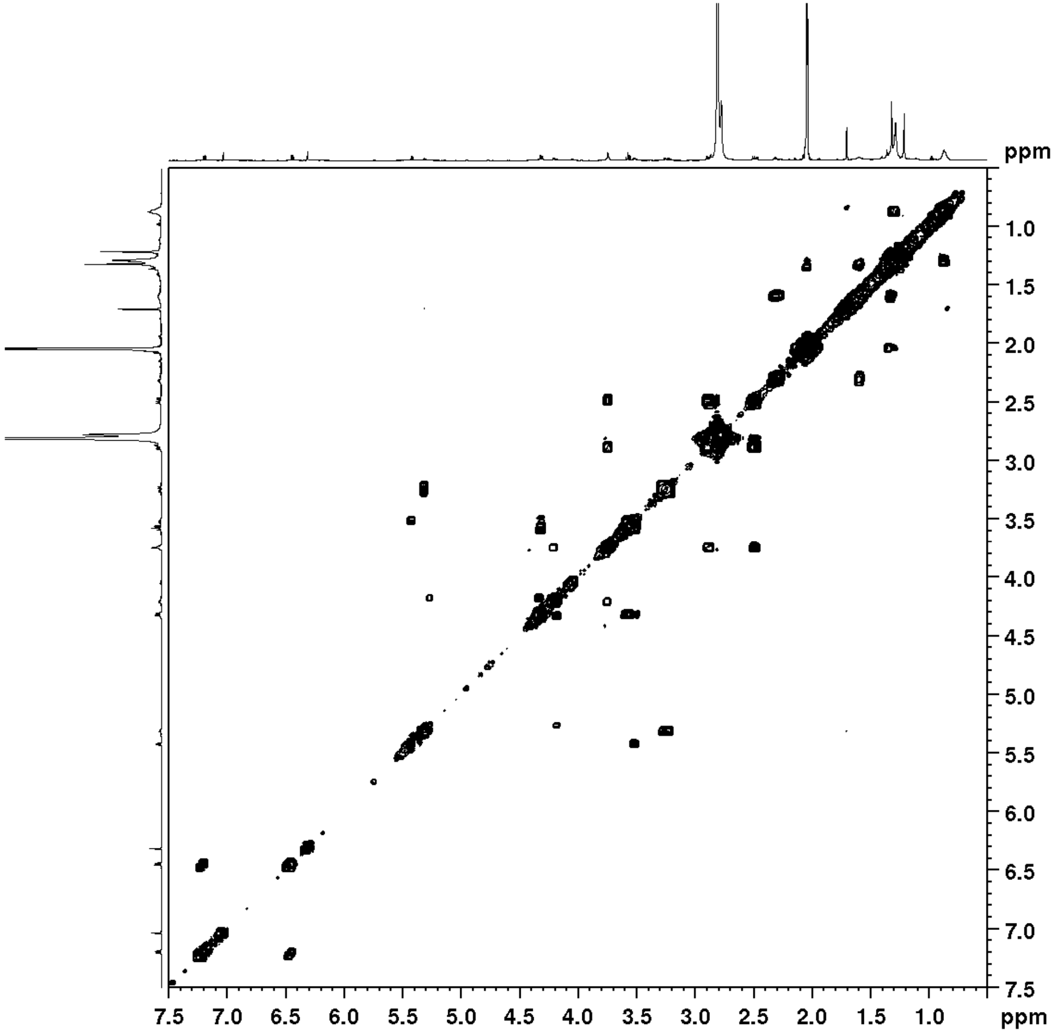

COSY spectrum of bituminarin A (**1**) (600 MHz, Acetone-*d*6)

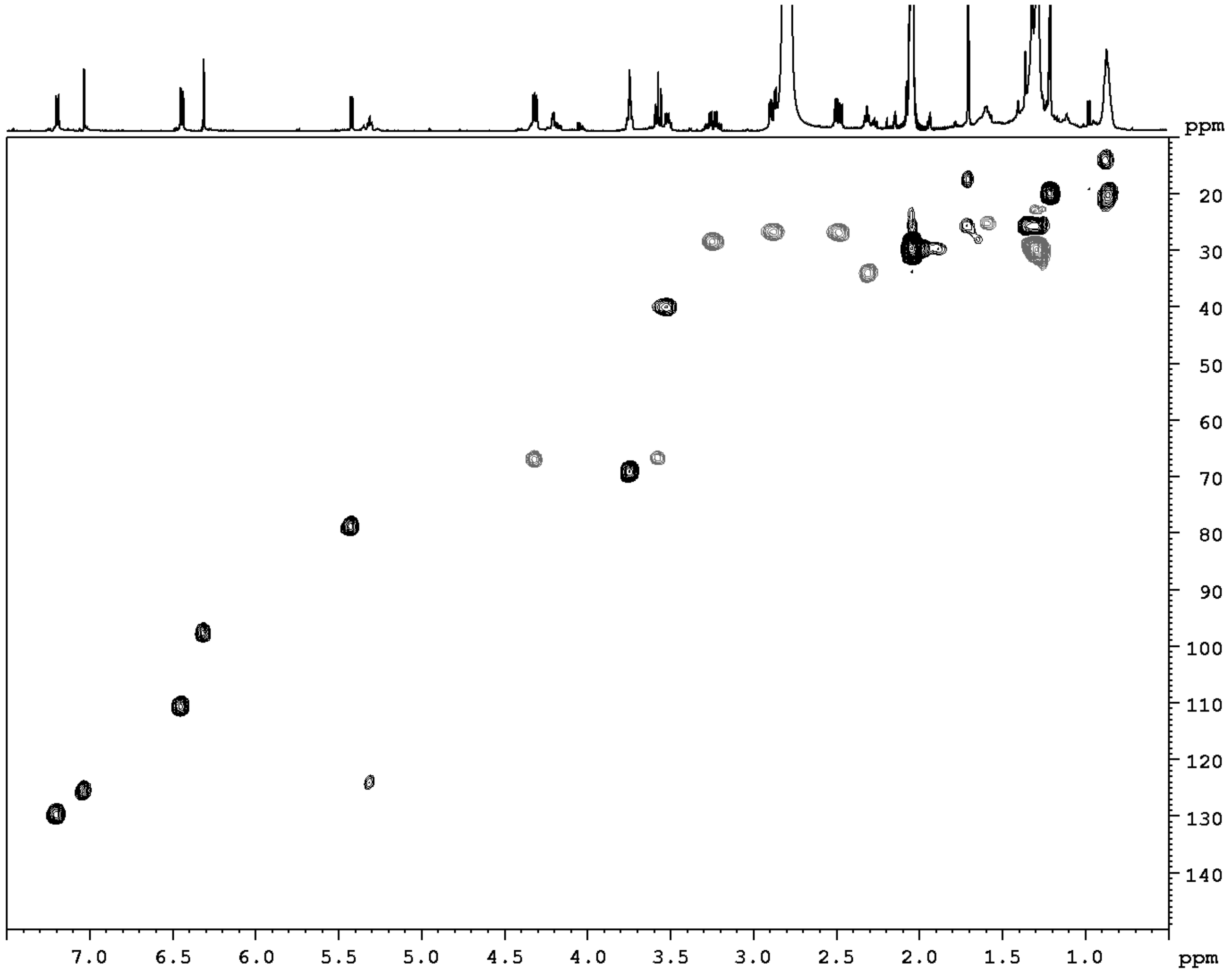

ed-HSQC spectrum of bituminarin A (**1**) (600 MHz, Acetone-*d*6)

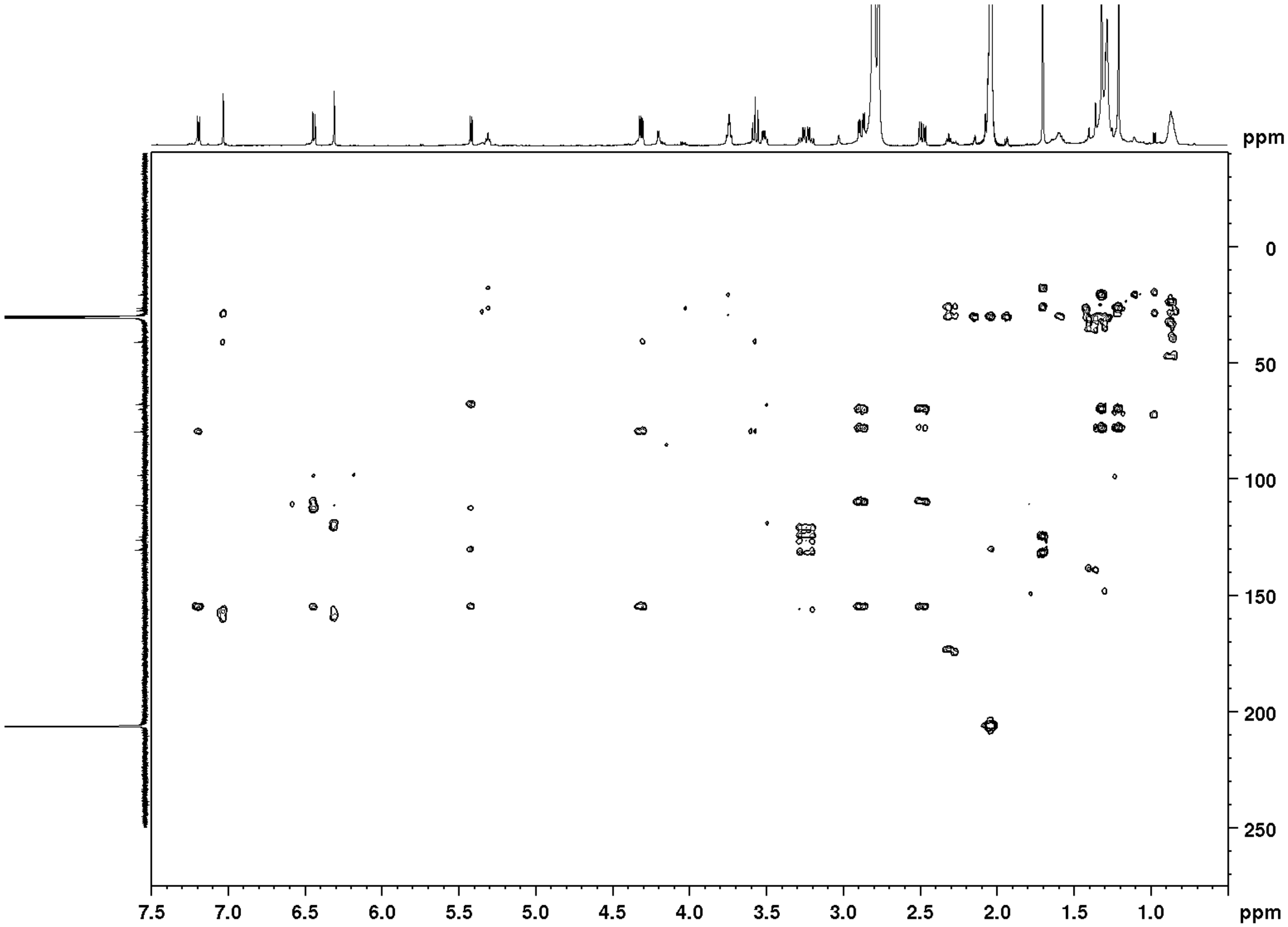

HMBC spectrum of bituminarin A (**1**) (600 MHz, Acetone-*d*6)

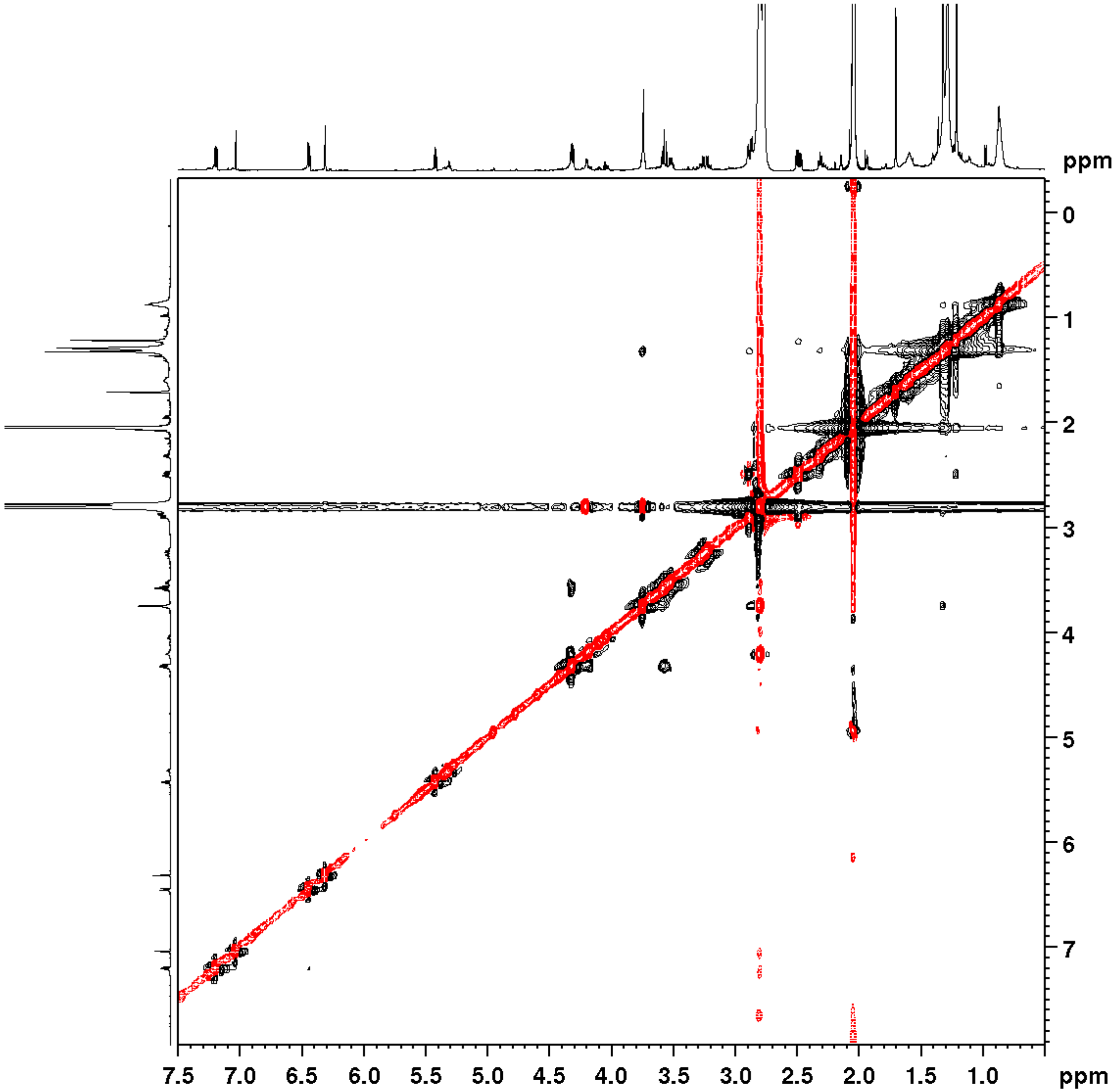

NOESY spectrum of bituminarin A (**1**) (600 MHz, Acetone-*d6*)

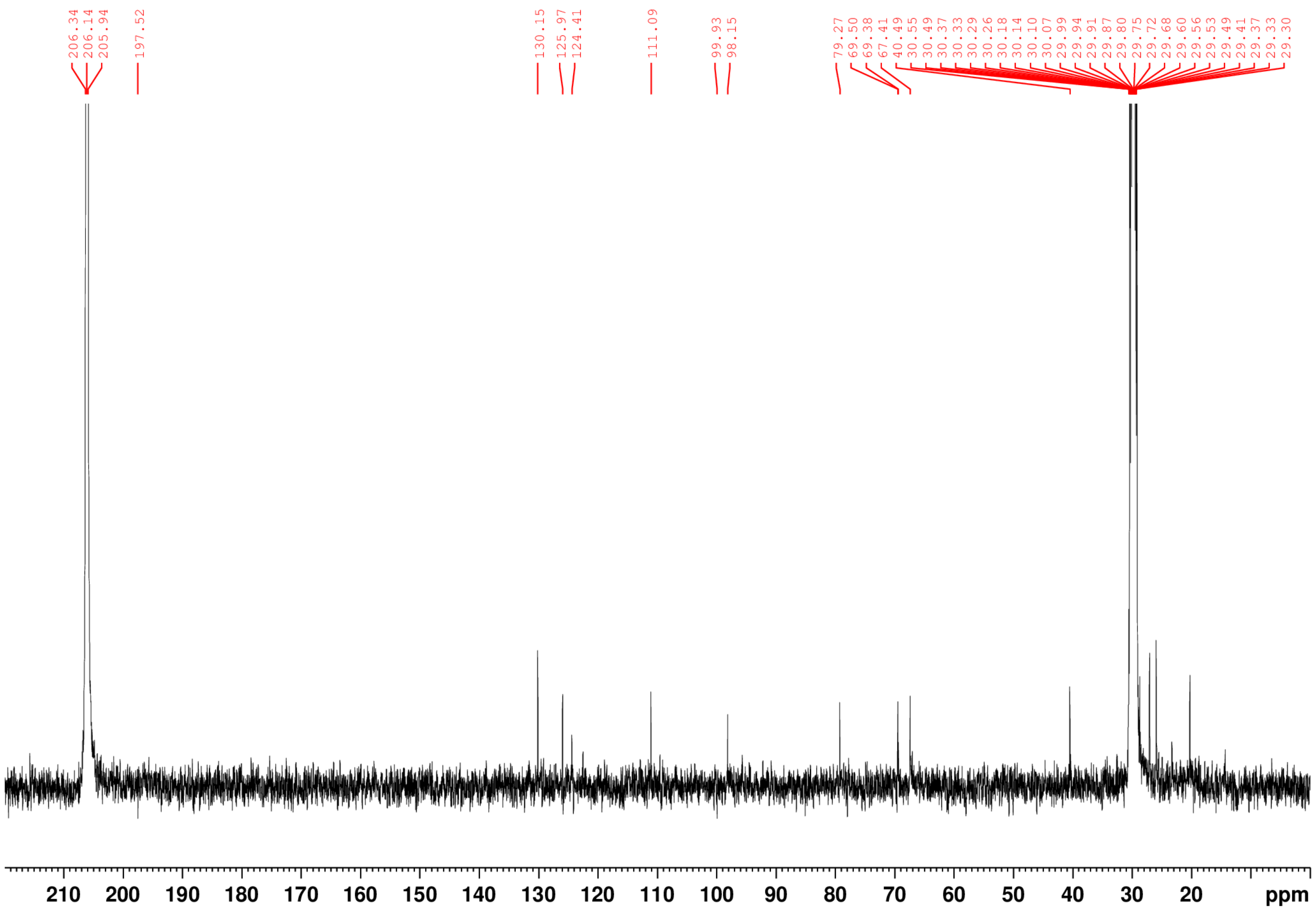

^{13}C NMR spectrum of bituminarin A (**1**) (150 MHz, Acetone-*d*6)

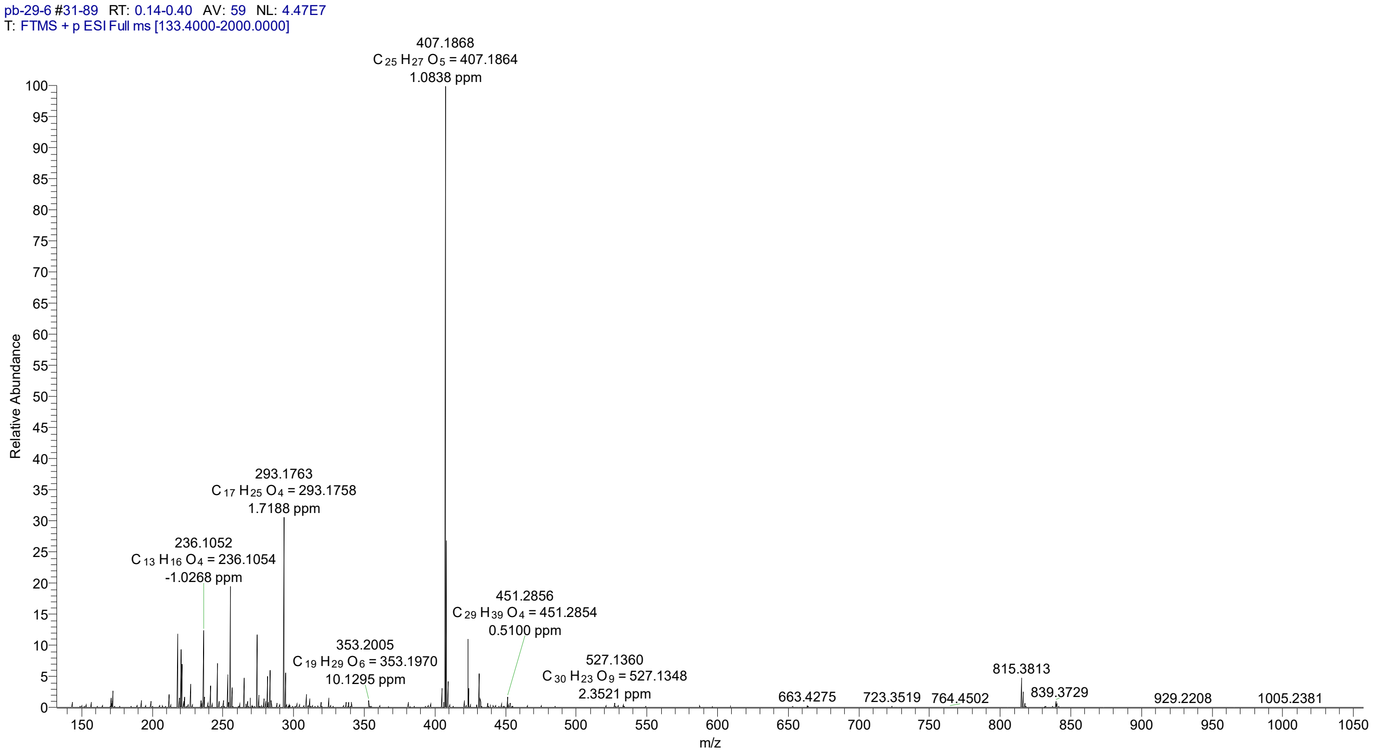

HR ESIMS spectrum of bituminarin A (**1**)

ECD curve of bituminarin A (**1**) (CH_3OH)

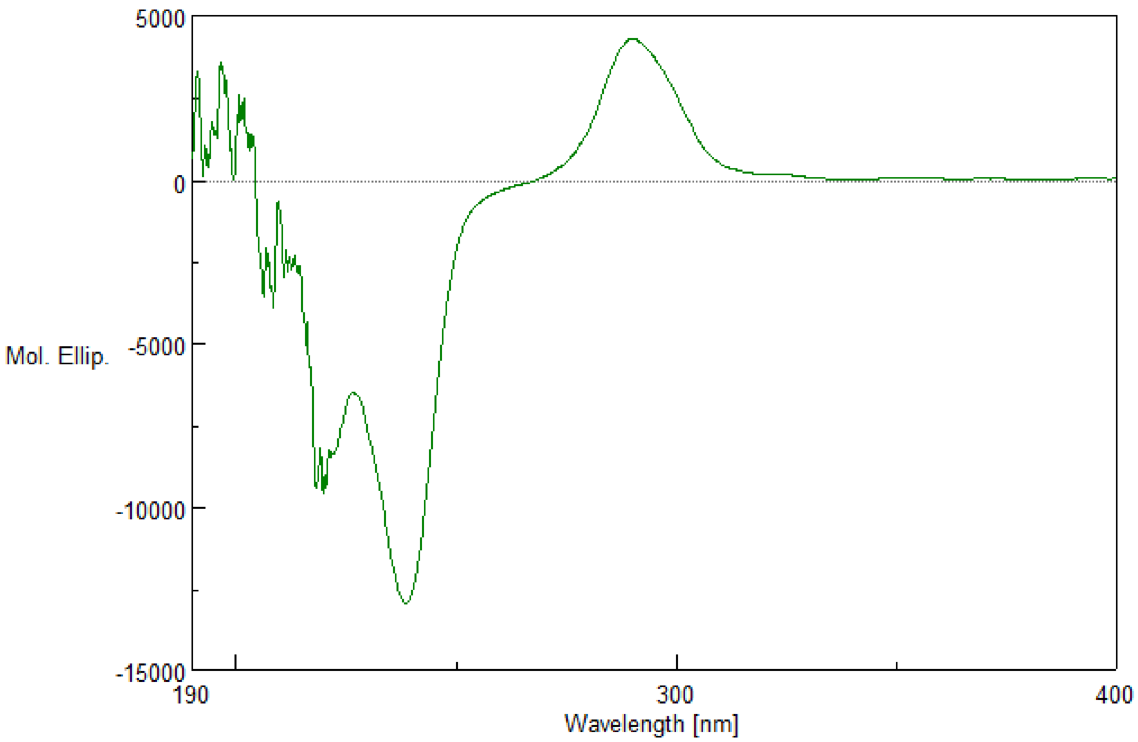

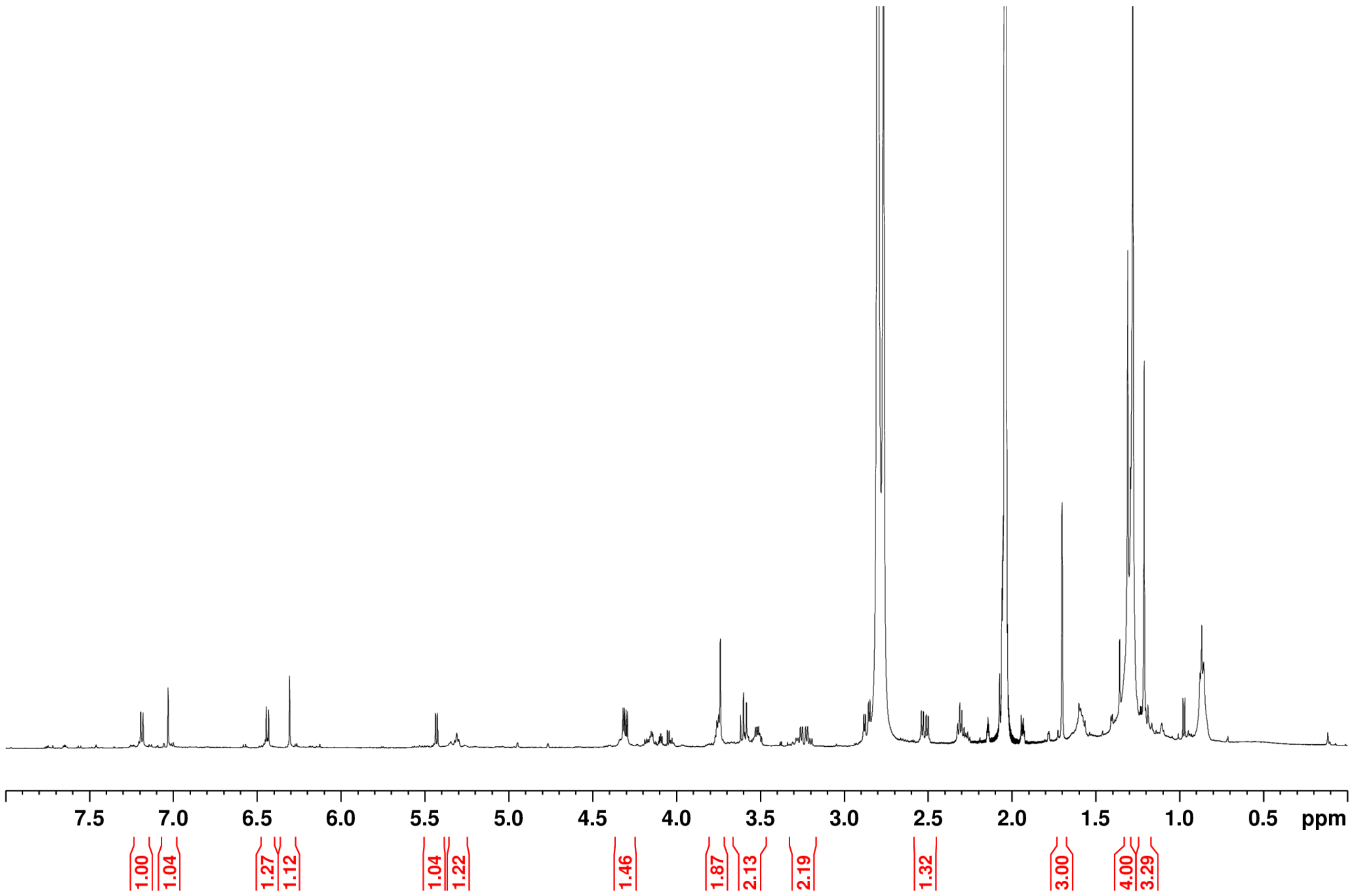

^{1}H NMR spectrum of bituminarin B (**2**) (600 MHz, Acetone-*d*6)

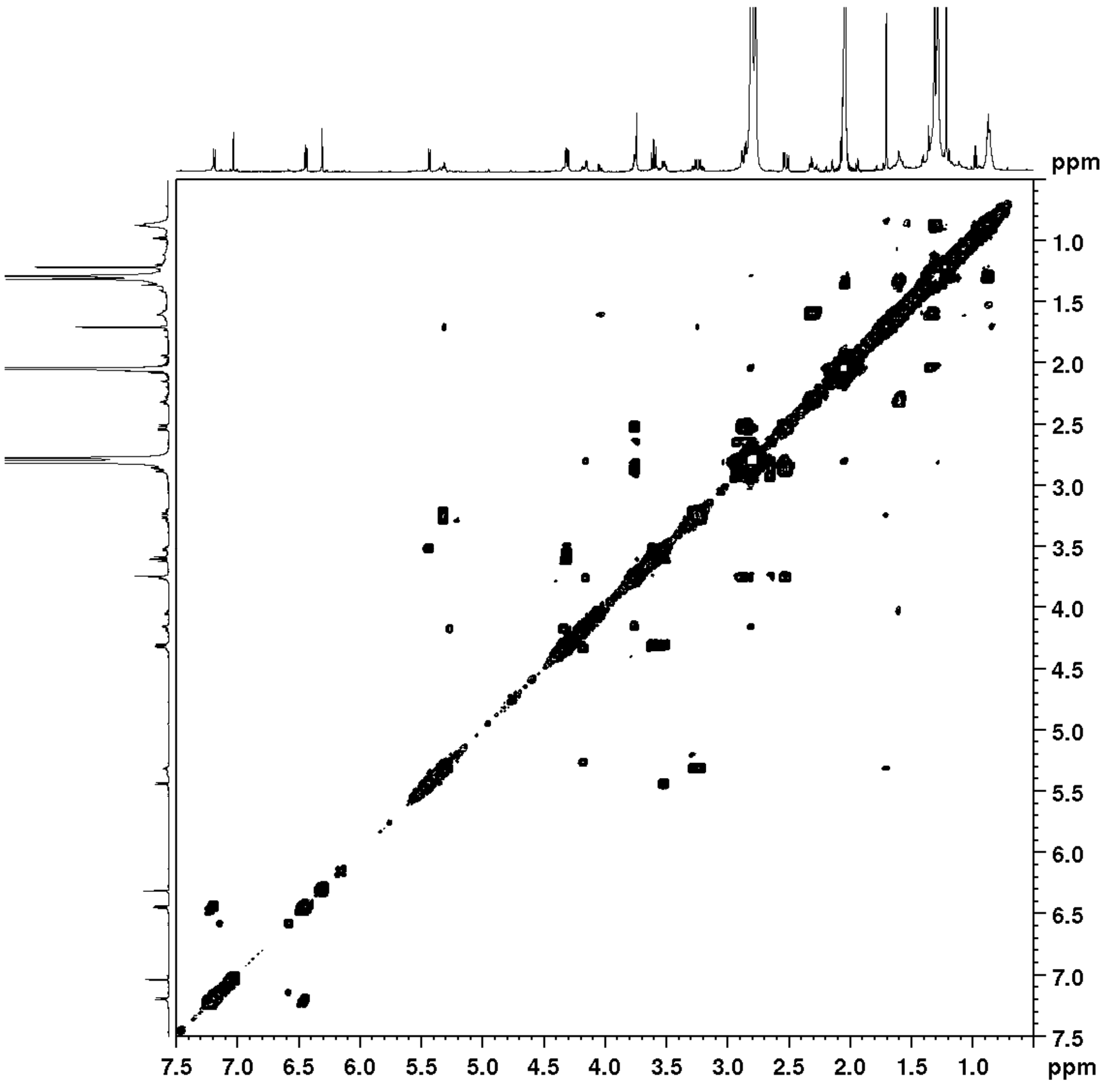

COSY spectrum of bituminarin B (**2**) (600 MHz, Acetone-*d*6)

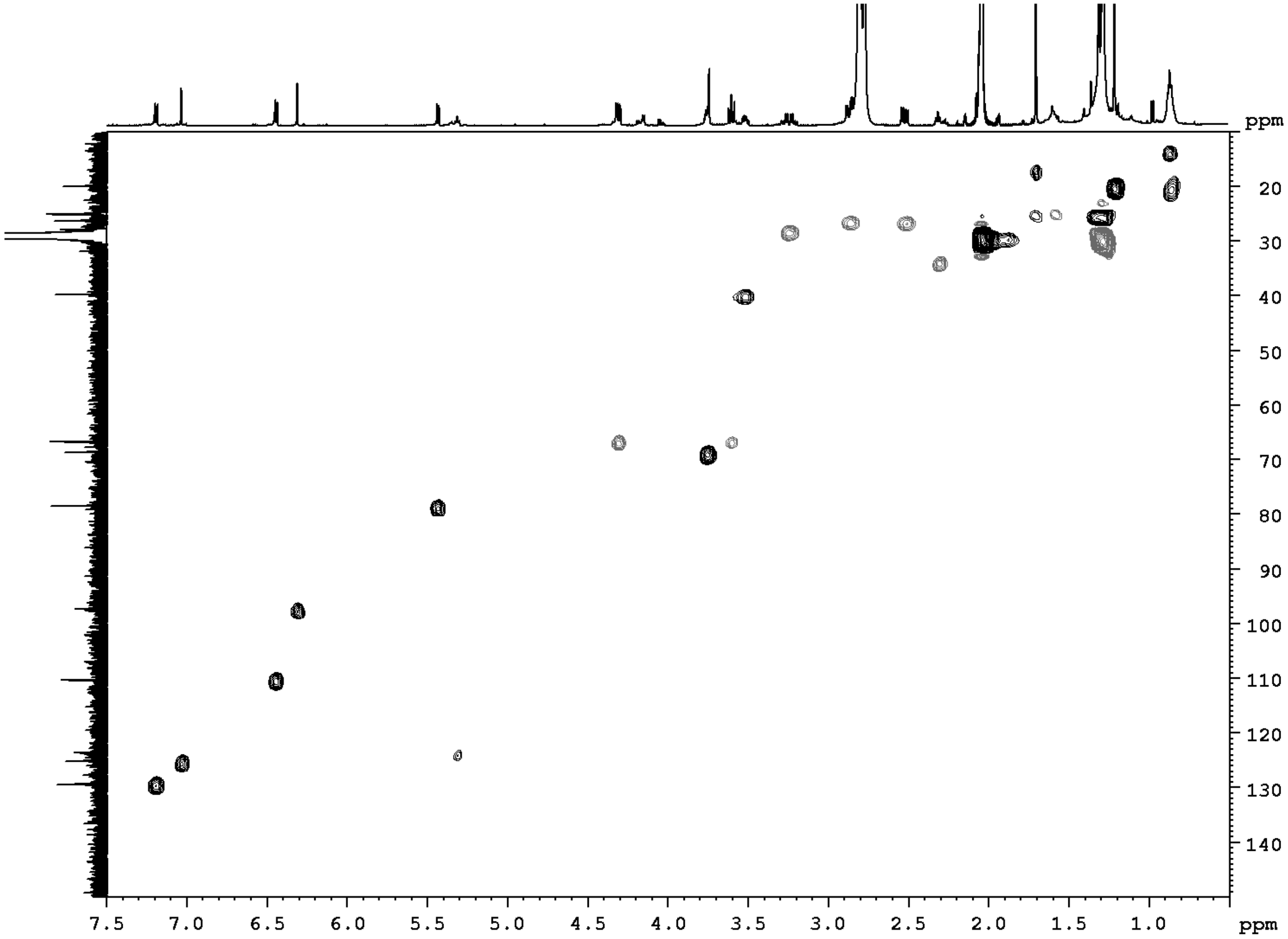

ed-HSQC spectrum of bituminarin B (**2**) (600 MHz, Acetone-*d6*)

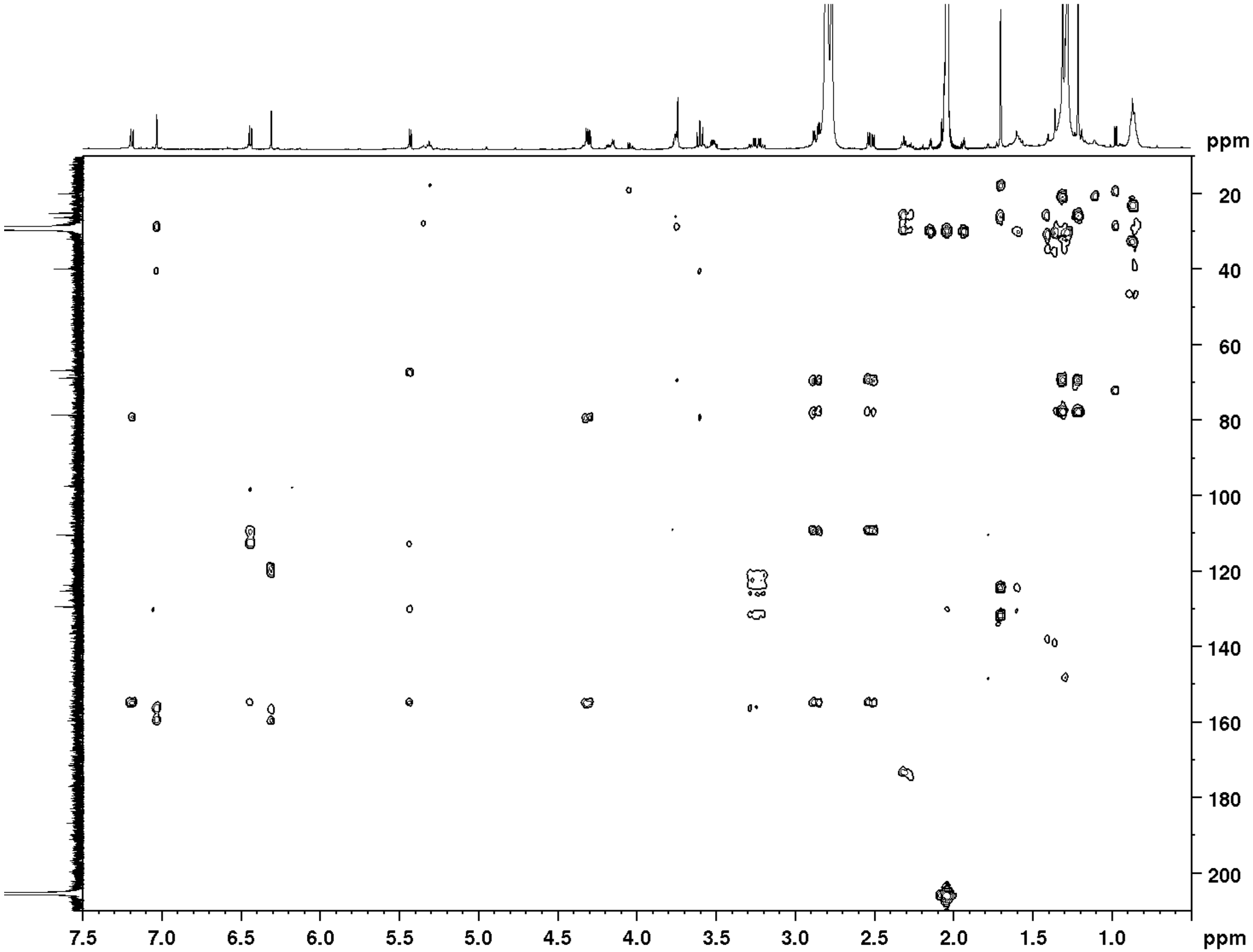

HMBC spectrum of bituminarin B (**2**) (600 MHz, Acetone-*d*6)

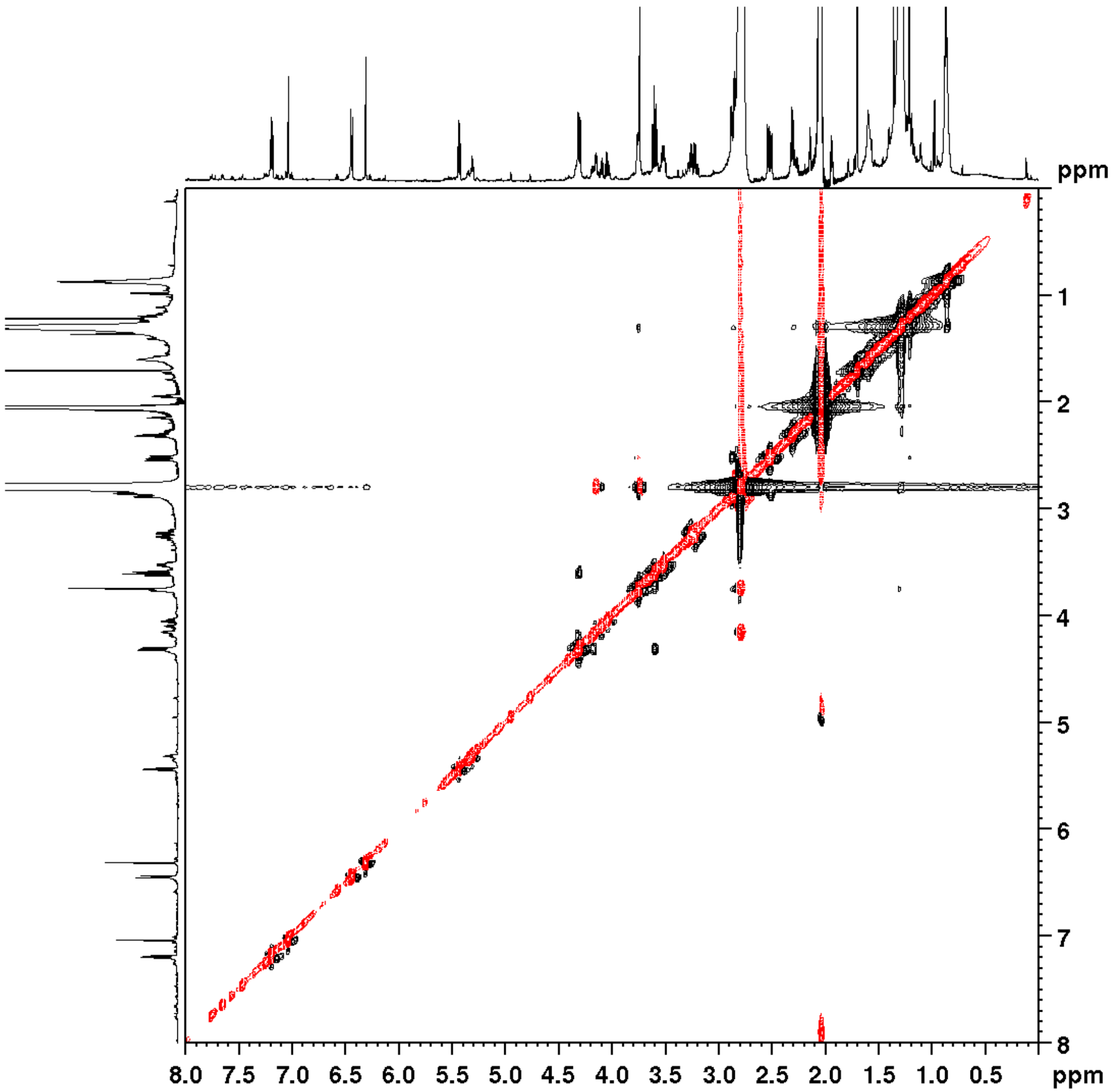

NOESY spectrum of bituminarin B (**2**) (600 MHz, Acetone-*d*6)

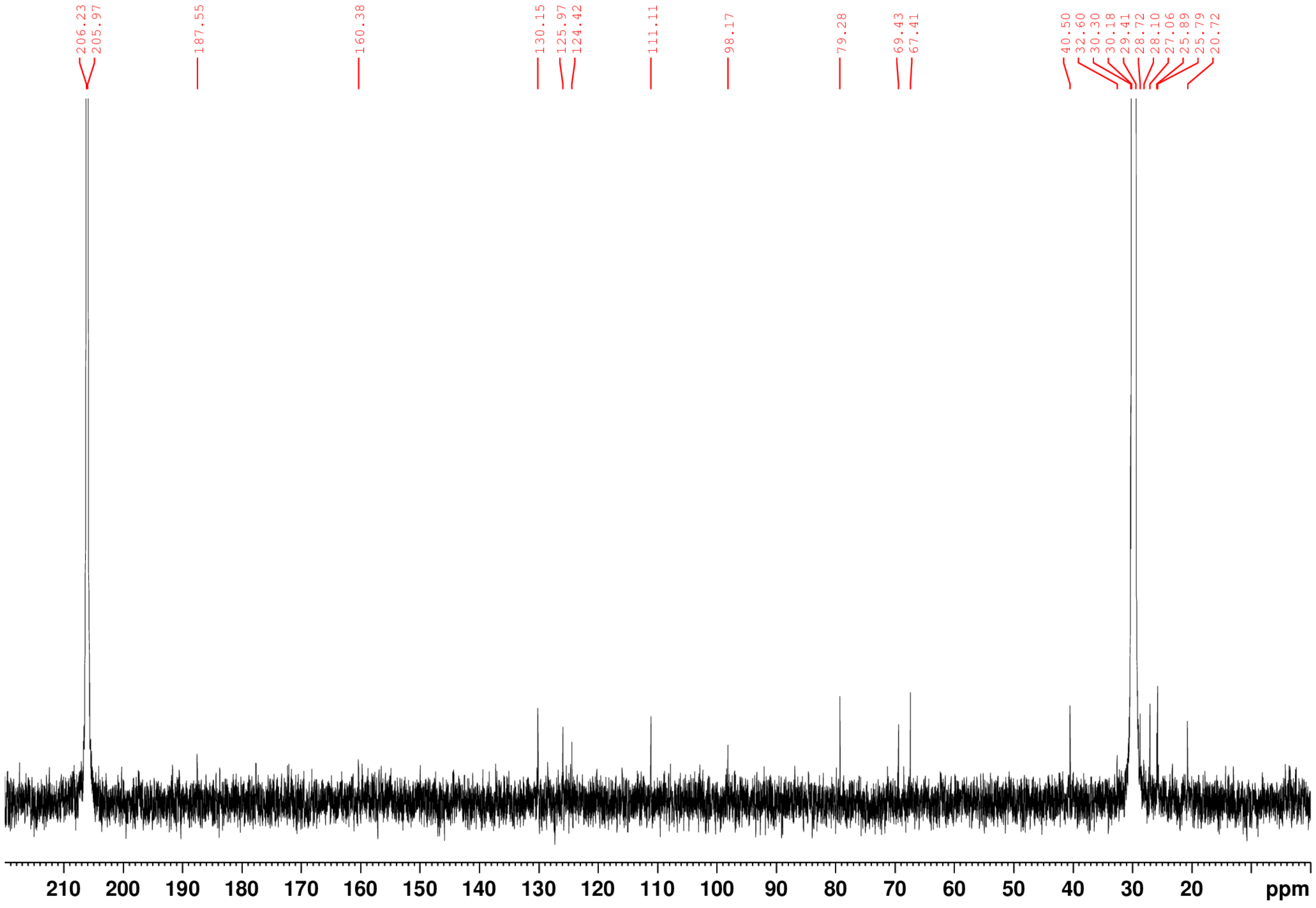

^{13}C NMR spectrum of bituminarin B (**2**) (150 MHz, Acetone-*d*6)

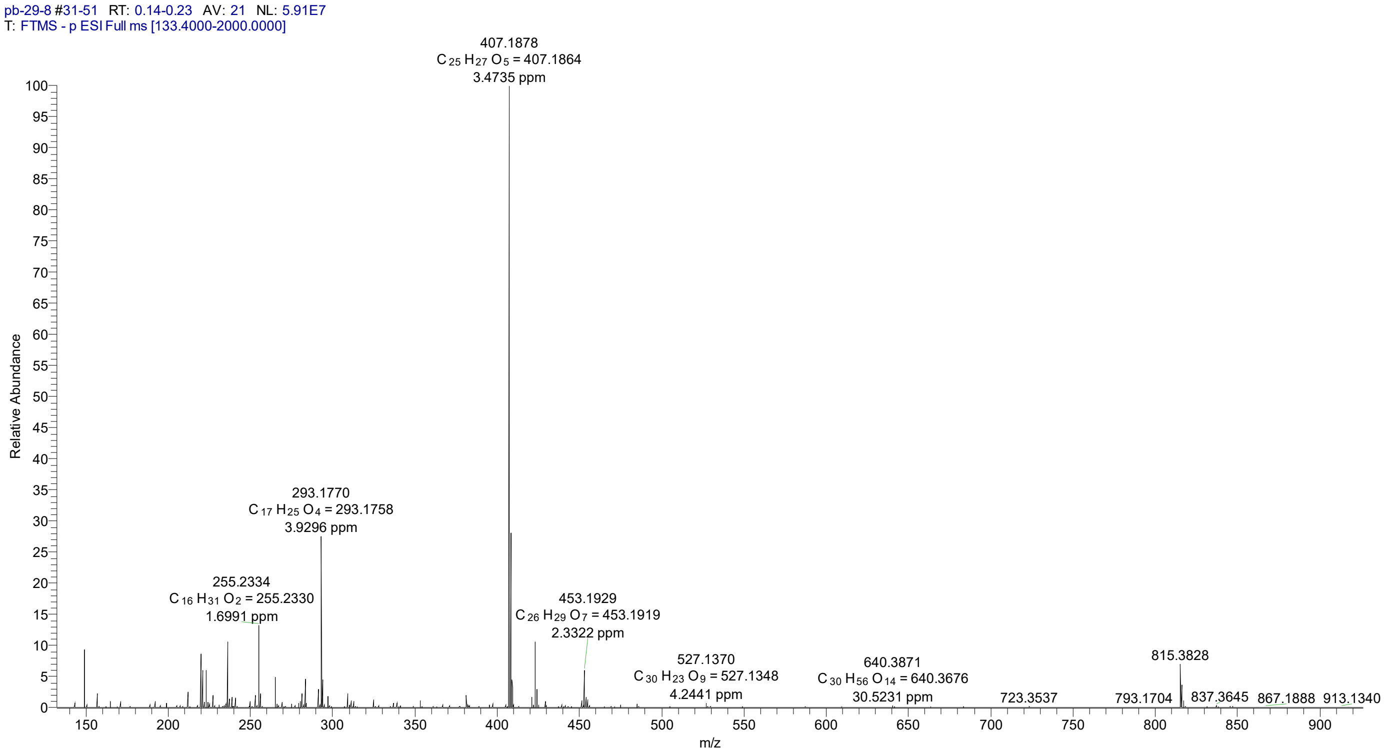

HR ESIMS spectrum of bituminarin B (**2**)

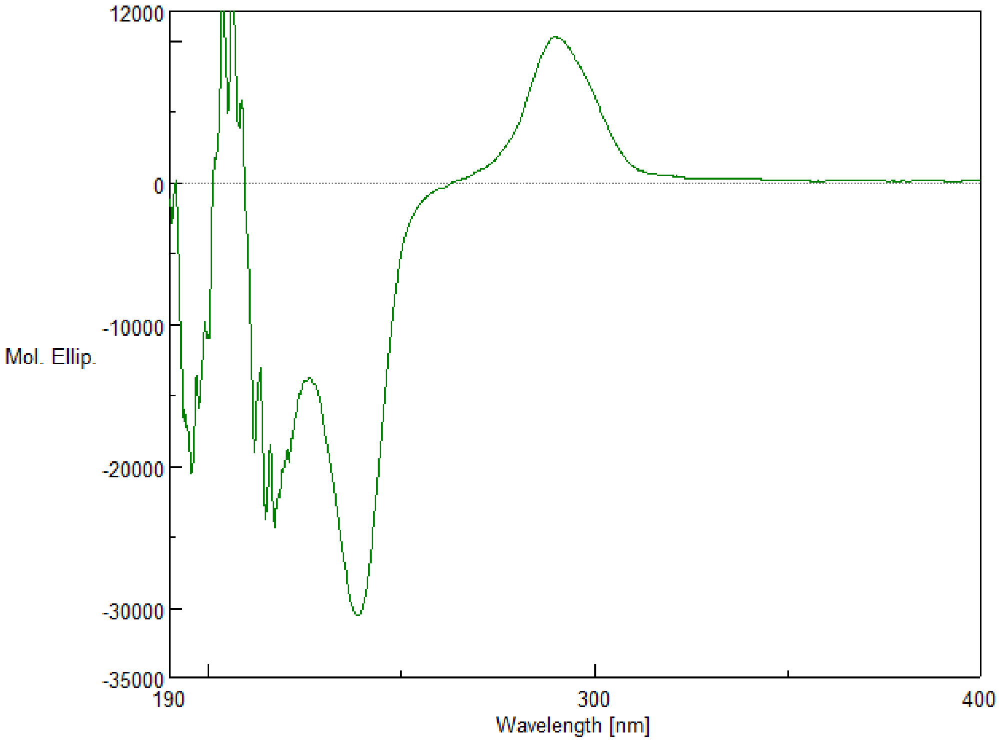

ECD curve of bituminarin B (**2**) (CH_3OH)

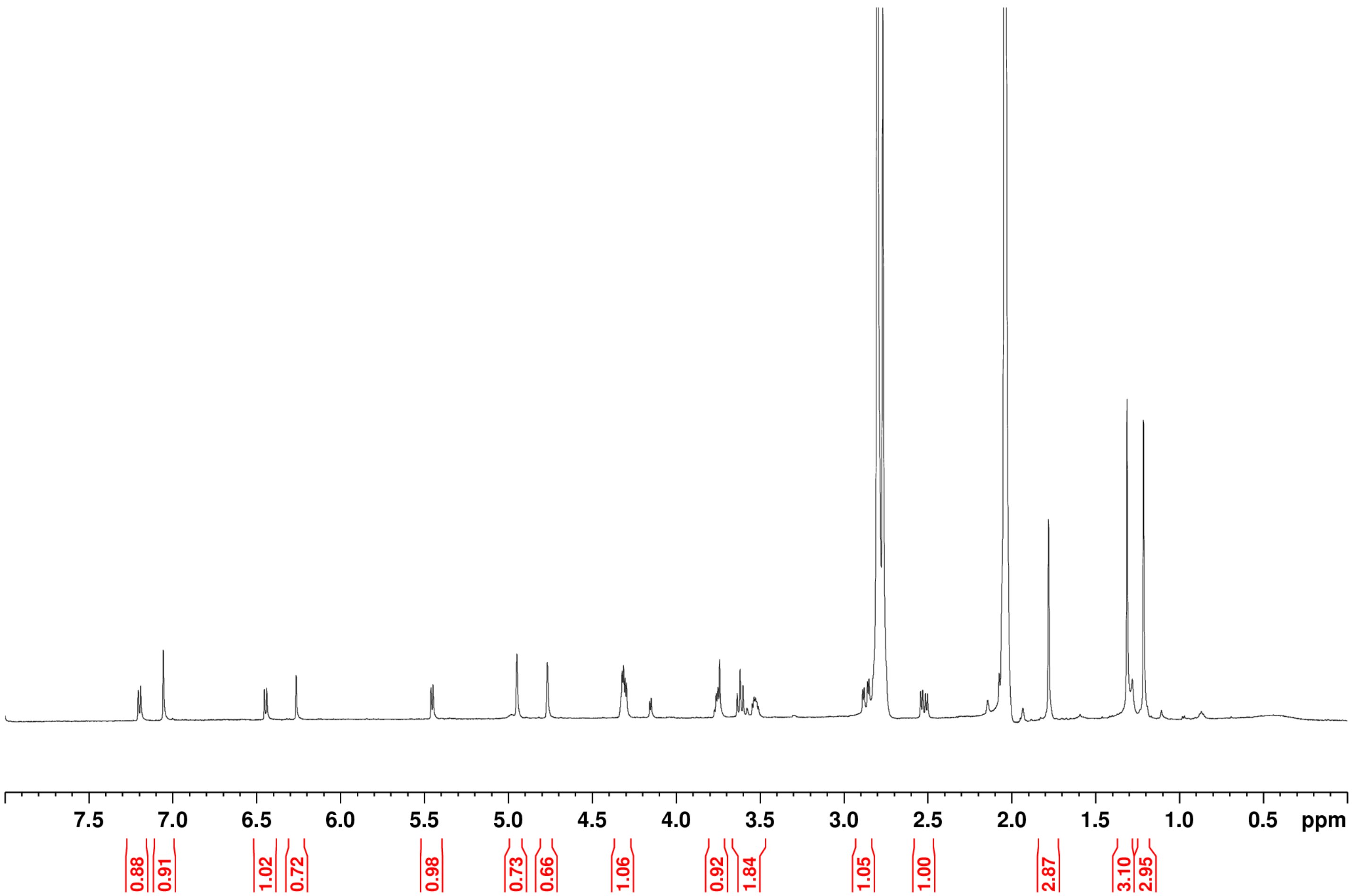

^{1}H NMR spectrum of bituminarin C (**3**) (600 MHz, Acetone-*d6*)

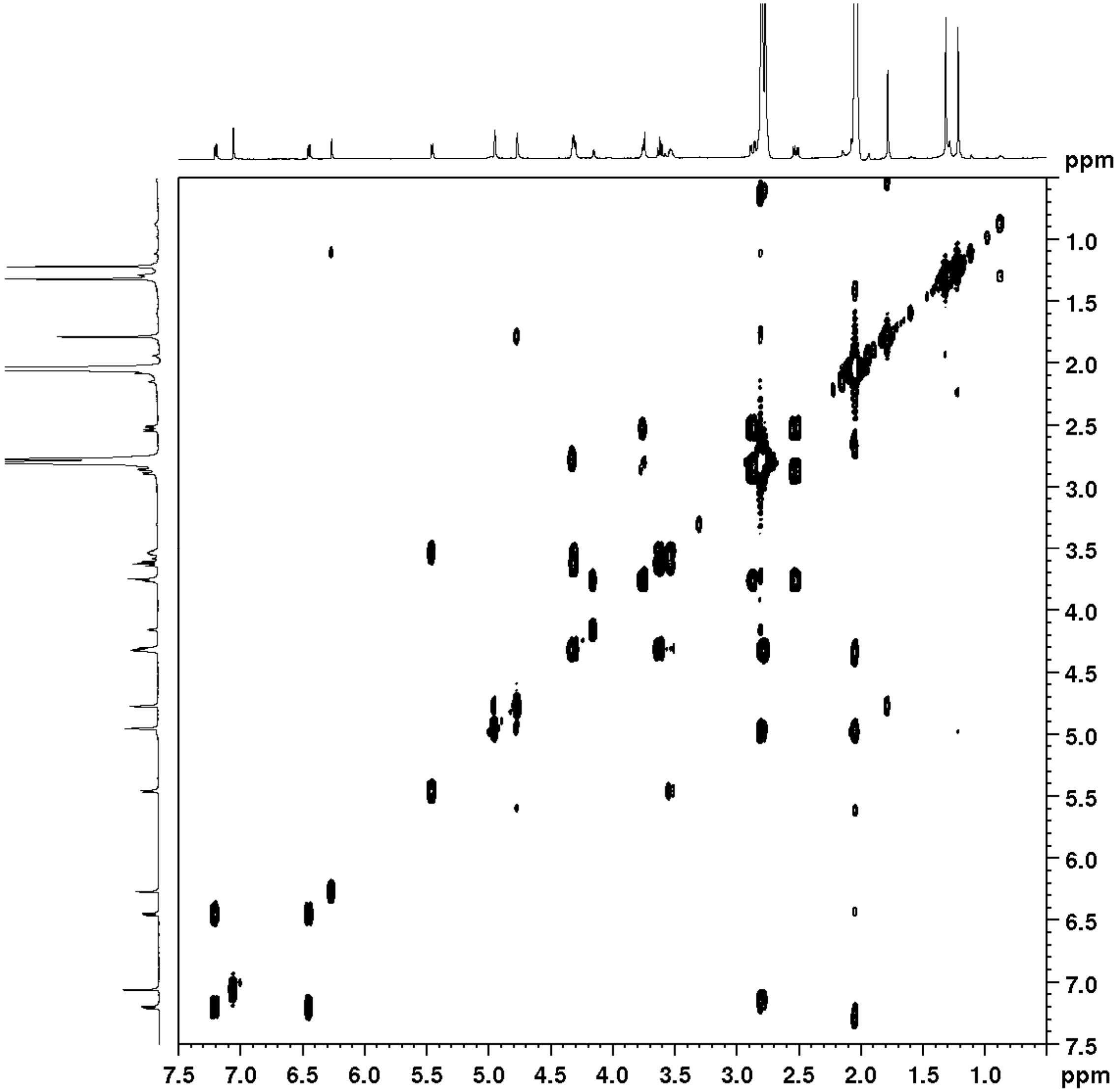

COSY spectrum of bituminarin C (**3**) (600 MHz, Acetone-*d*6)

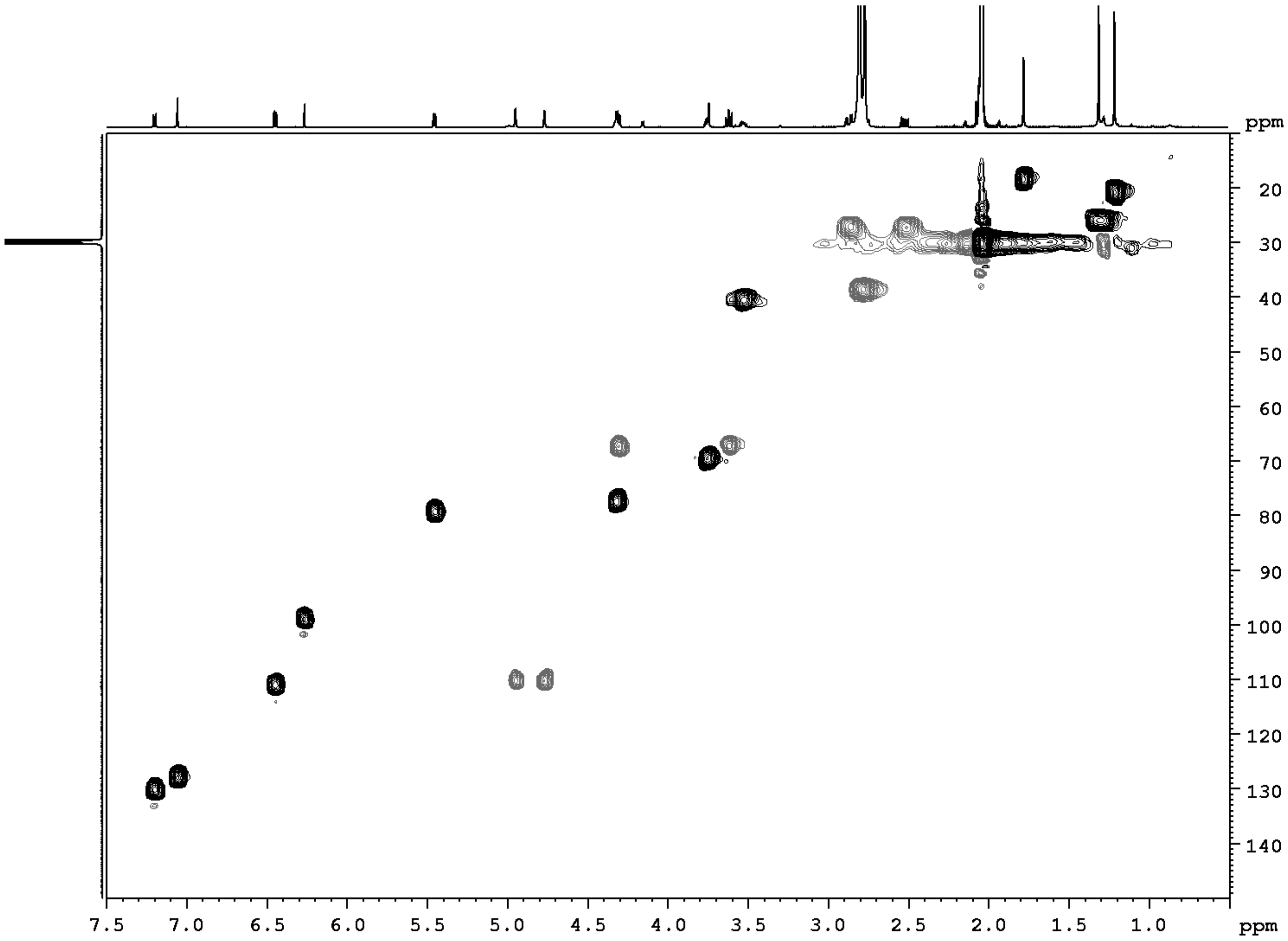

ed-HSQC spectrum of bituminarin C (**3**) (600 MHz, Acetone-*d*6)

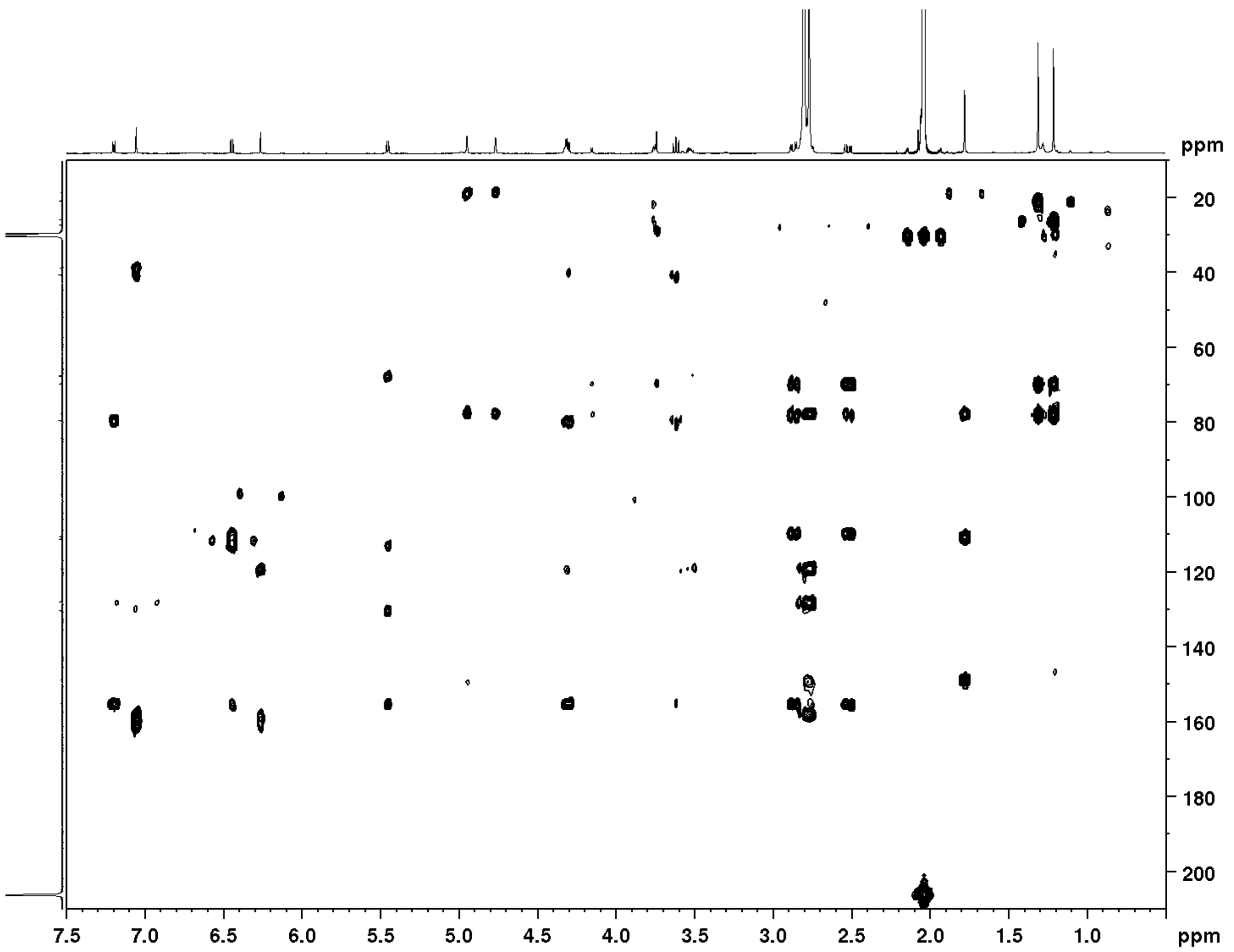

HMBC spectrum of bituminarin C (**3**) (600 MHz, Acetone-*d*6)

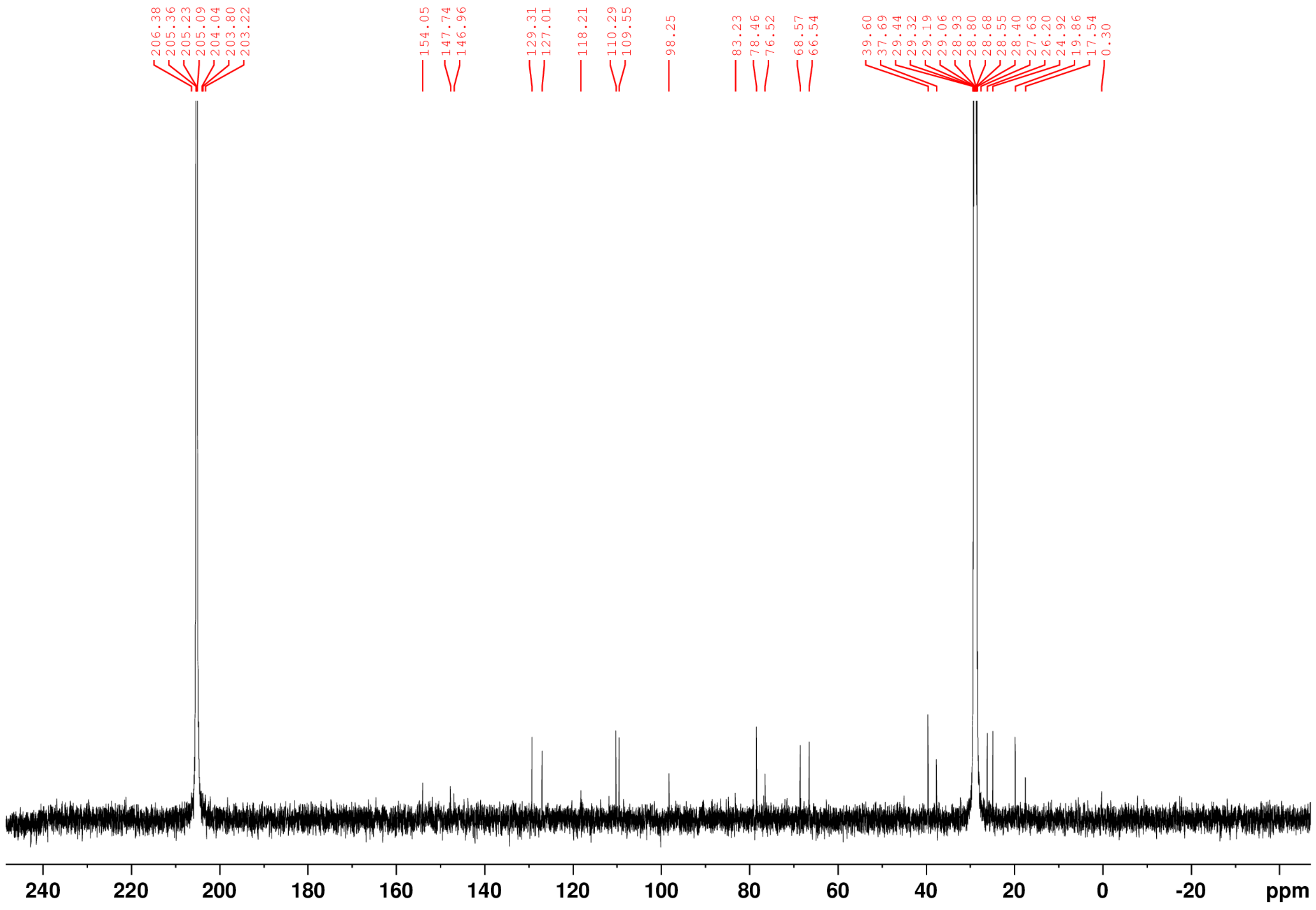

^{13}C NMR spectrum of bituminarin C (**3**) (150 MHz, Acetone-*d6*)

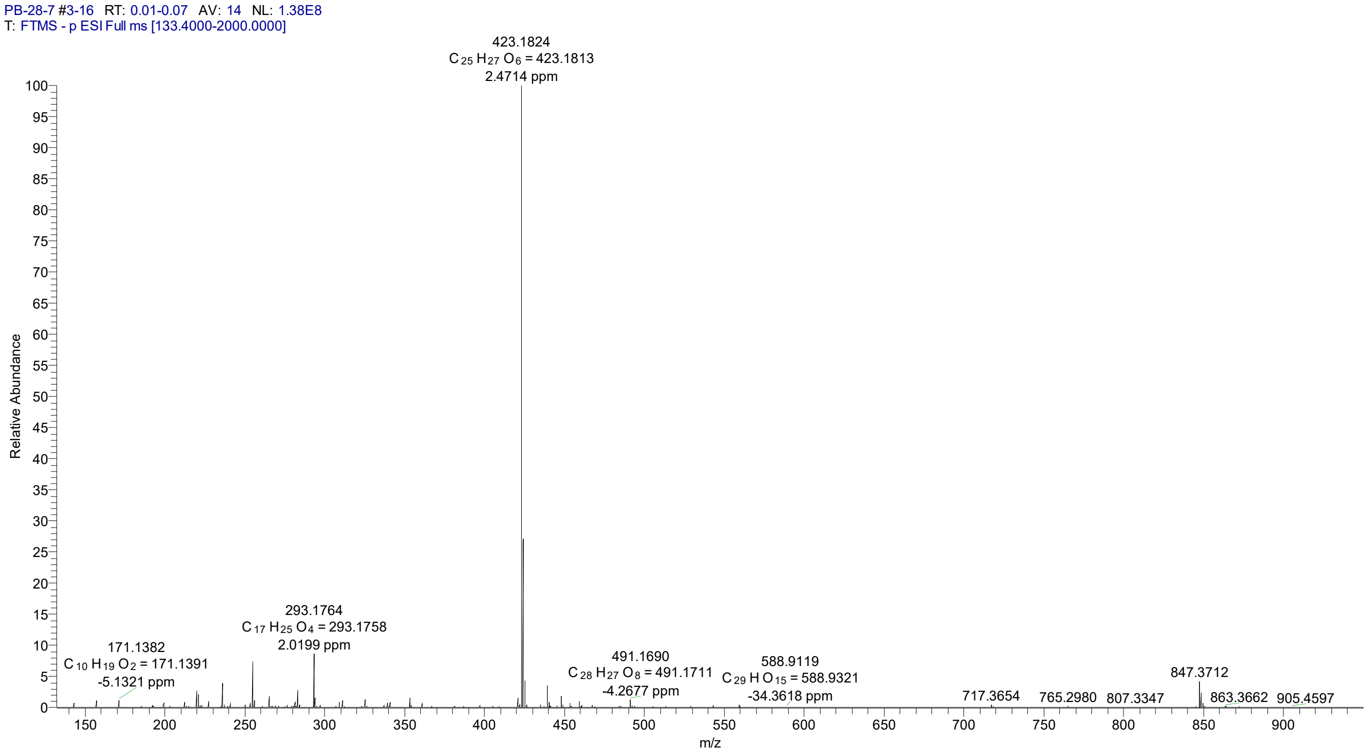

HR ESIMS spectrum of bituminarin C (**3**)

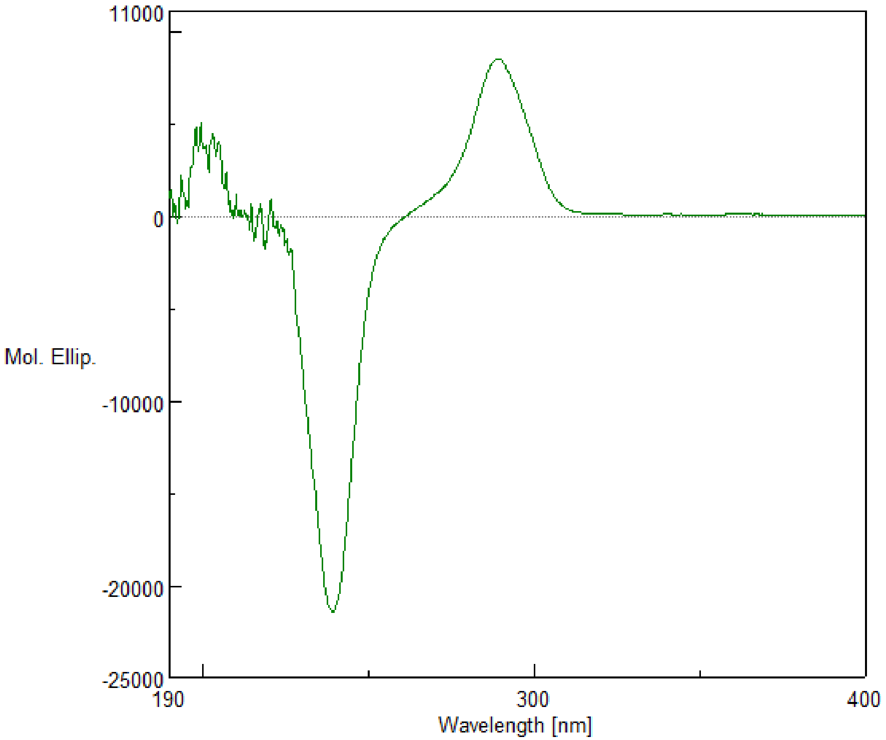

ECD curve of bituminarin C (**3**)

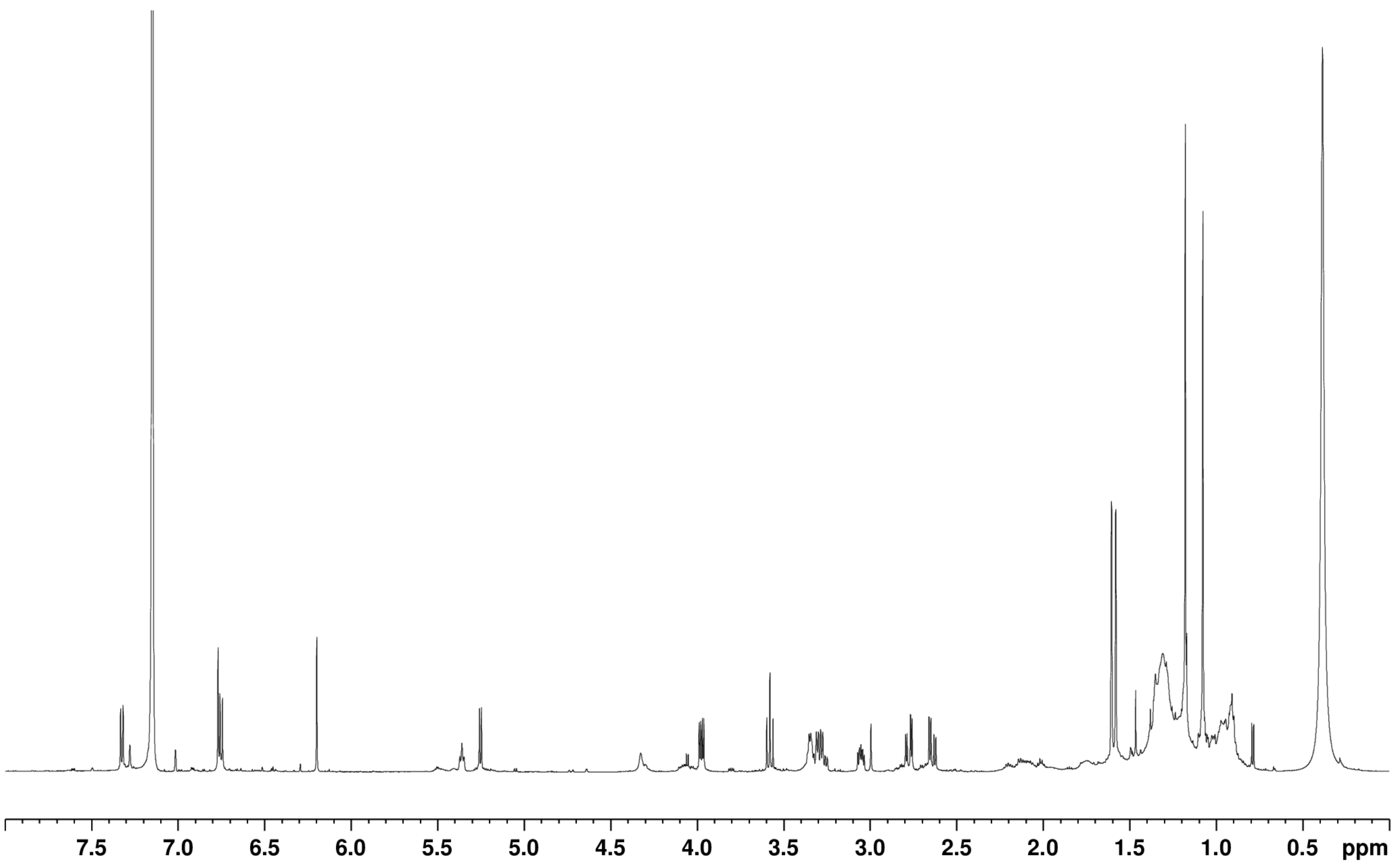

^{1}H NMR spectrum of bituminarin A (**1**) (600 MHz, C_6D_6)

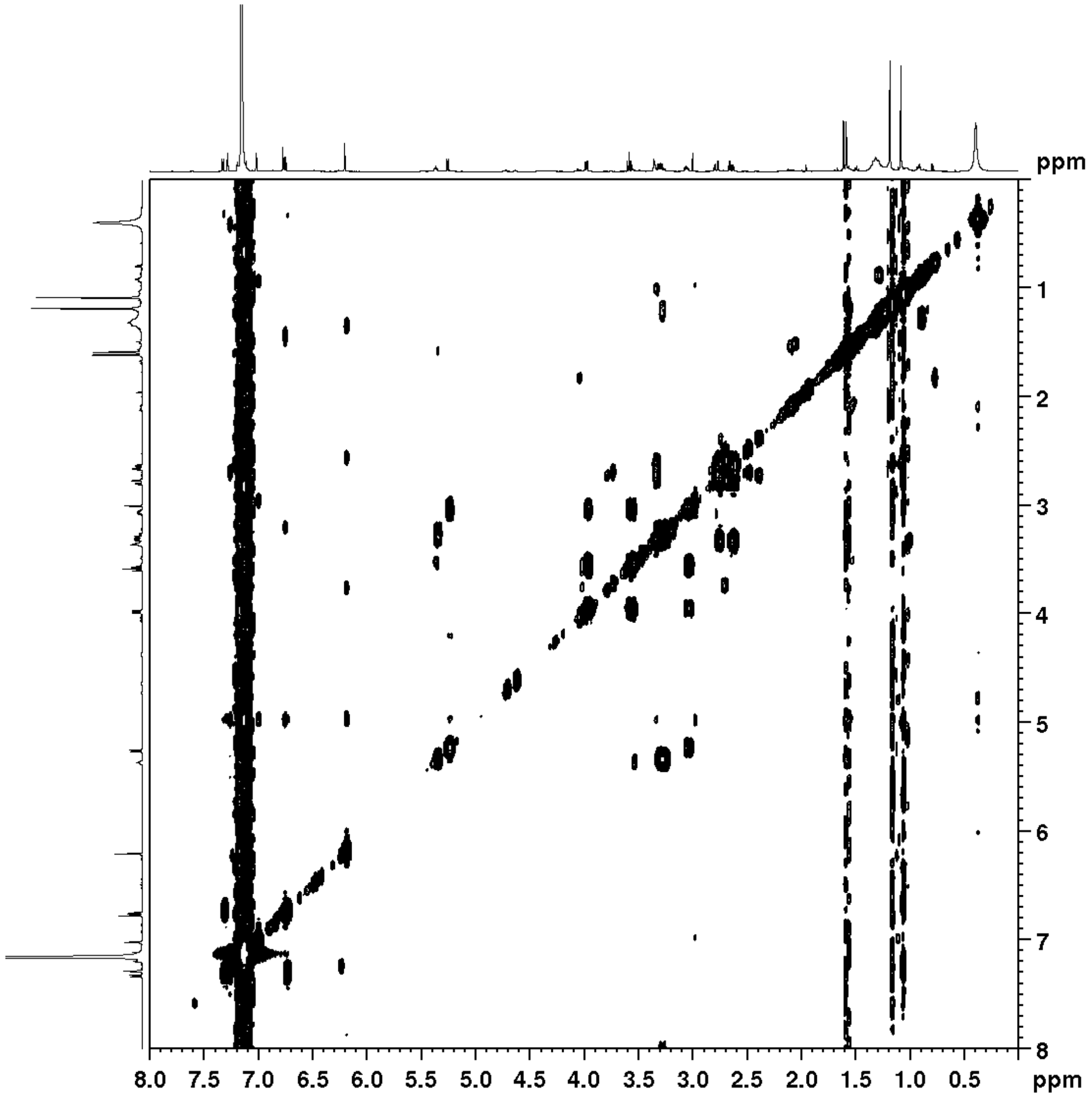

COSY spectrum of bituminarin A (**1**) (600 MHz, C_6D_6)

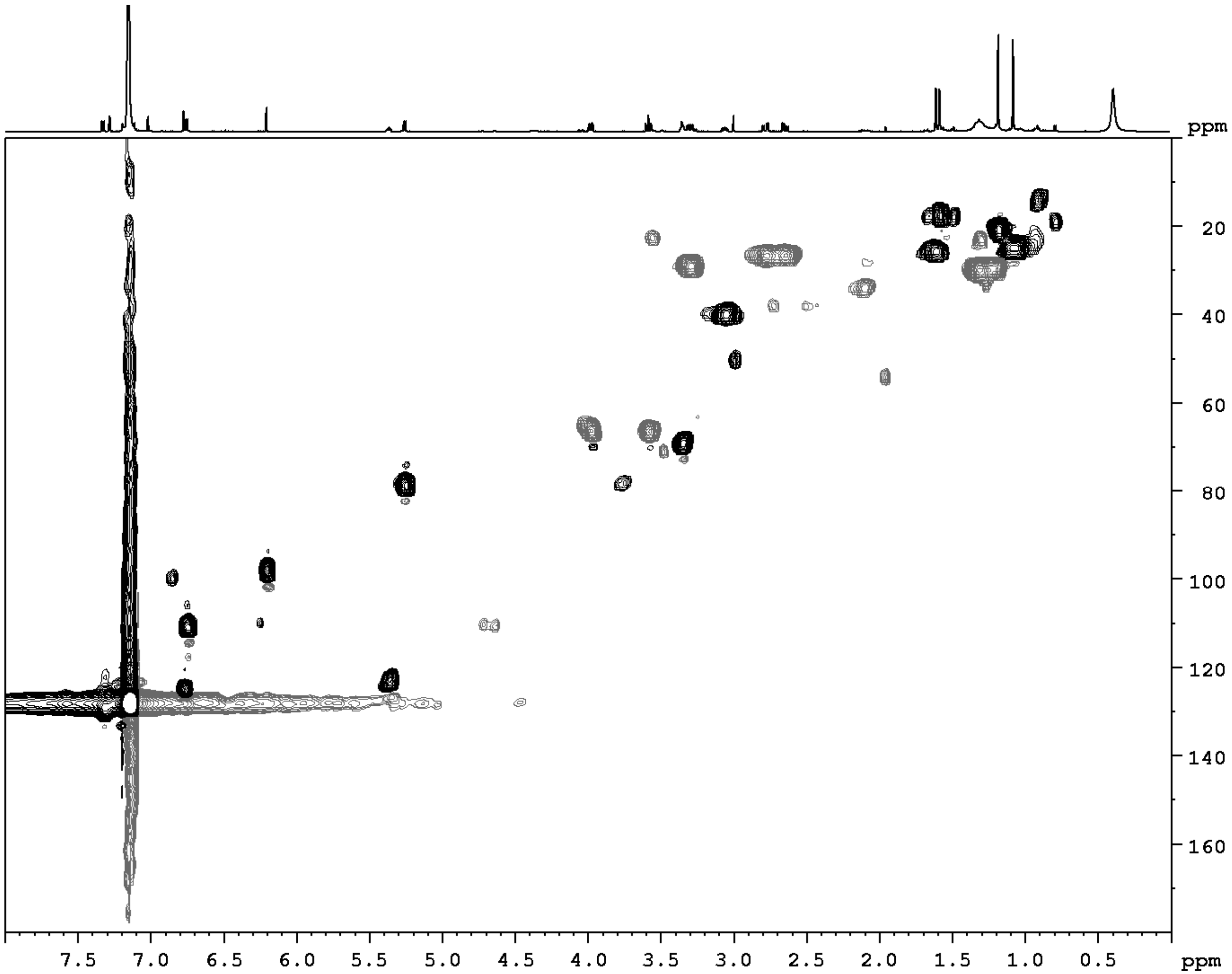

ed-HSQC spectrum of bituminarin A (**1**) (600 MHz, C_6D_6)

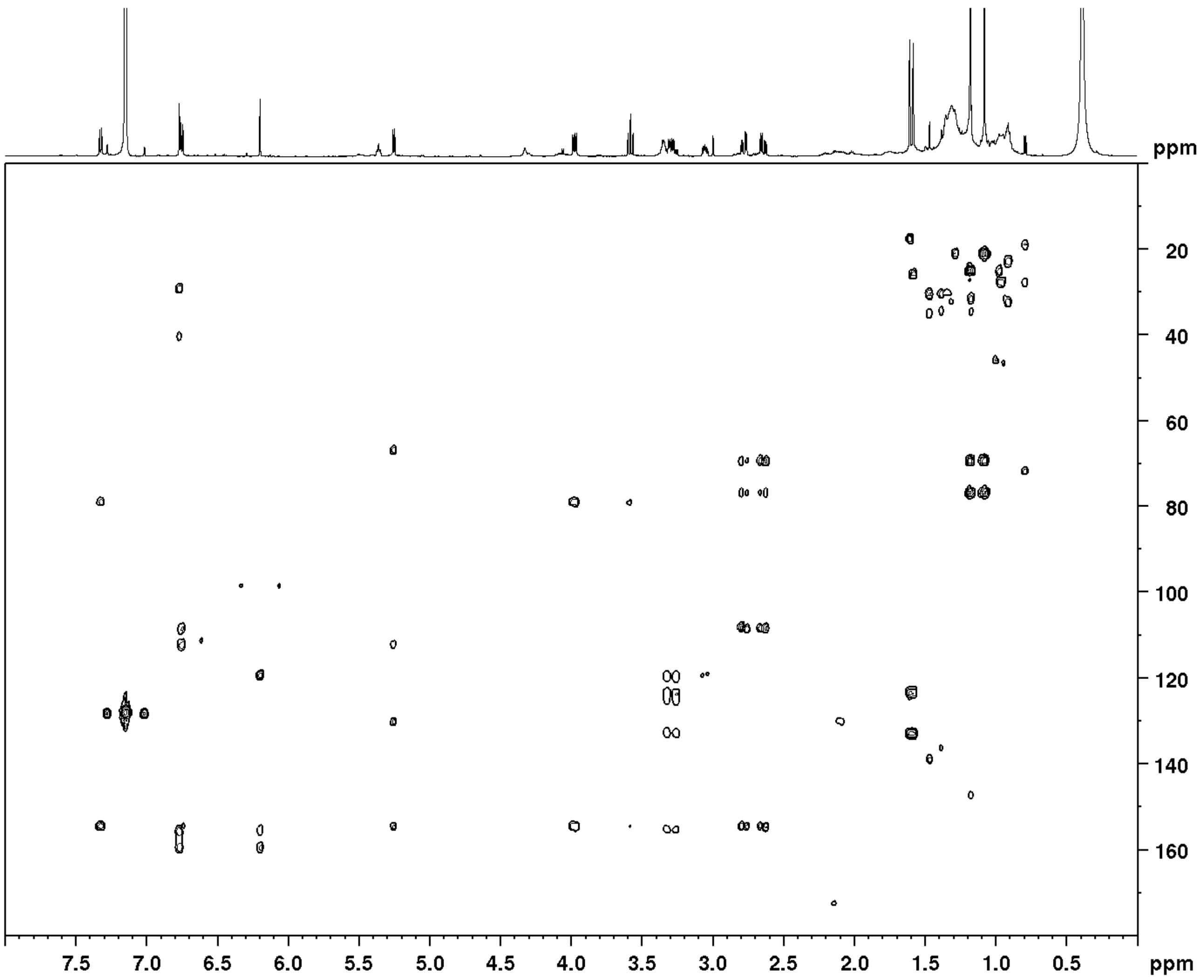

HMBC spectrum of bituminarin A (**1**) (600 MHz, C_6D_6)

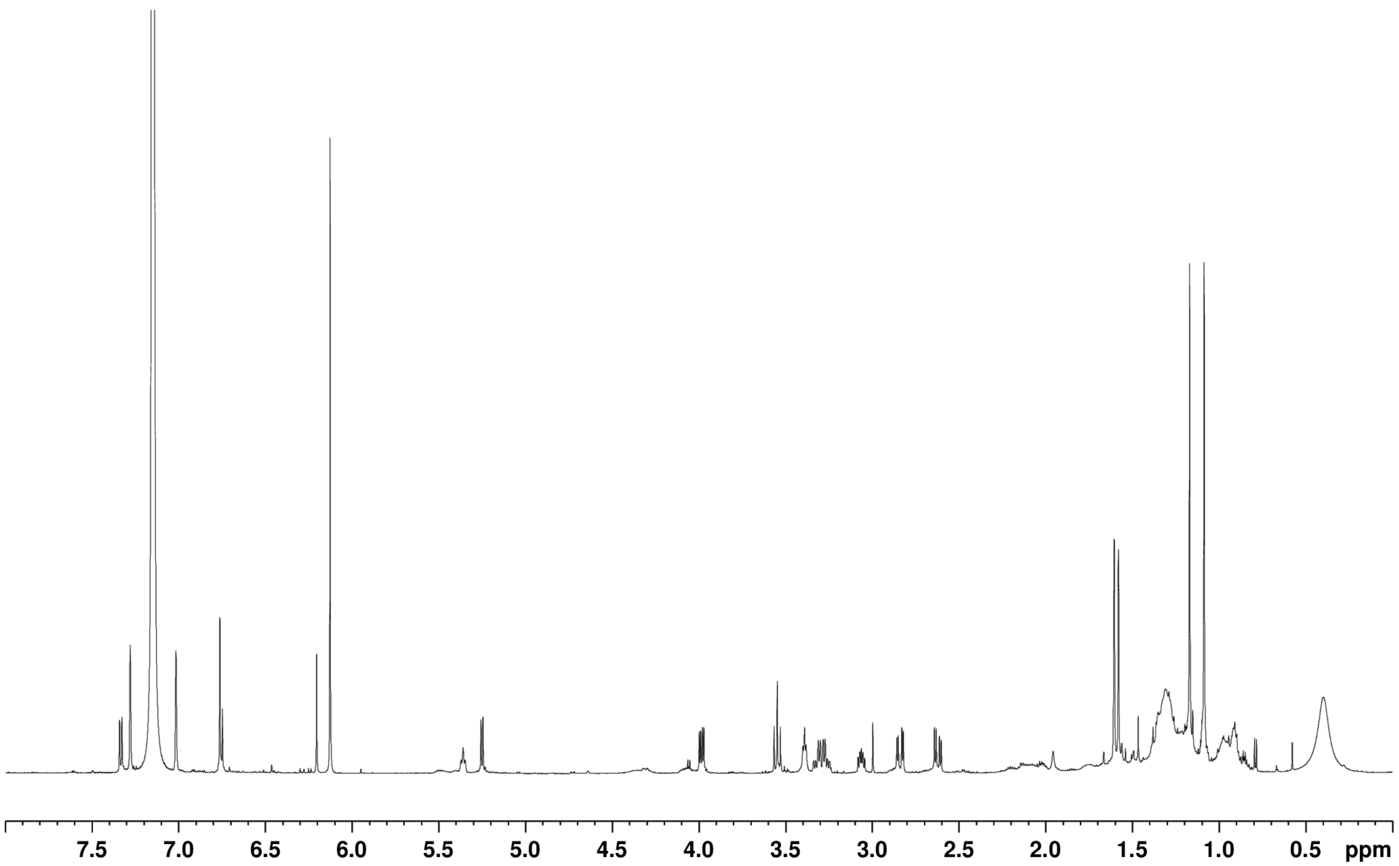

^{1}H NMR spectrum of bituminarin B (**2**) (600 MHz, C_6D_6)

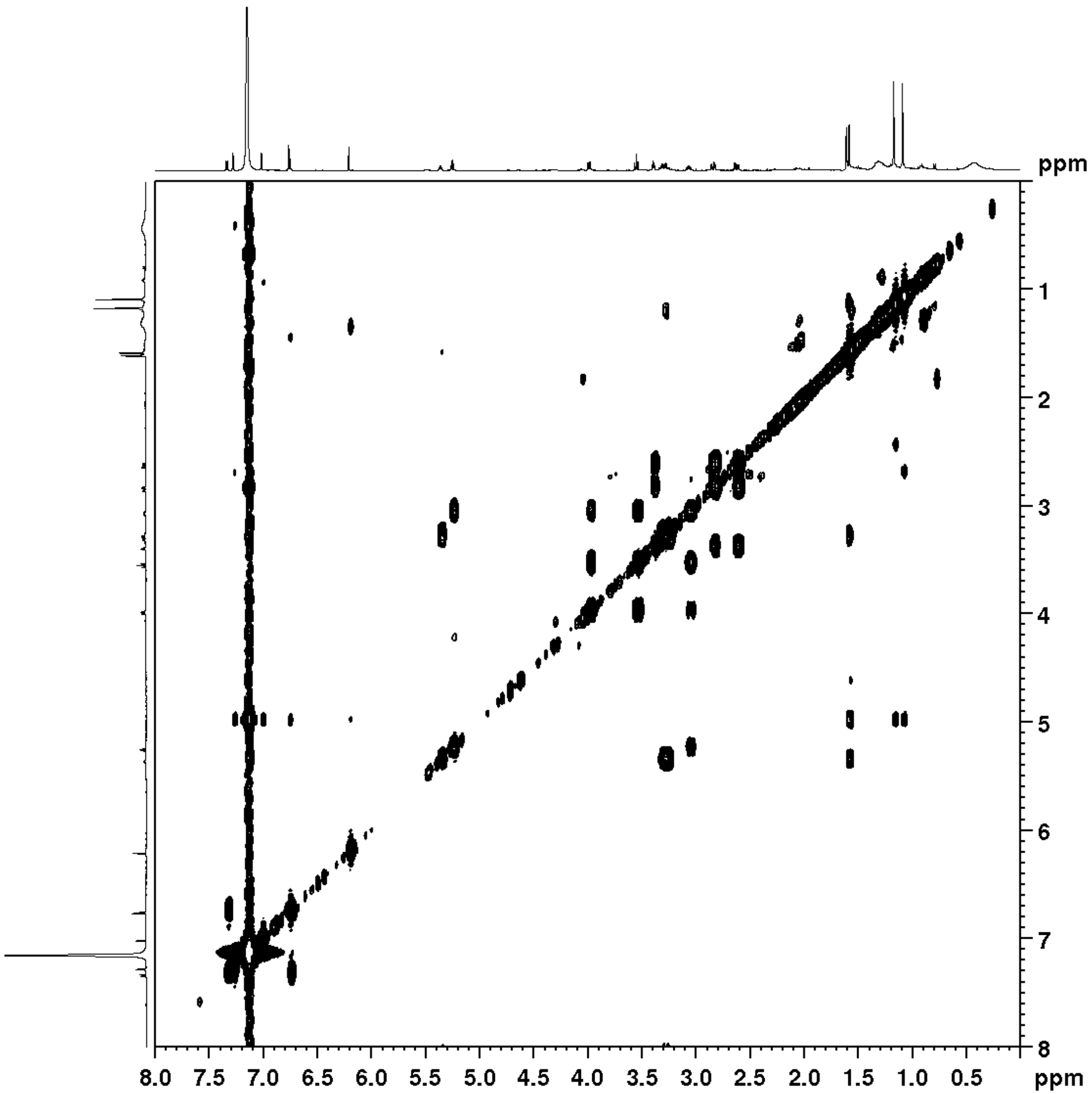

COSY spectrum of bituminarin B (**2**) (600 MHz, C_6D_6)

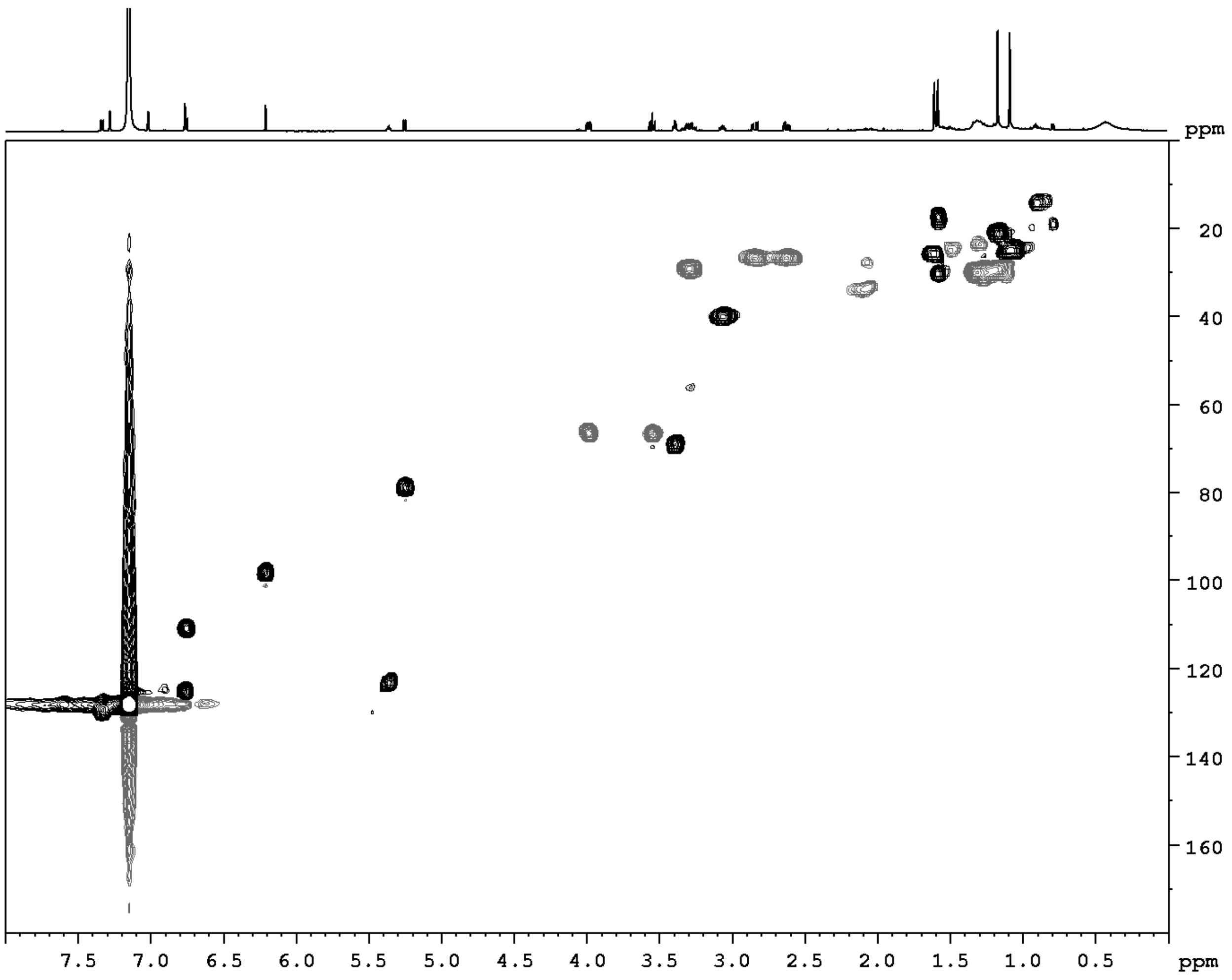

ed-HSQC spectrum of bituminarin B (**2**) (600 MHz C_6D_6)

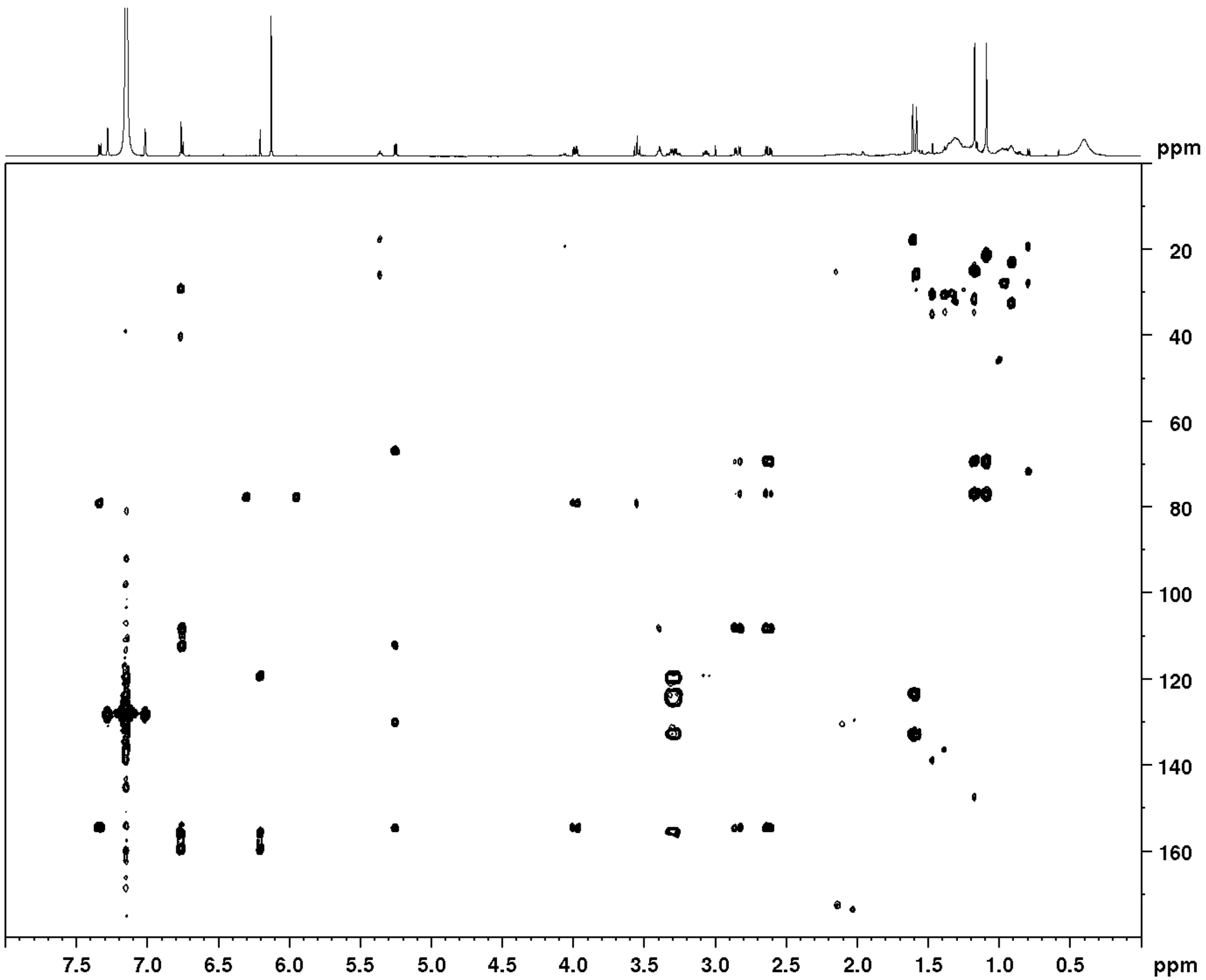

HMBC spectrum of bituminarin B (**2**) (600 MHz, C_6D_6)

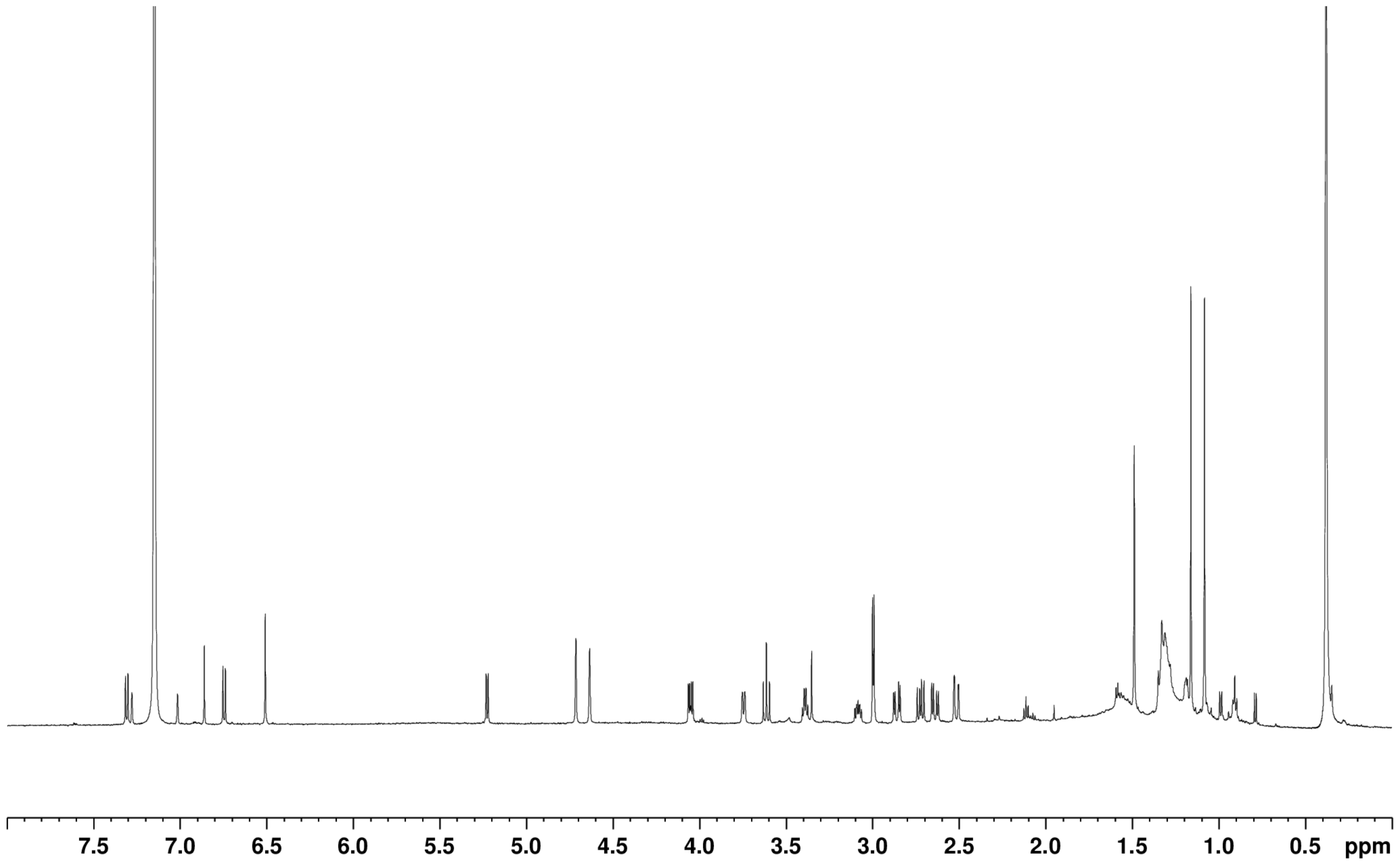

^{1}H NMR spectrum of bituminarin C (**3**) (600 MHz, C_6D_6)

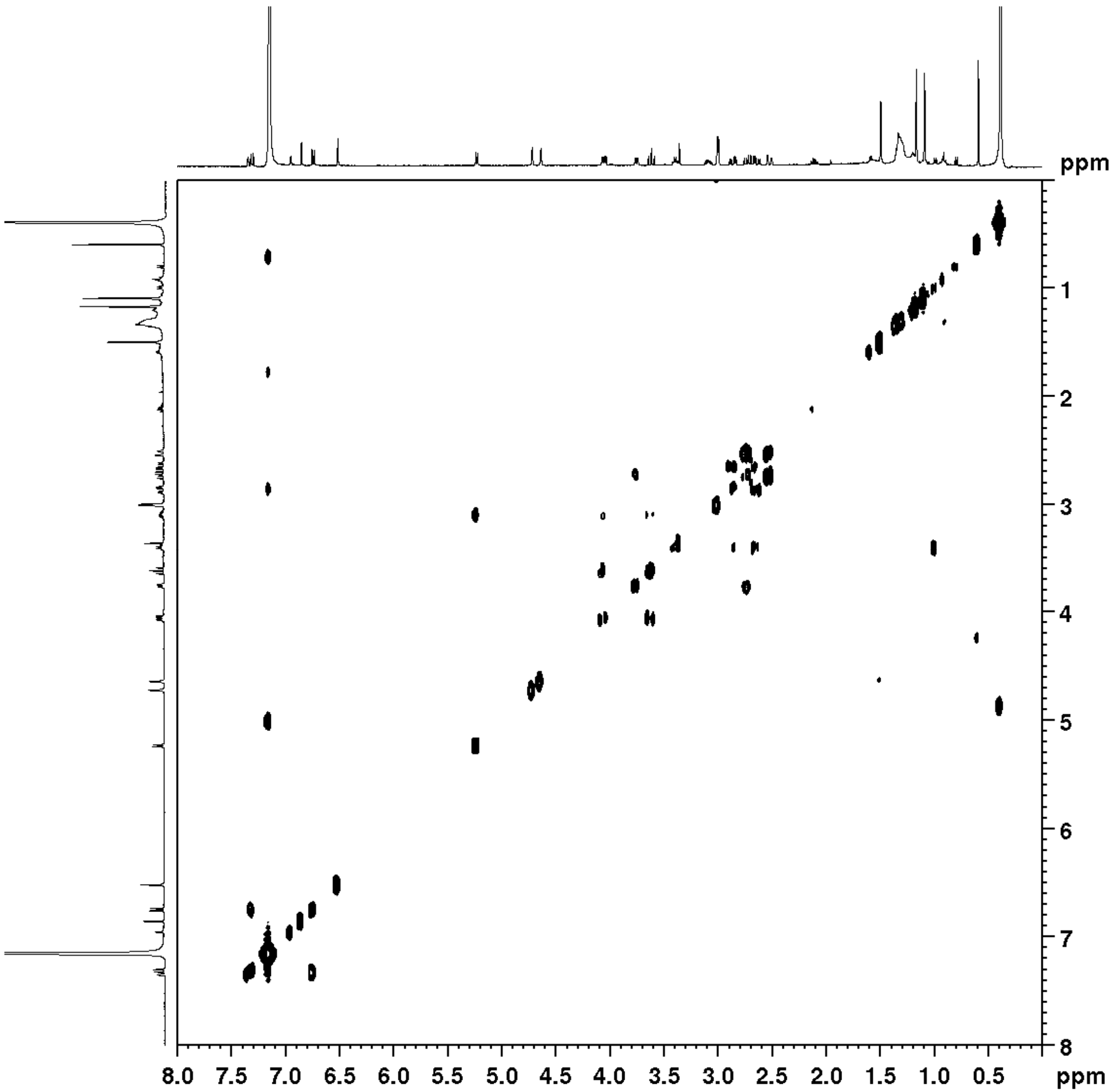

COSY spectrum of bituminarin C (**3**) (400 MHz, C_6D_6)

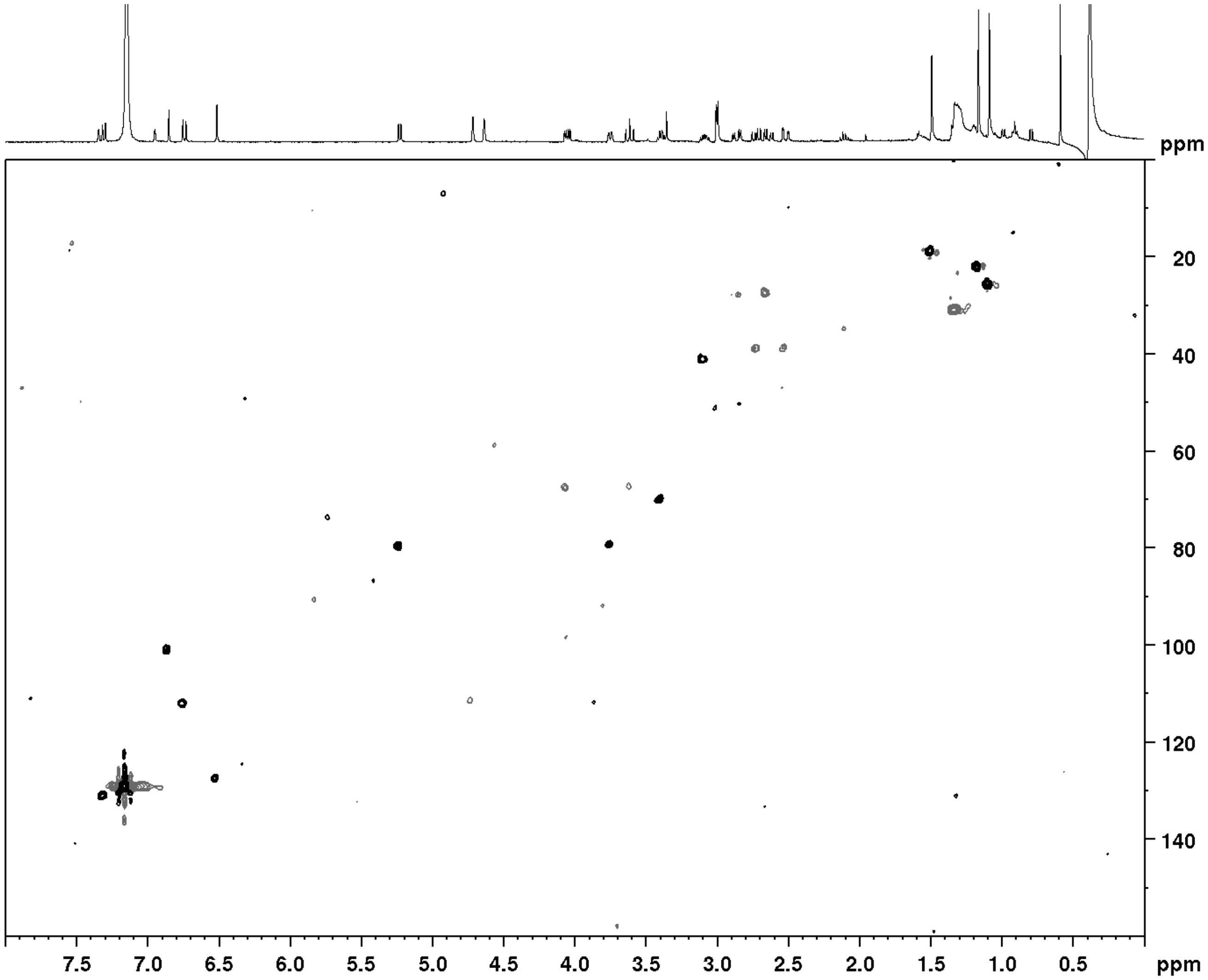

ed-HSQC spectrum of bituminarin C (**3**) (400 MHz, C_6D_6)

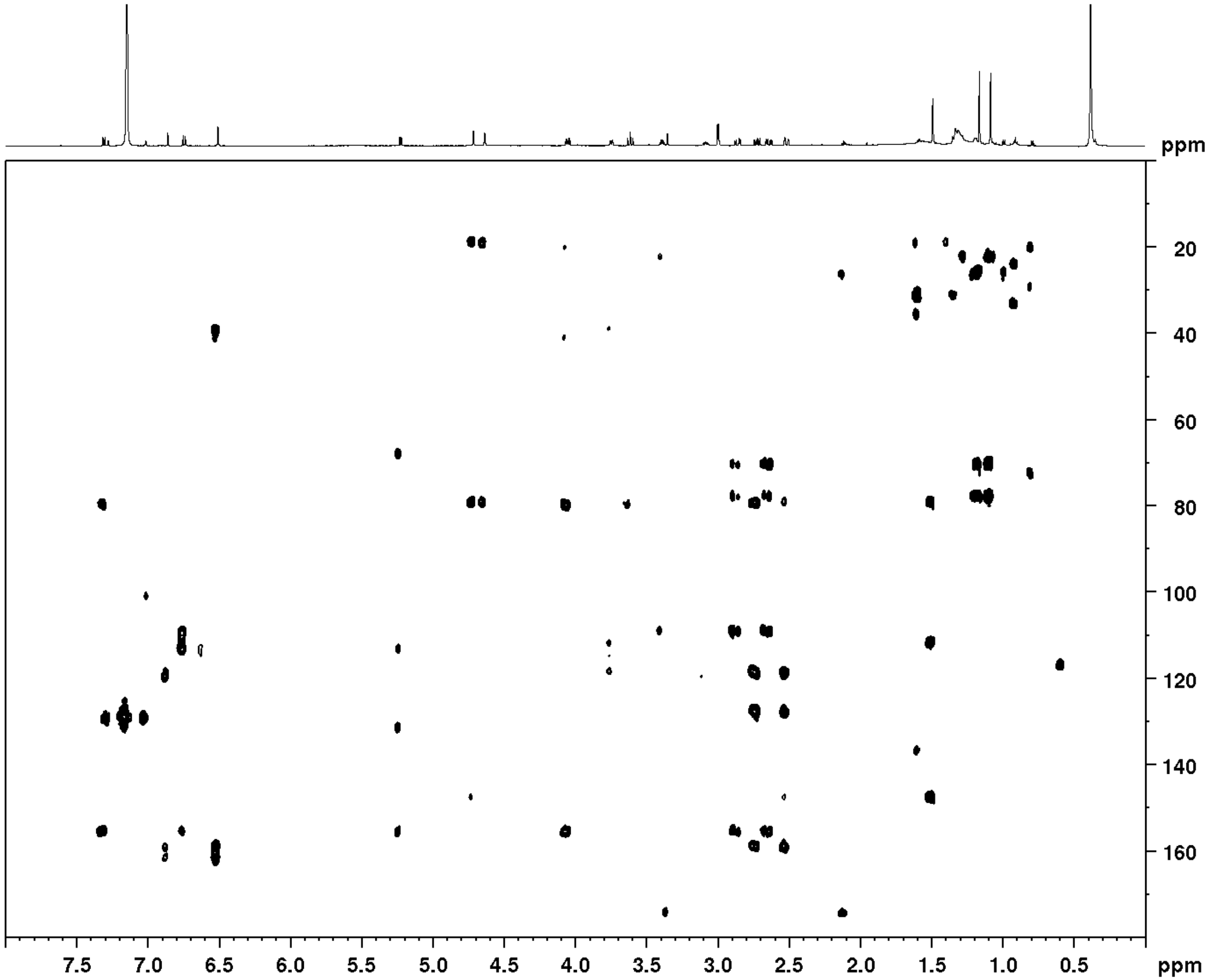

HMBC spectrum of bituminarin C (**3**) (600 MHz, C_6D_6)

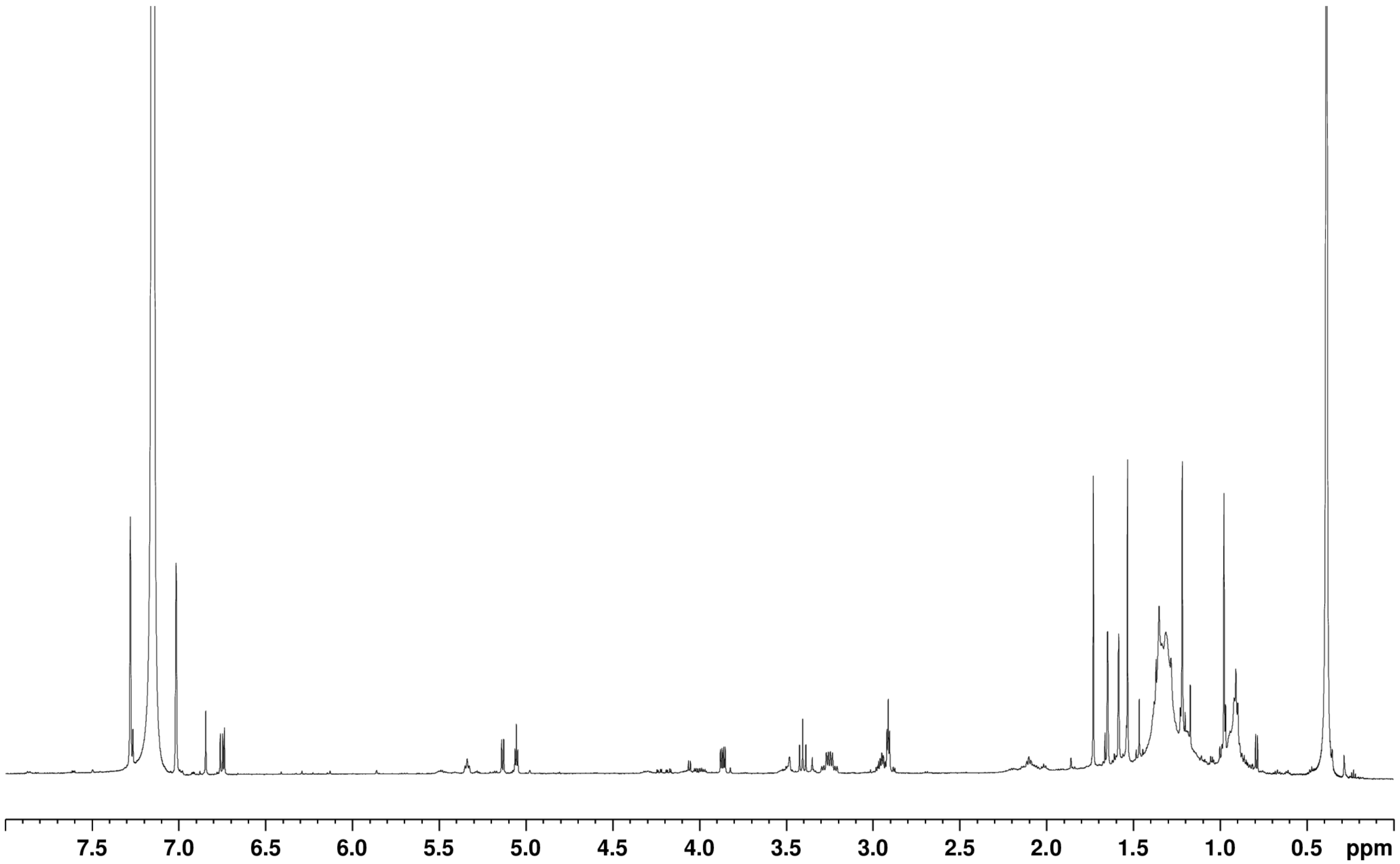

^{1}H NMR spectrum of acetylated bituminarin A (**1a**) (600 MHz, C_6D_6)

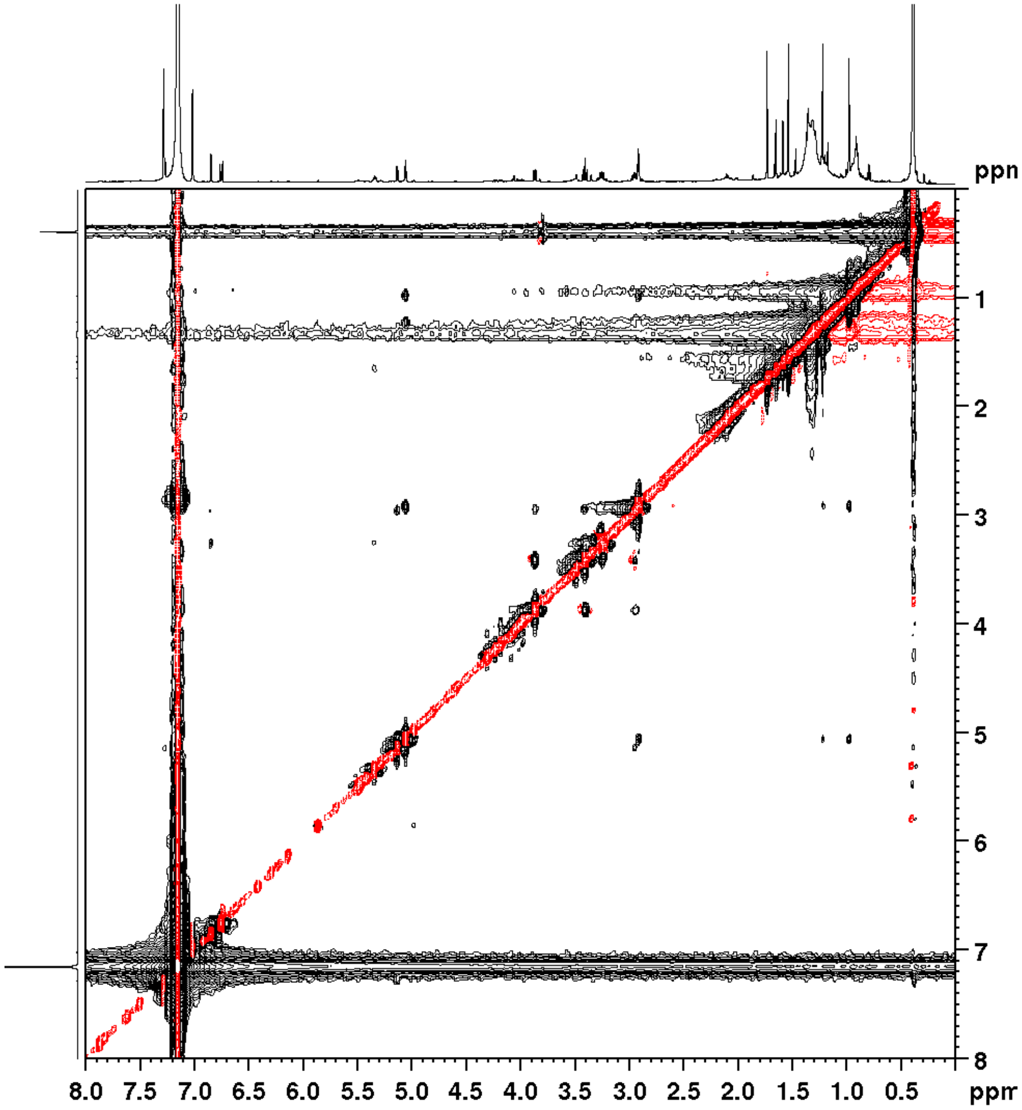

NOESY spectrum of acetylated bituminarin A (**1a**) (600 MHz, C_6D_6)

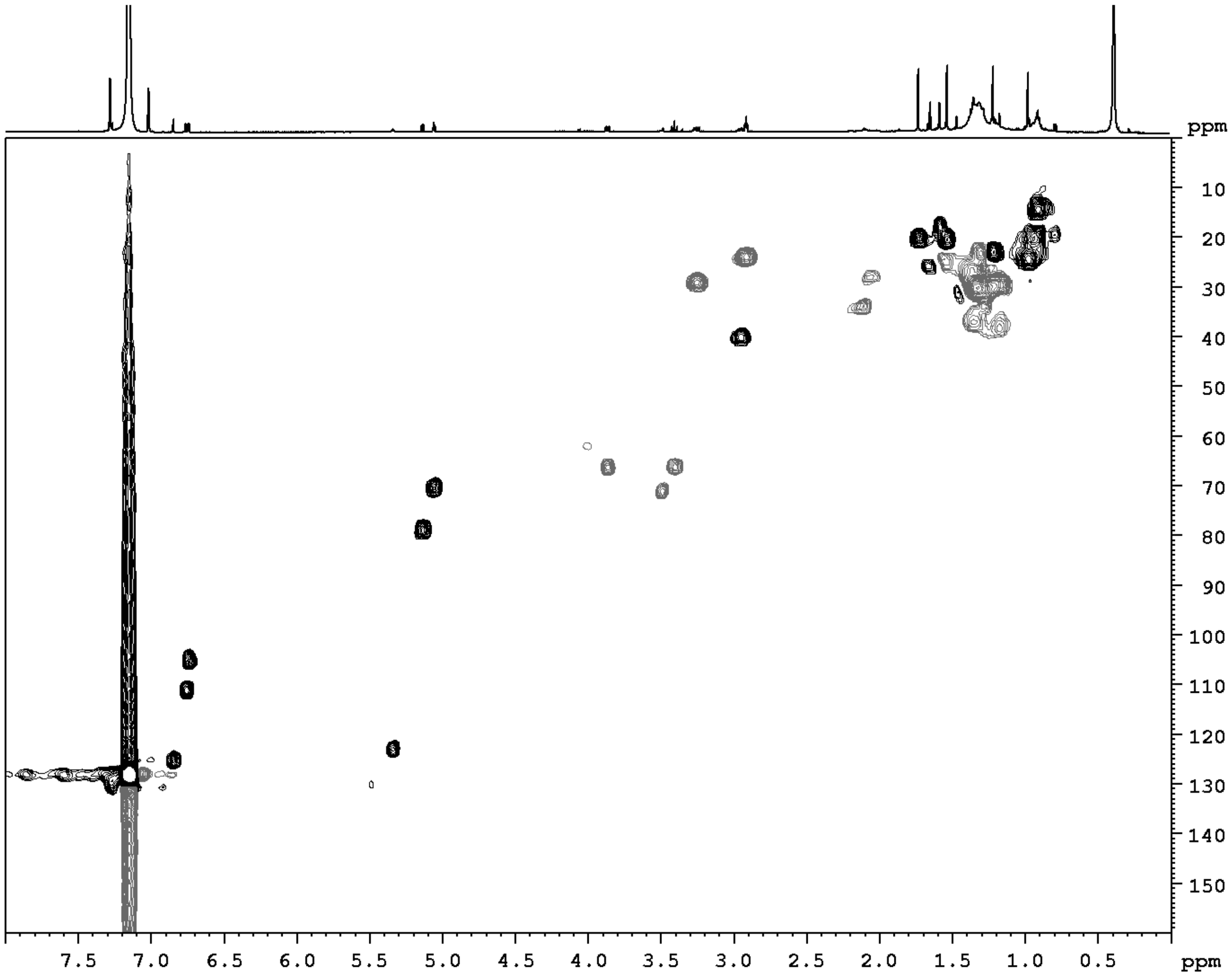

ed-HSQC spectrum of acetylated bituminarin A (**1a**) (600 MHz, C_6D_6)

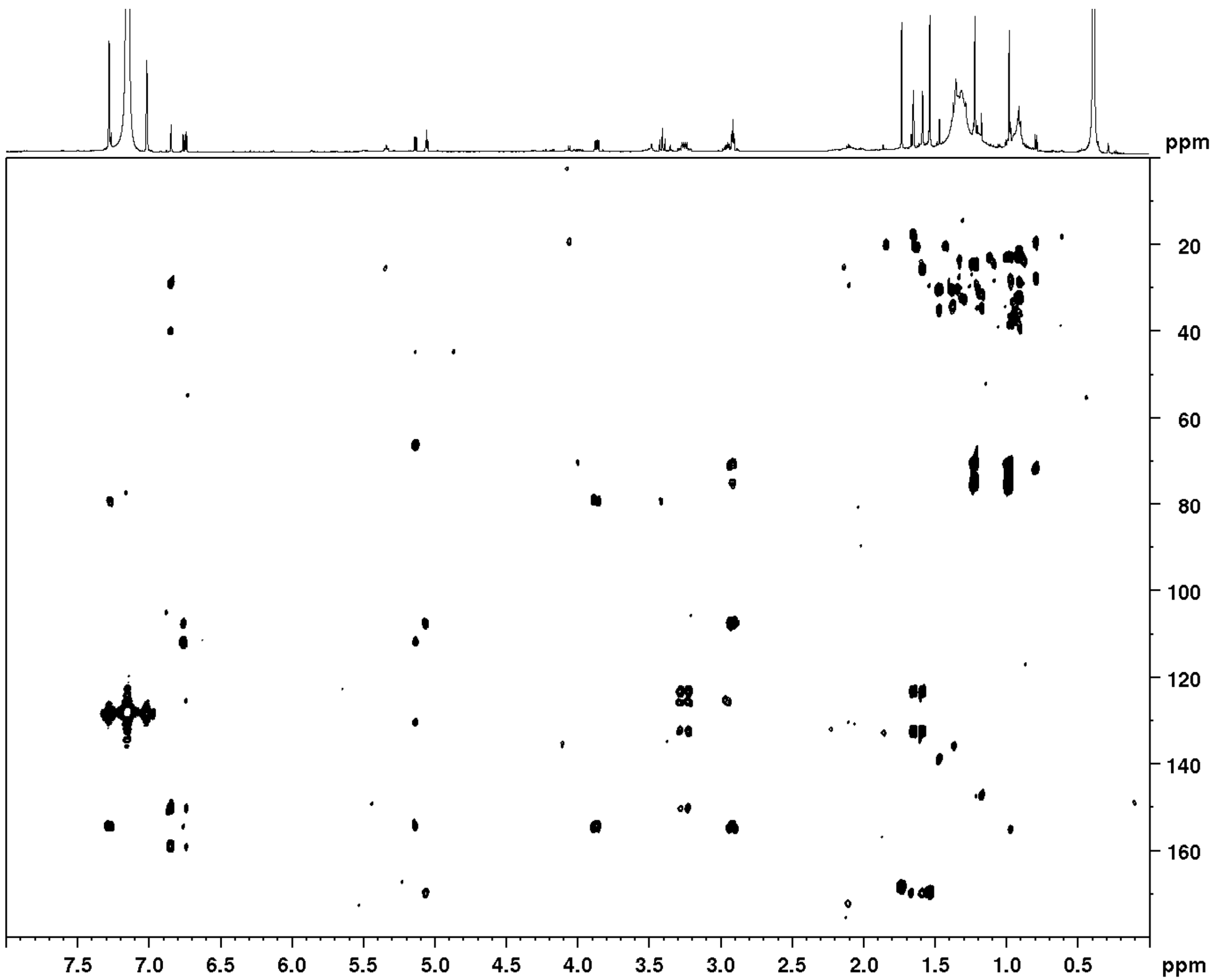

HMBC spectrum of acetylated bituminarin A (**1a**) (600 MHz, C_6D_6)

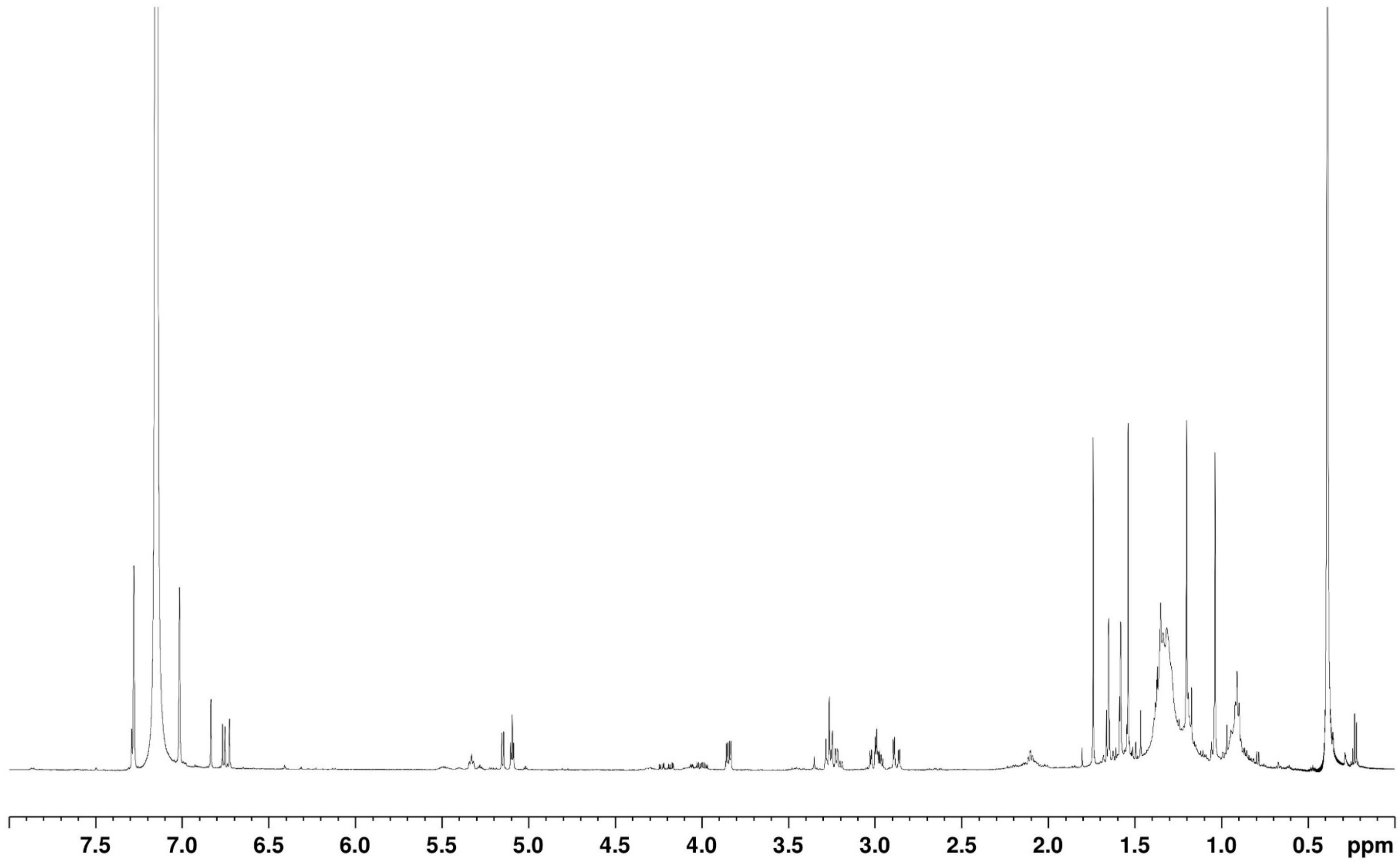

^{1}H NMR spectrum of acetylated bituminarin B (**2a**) (600 MHz, C_6D_6)

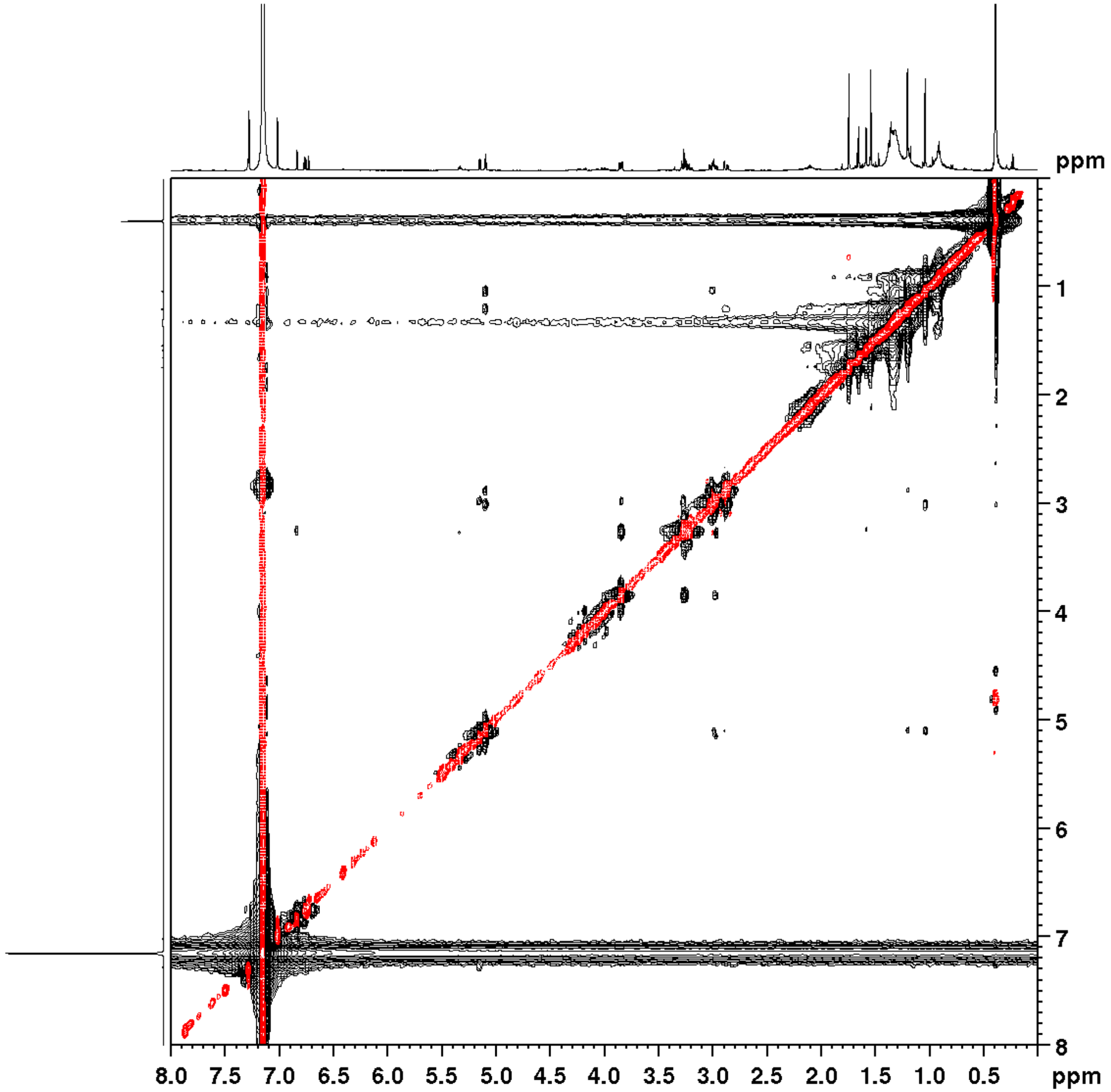

NOESY spectrum of acetylated bituminarin B (**2a**) (600 MHz, C_6D_6)

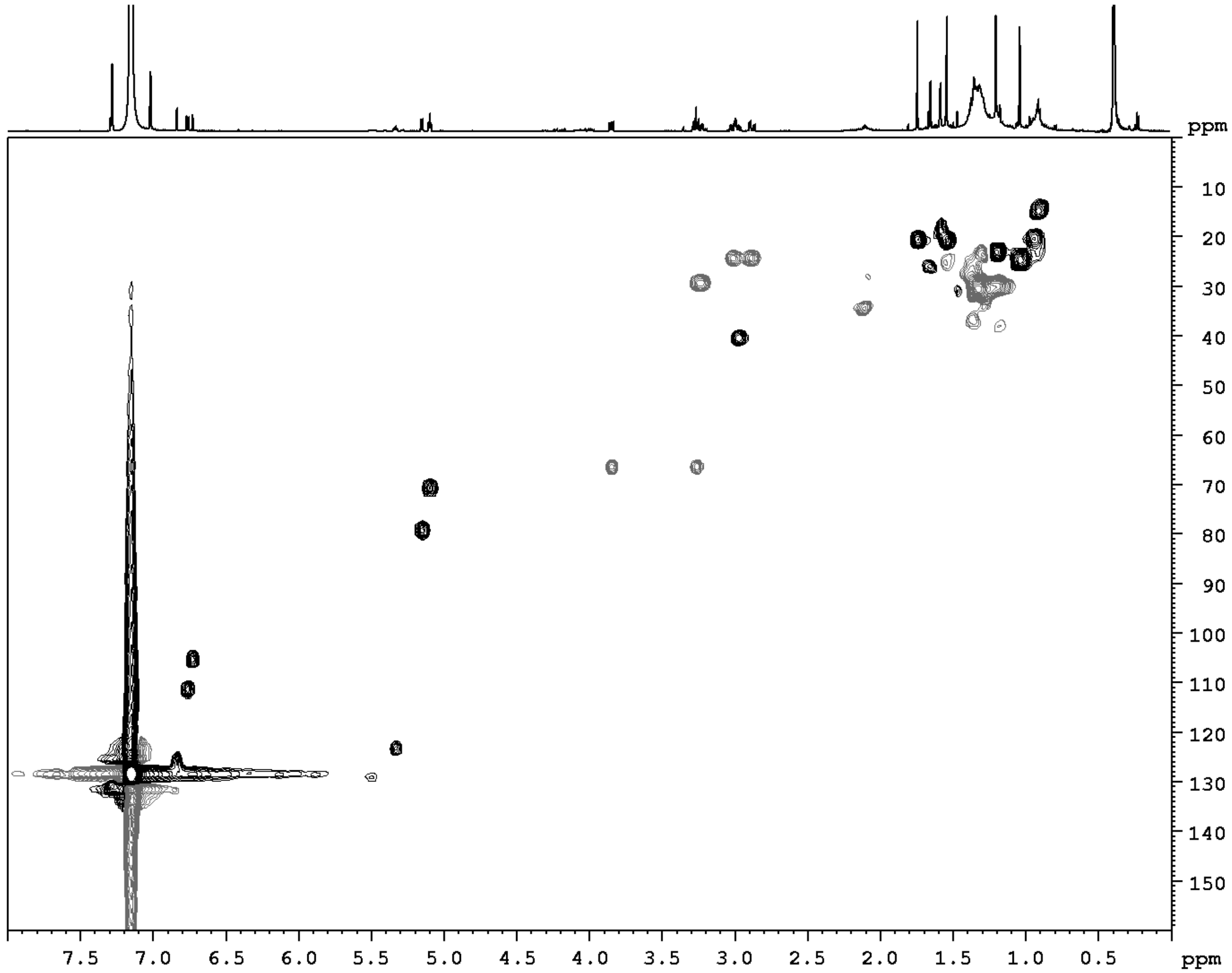

ed-HSQC spectrum of acetylated bituminarin B (**2a**) (600 MHz C_6D_6)

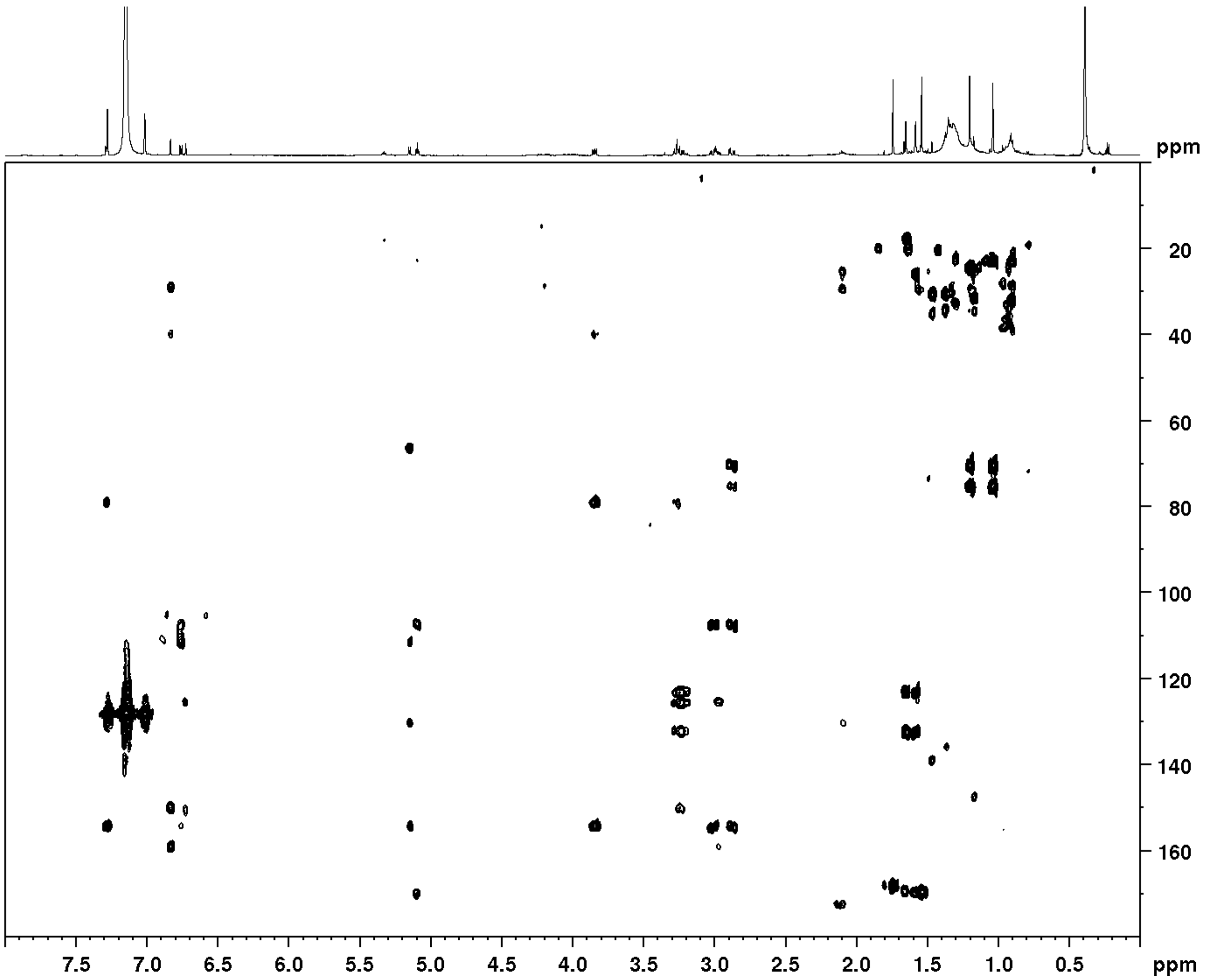

HMBC spectrum of acetylated bituminarin B (**2a**) (600 MHz, C_6D_6)

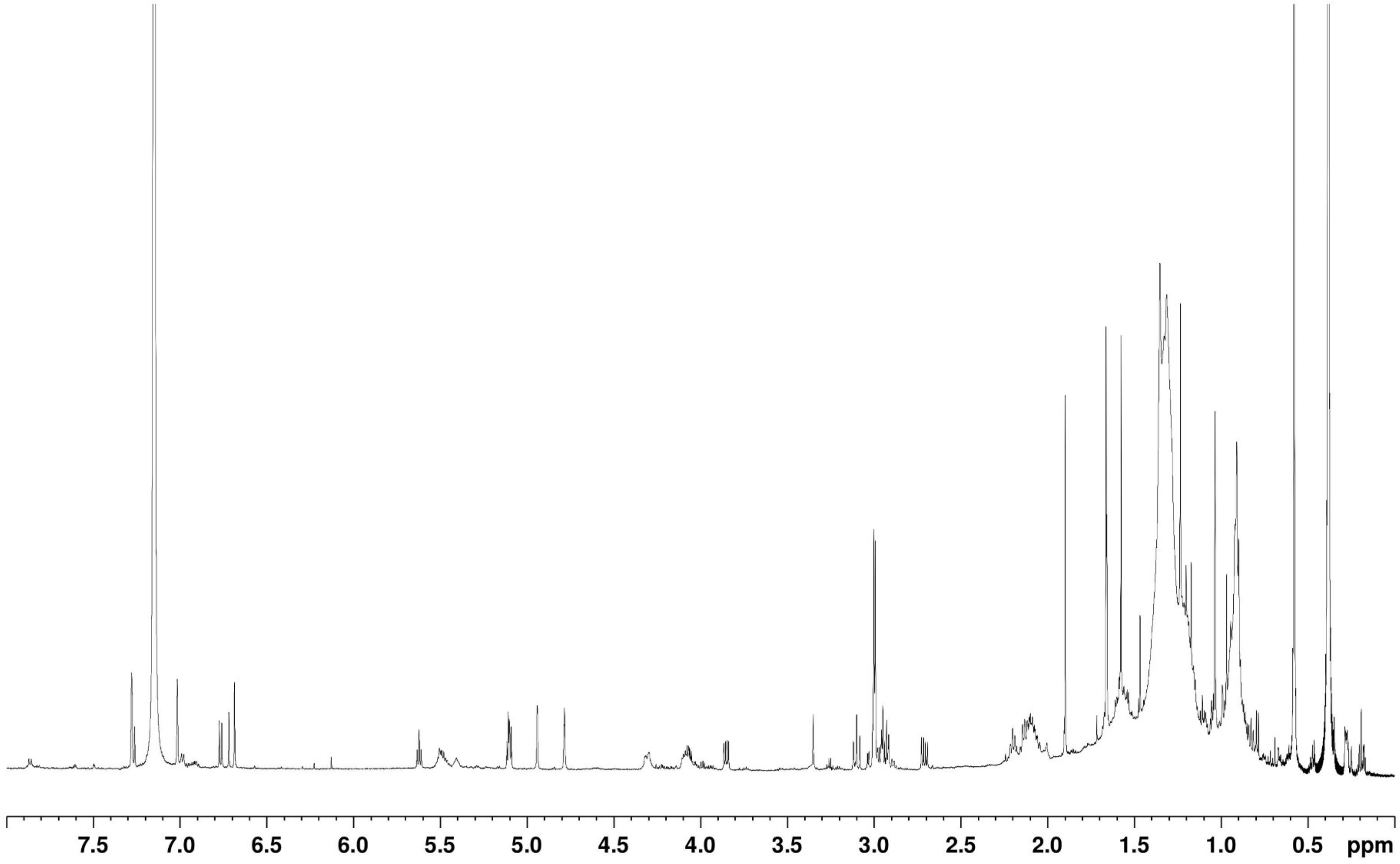

^{1}H NMR spectrum of acetylated bituminarin C (**3a**) (600 MHz, C_6D_6)

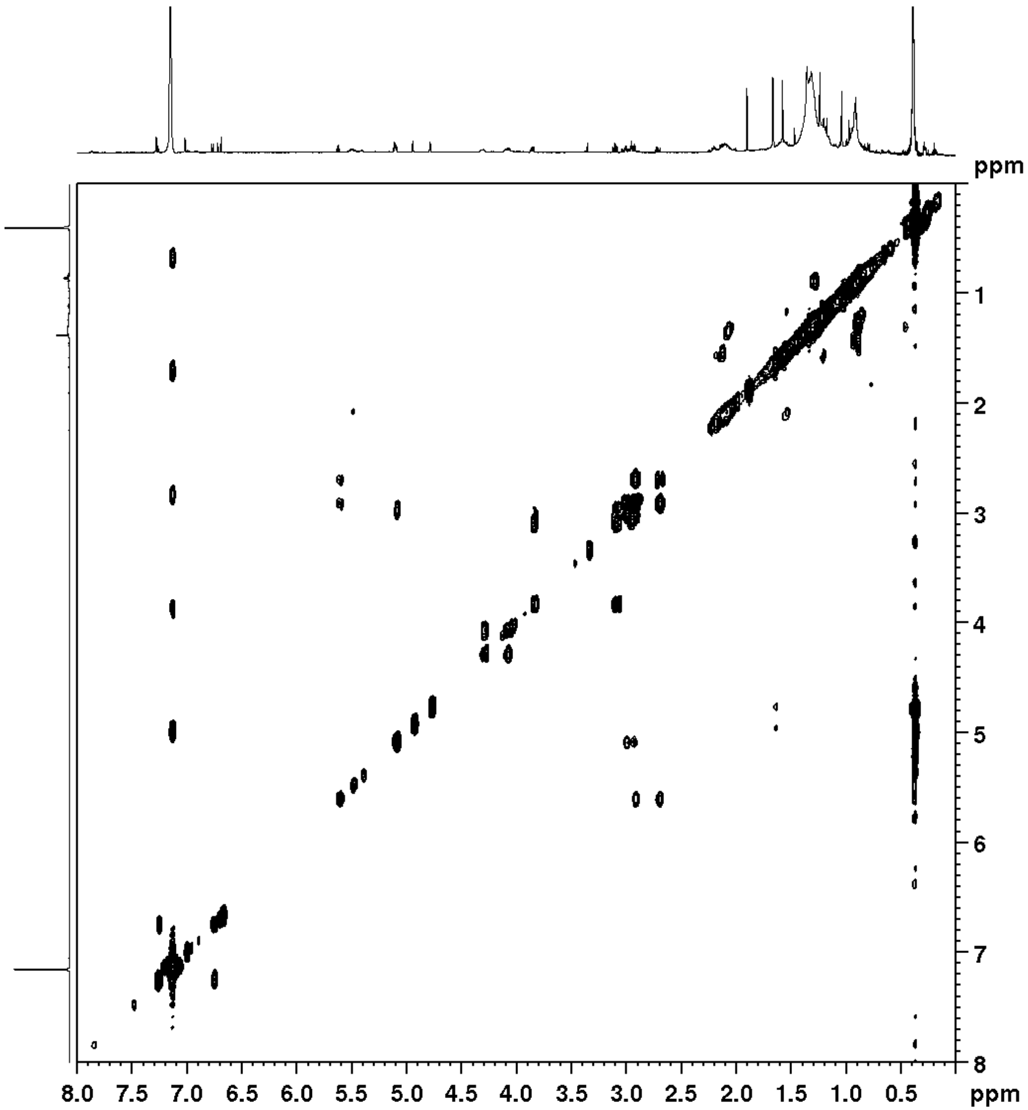

COSY spectrum of acetylated bituminarin C (**3a**) (400 MHz, C_6D_6)

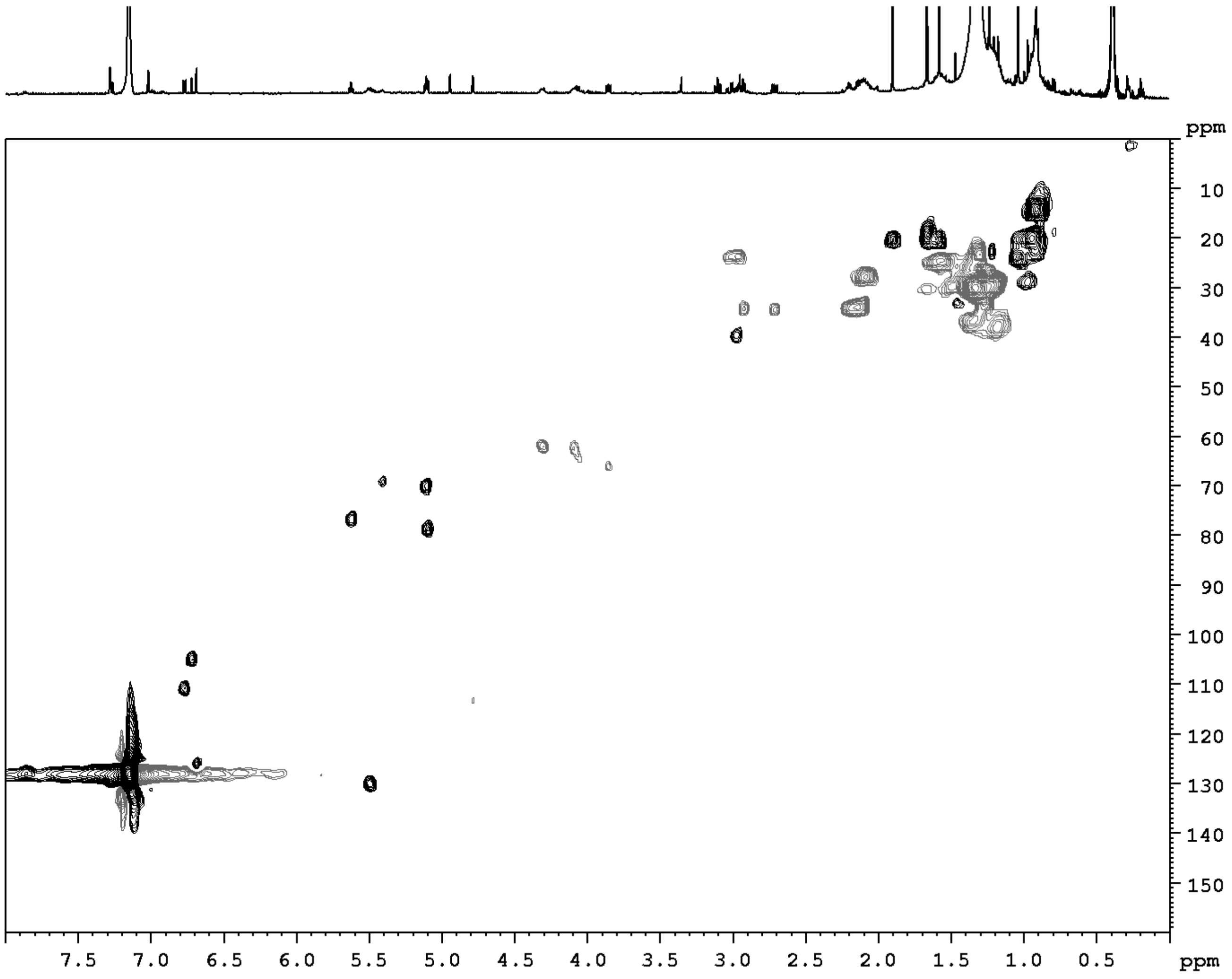

ed-HSQC spectrum of acetylated bituminarin C (**3a**) (400 MHz, C_6D_6)

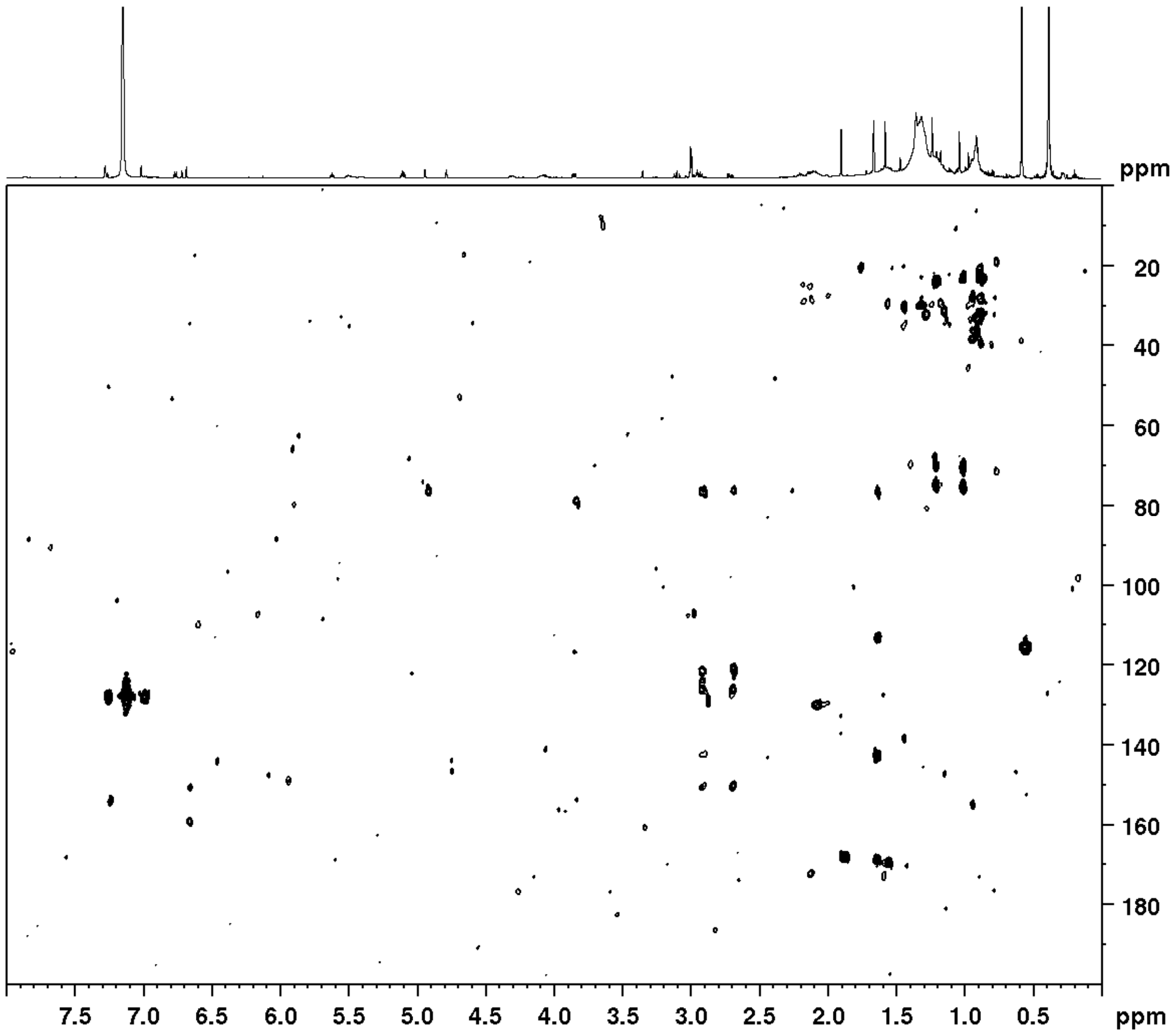

HMBC spectrum of acetylated bituminarin C (**3a**) (600 MHz, C_6D_6)

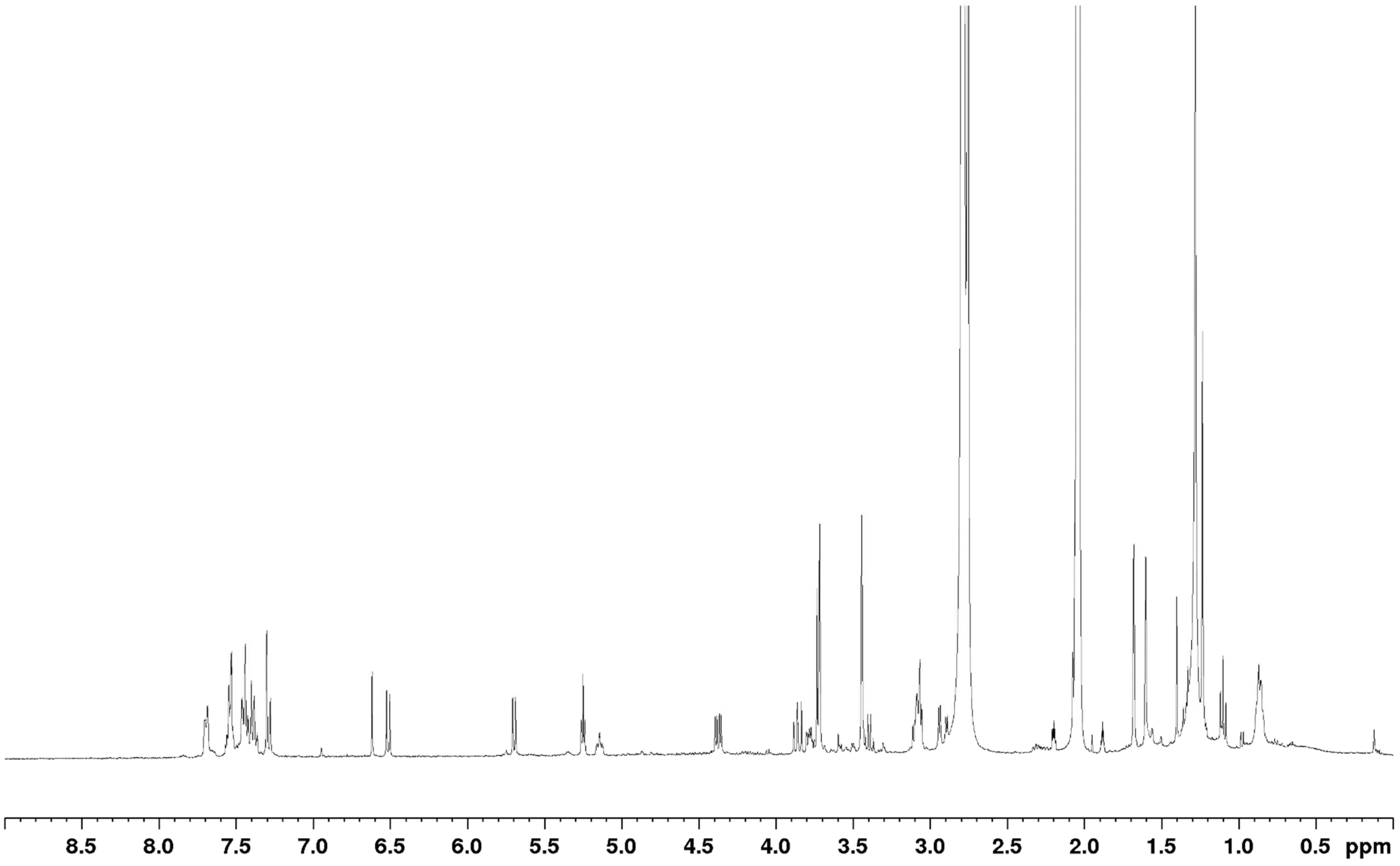

^{1}H NMR spectrum of (*S*)-MTPA-ester of bituminarin A (**1b**) (600 MHz, Acetone-*d*6)

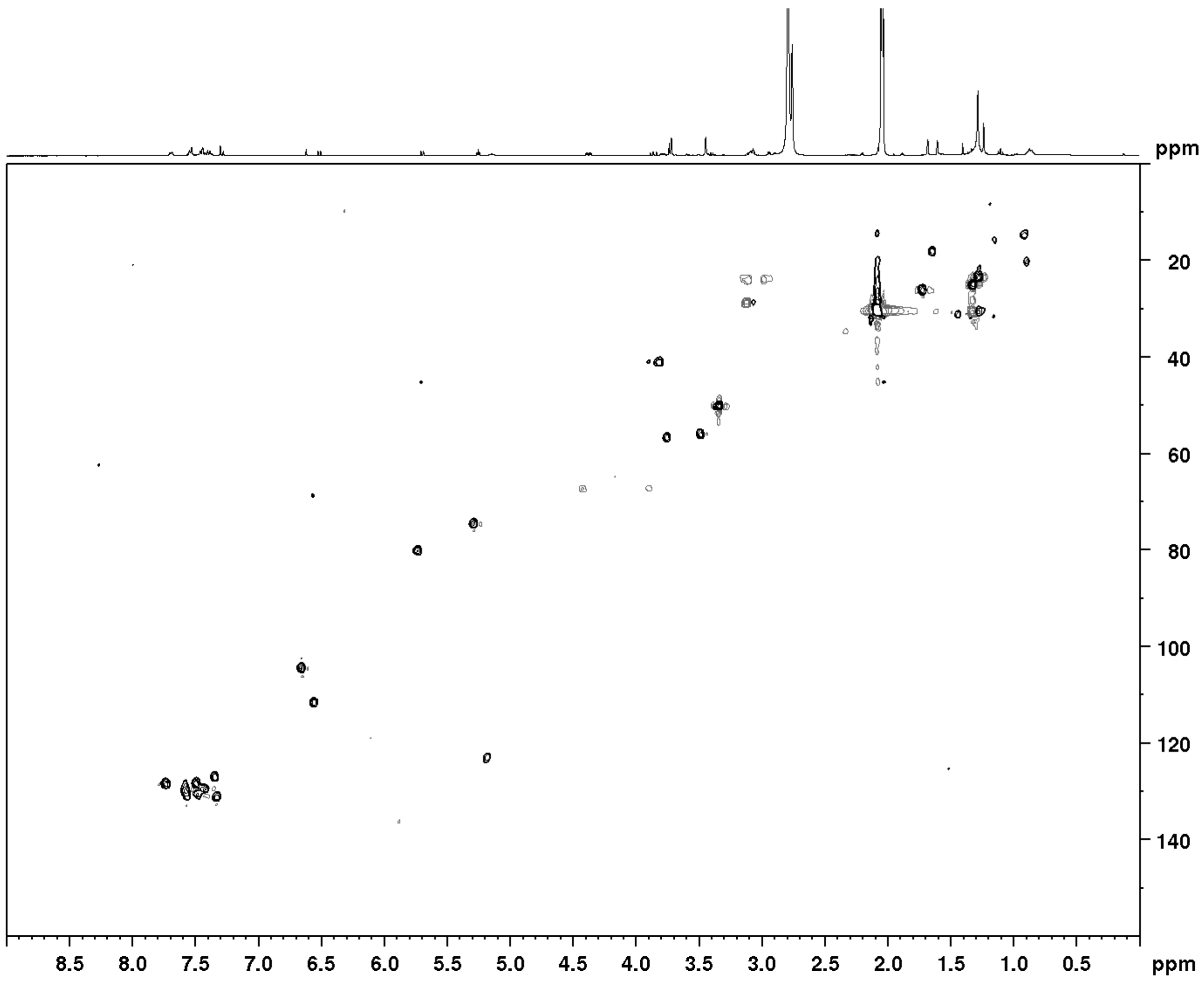

ed-HSQC spectrum of (*S*)-MTPA-ester of bituminarin A (**1b**) (600 MHz, Acetone-*d6*)

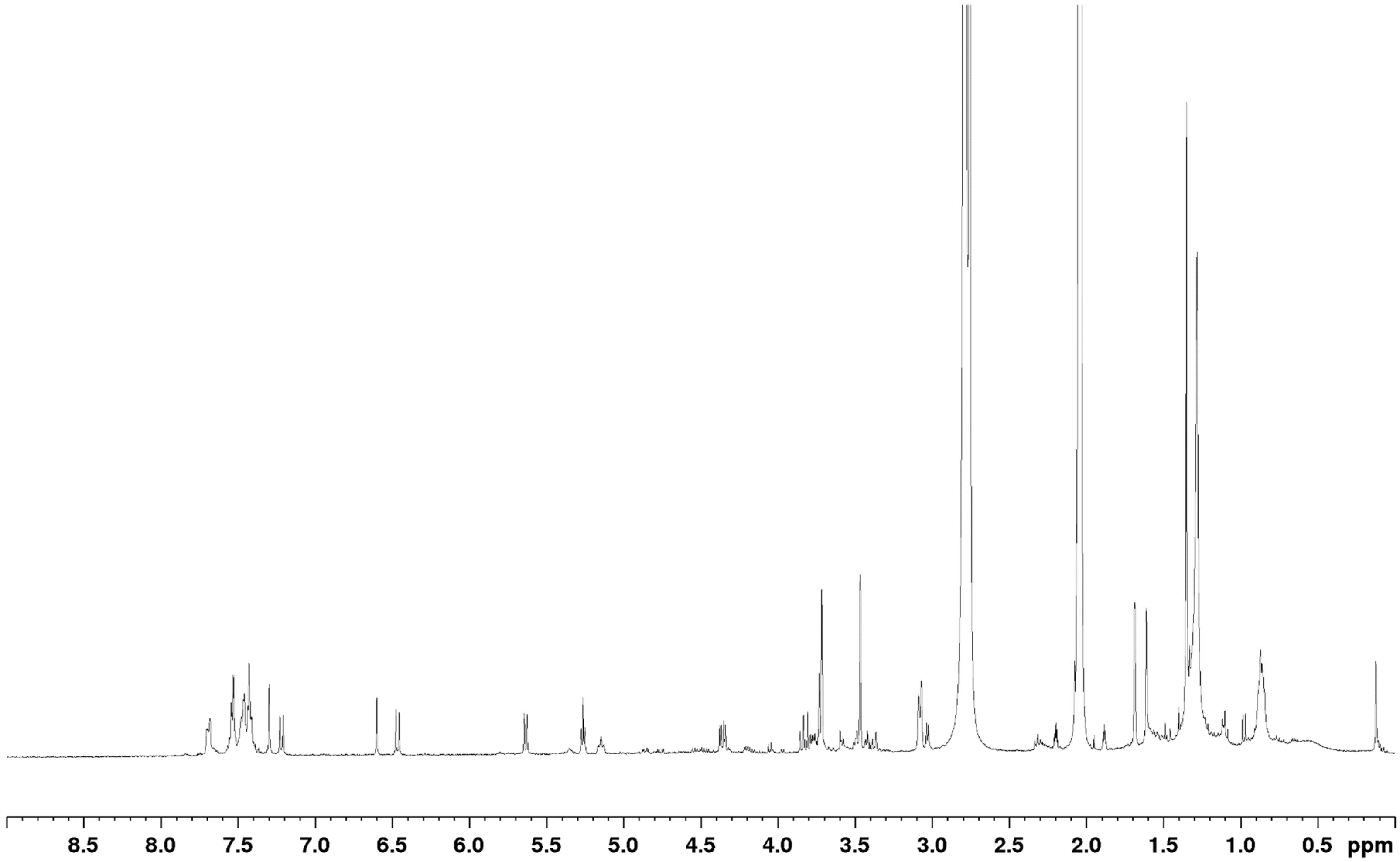

^{1}H NMR spectrum of (*R*)-MTPA-ester of bituminarin A (**1c**) (600 MHz, Acetone-*d6*)

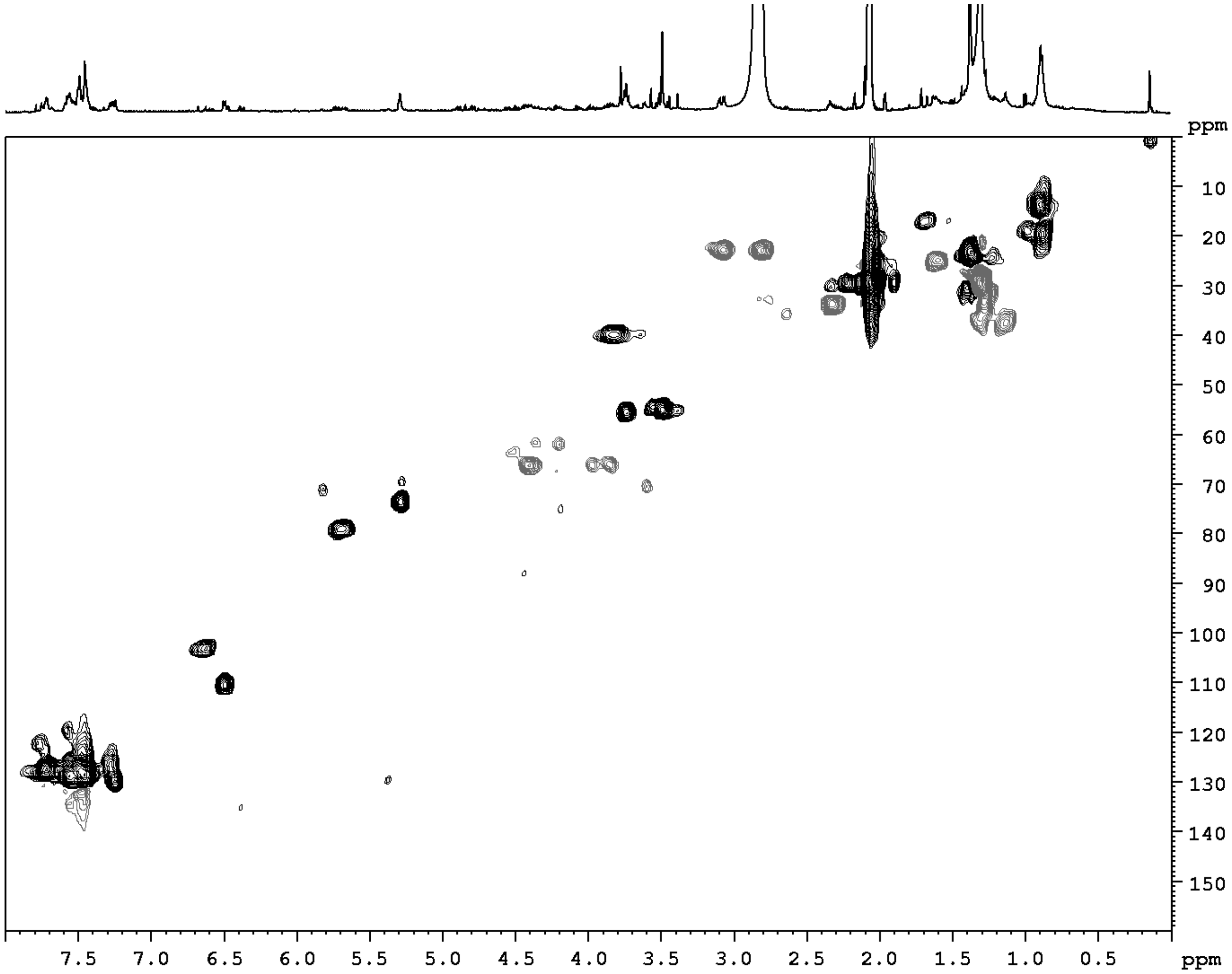

ed-HSQC spectrum of (*R*)-MTPA-ester of bituminarin A (**1c**) (600 MHz, Acetone-*d6*)

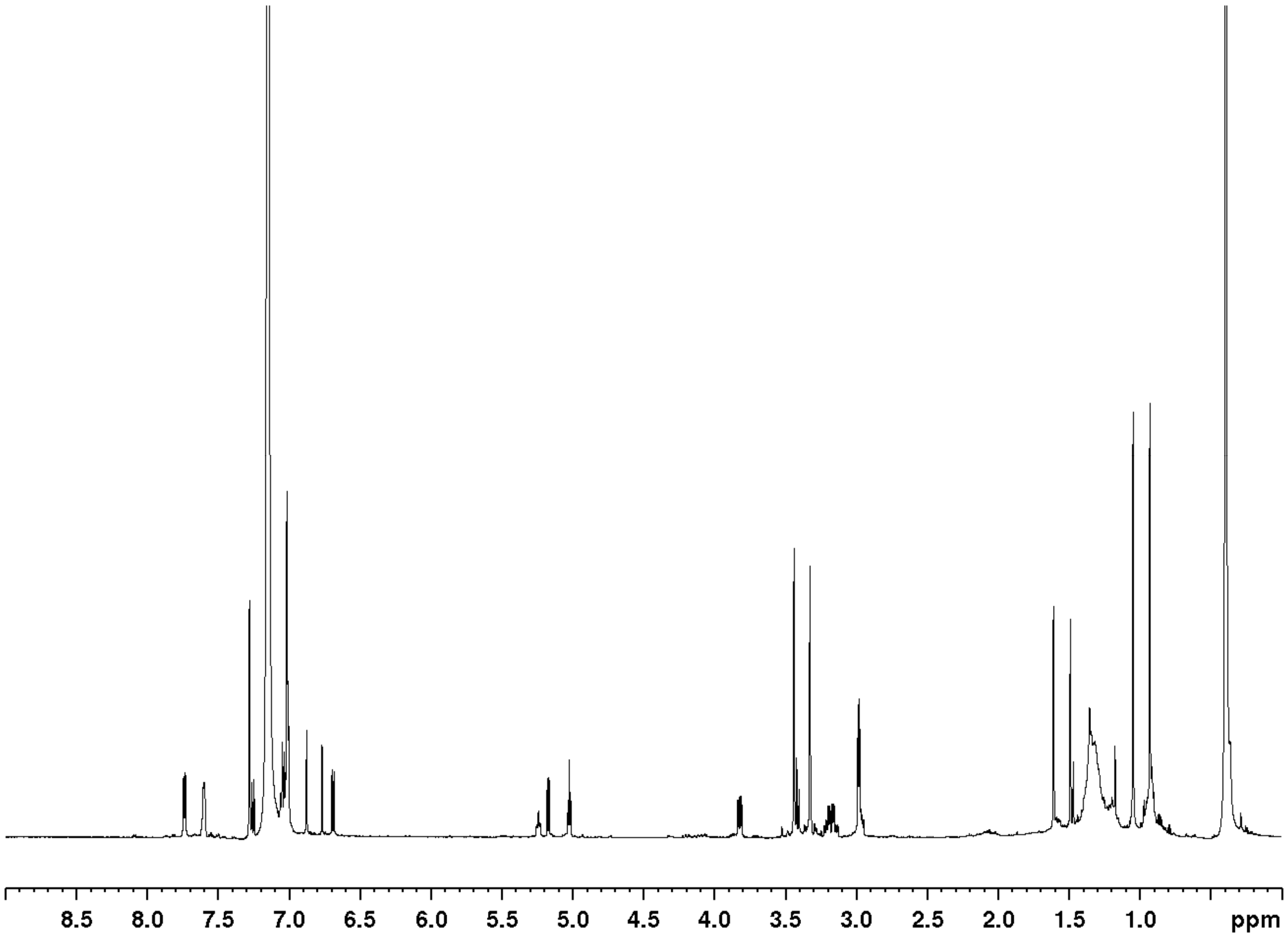

^{1}H NMR spectrum of (*S*)-MTPA-ester of bituminarin A (**1b**) (600 MHz, C_6D_6)

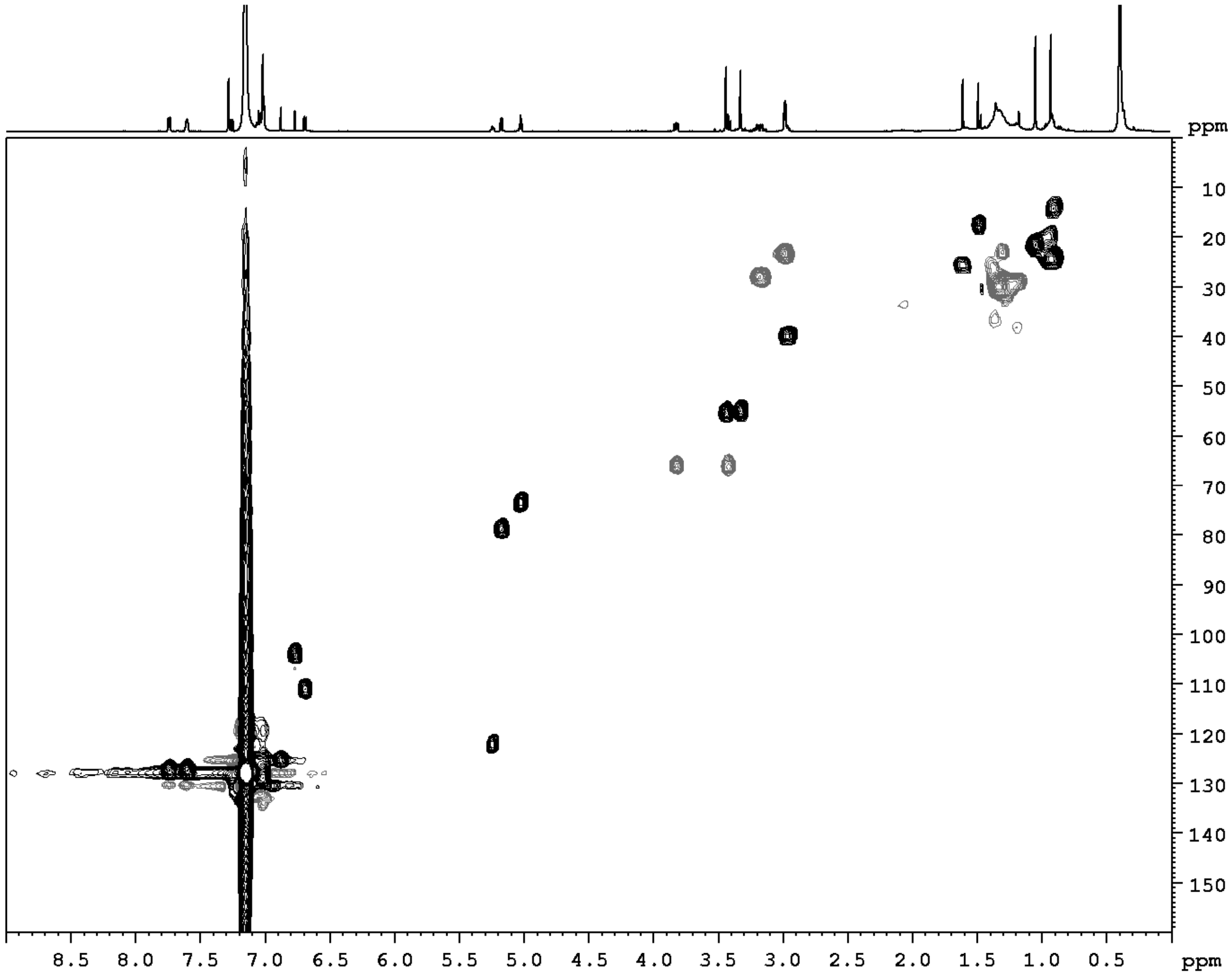

ed-HSQC spectrum of (*S*)-MTPA-ester of bituminarin A (**1b**) (600 MHz, C_6D_6)

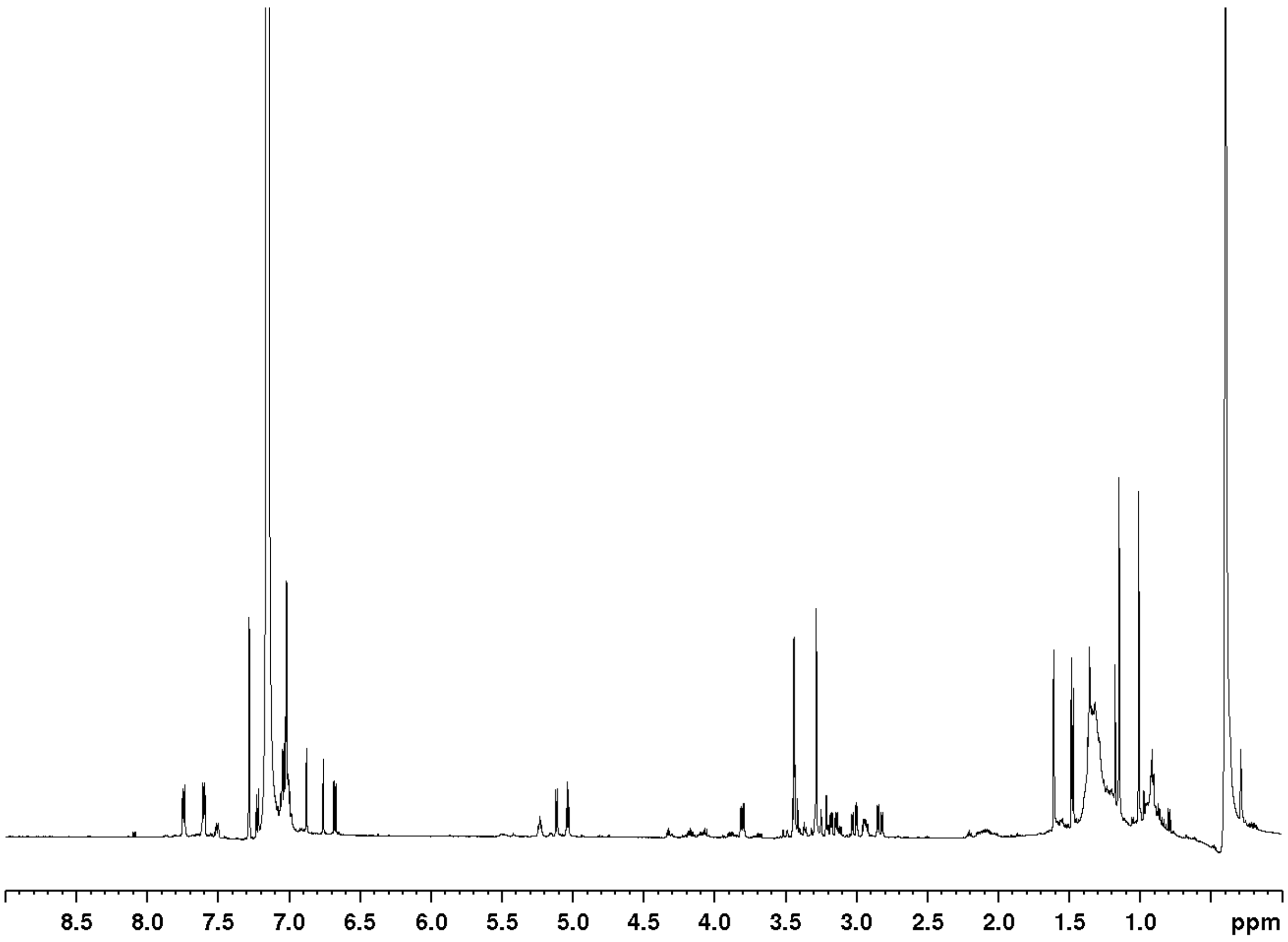

^{1}H NMR spectrum of (*R*)-MTPA-ester of bituminarin A (**1c**) (600 MHz, C_6D_6)

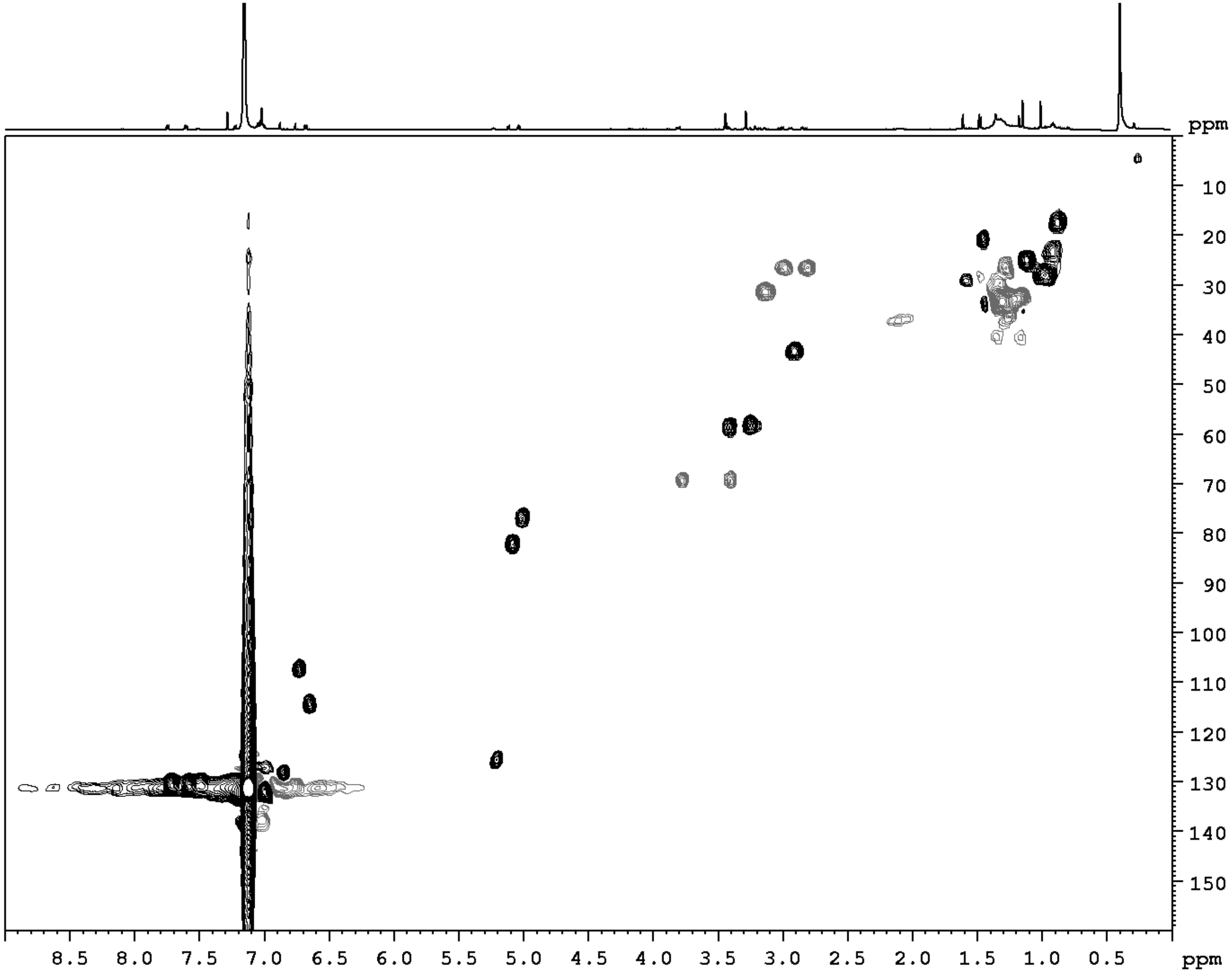

ed-HSQC spectrum of (*R*)-MTPA-ester of bituminarin A (**1c**) (600 MHz, C_6D_6)

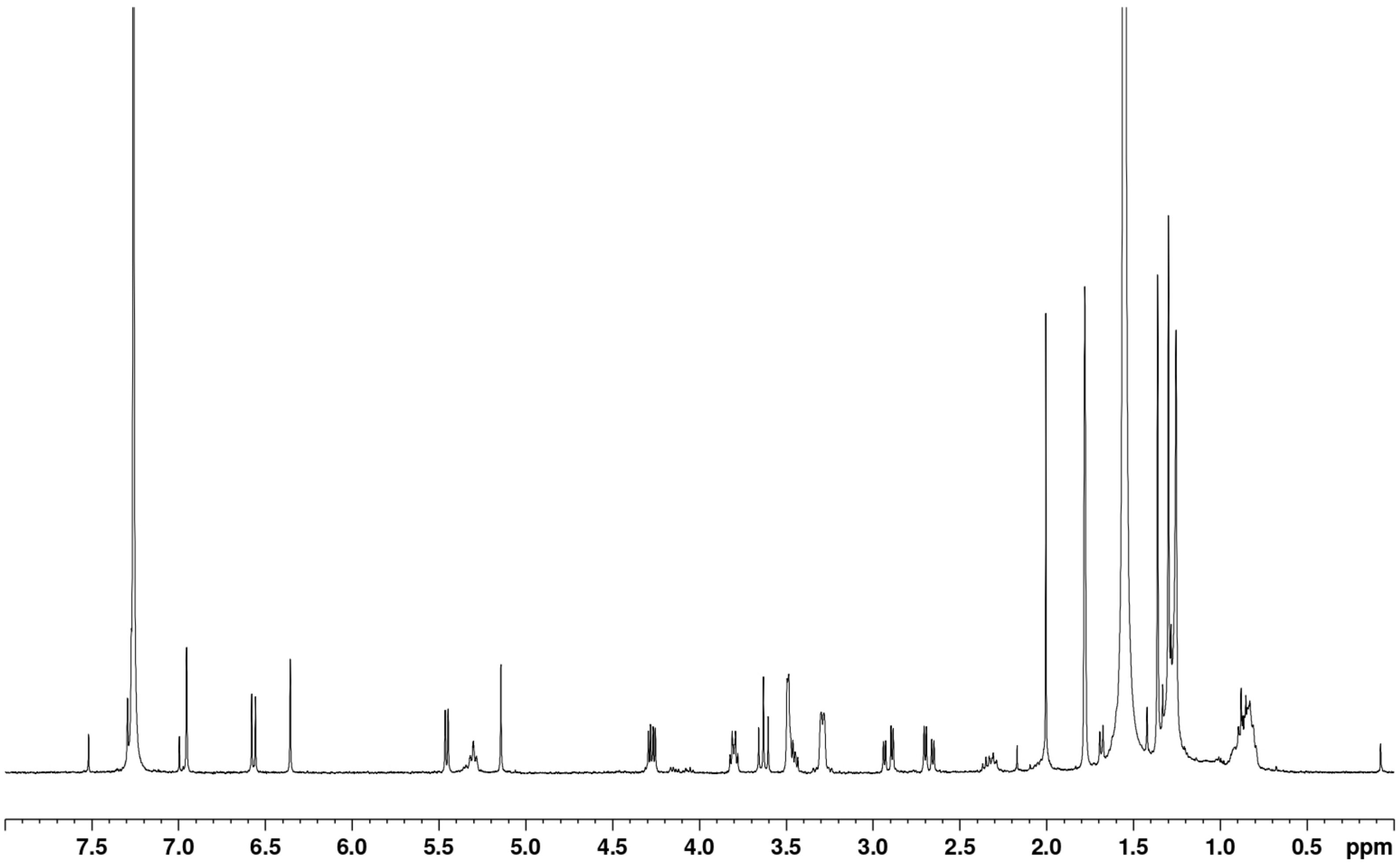

^{1}H NMR spectrum of bituminarin A (**1**) (400 MHz, $CDCl_3$)

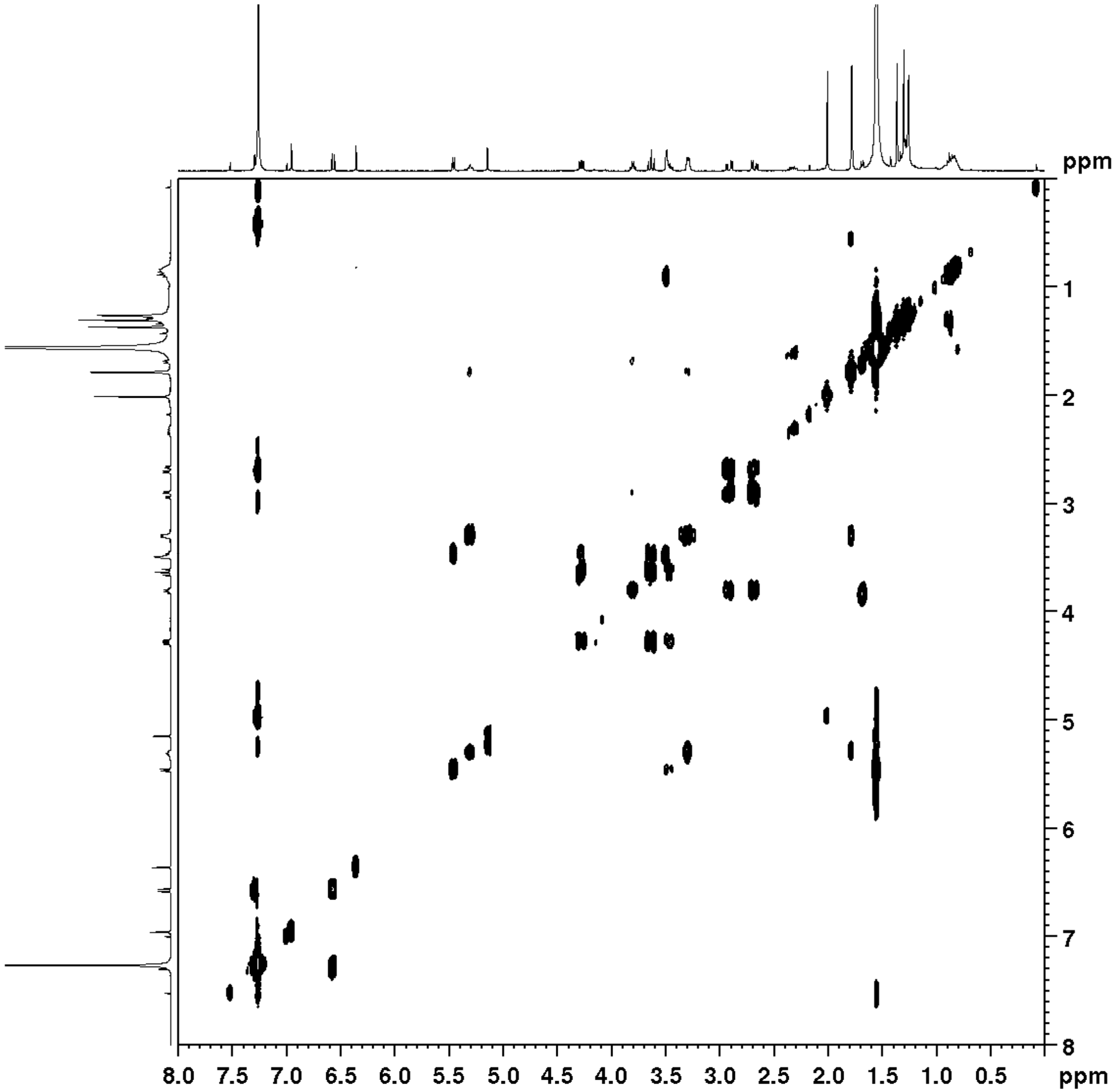

COSY spectrum of bituminarin A (**1**) (400 MHz, $CDCl_3$)

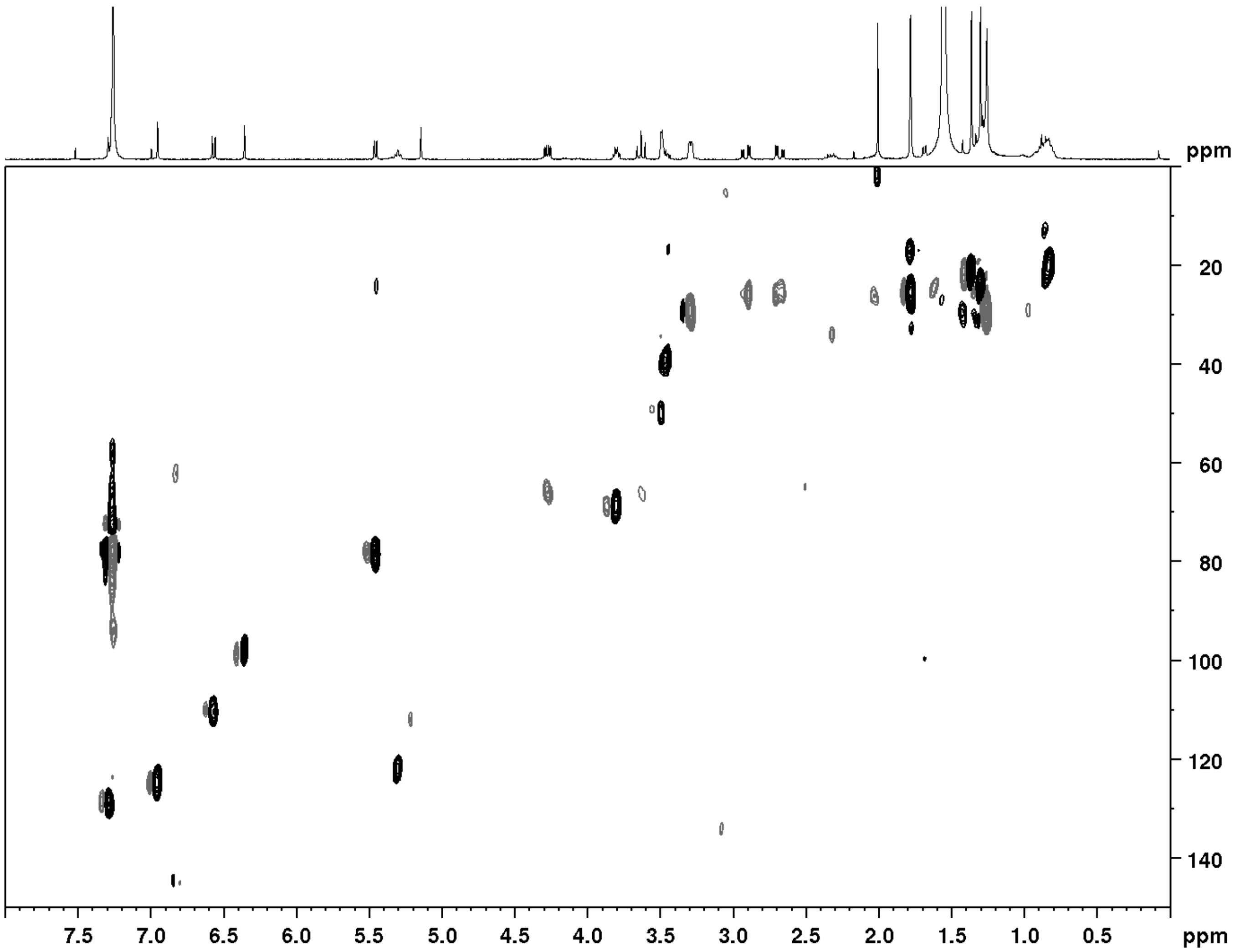

ed-HSQC spectrum of bituminarin A (**1**) (400 MHz, $CDCl_3$)

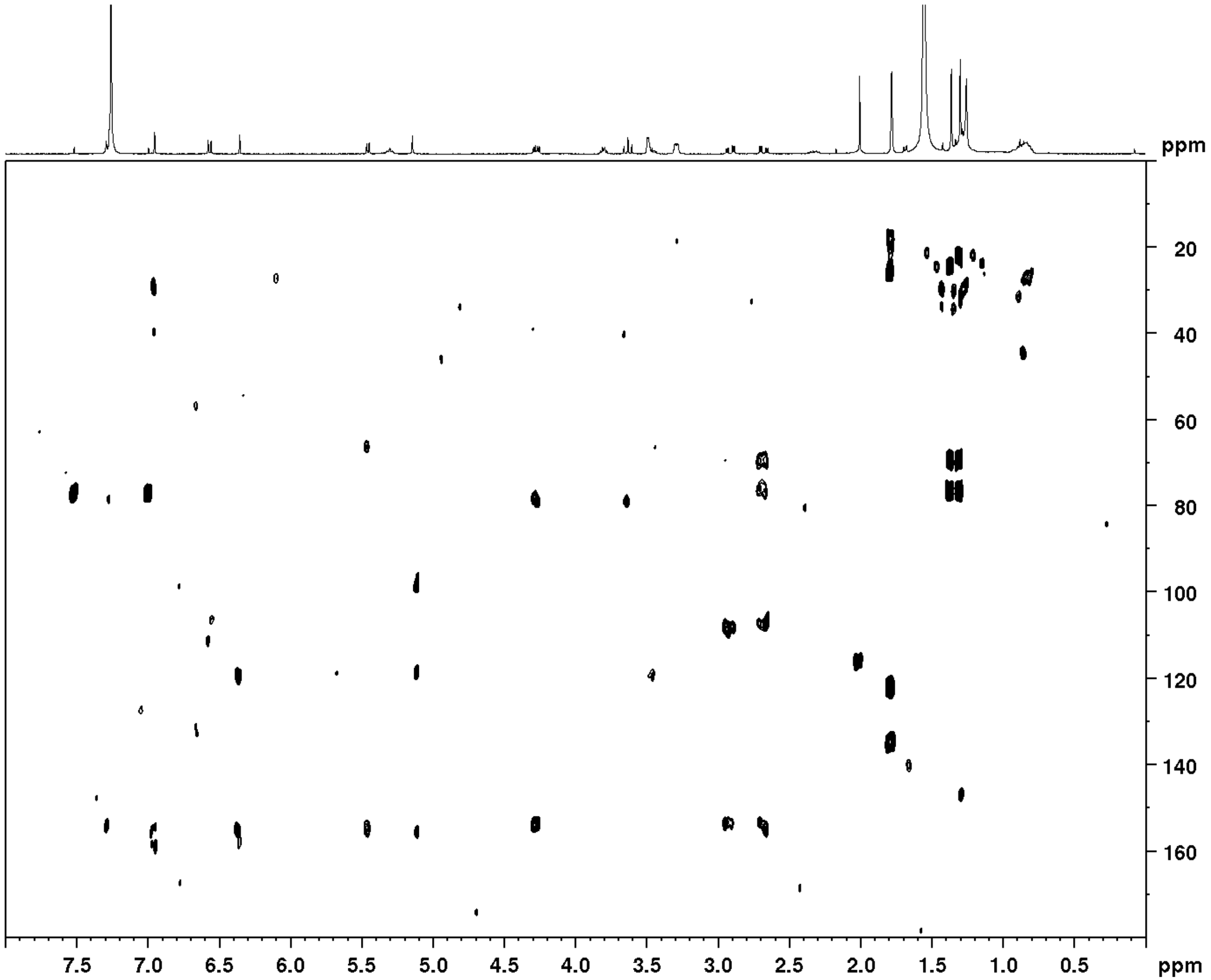

HMBC spectrum of bituminarin A (**1**) (400 MHz, $CDCl_3$)

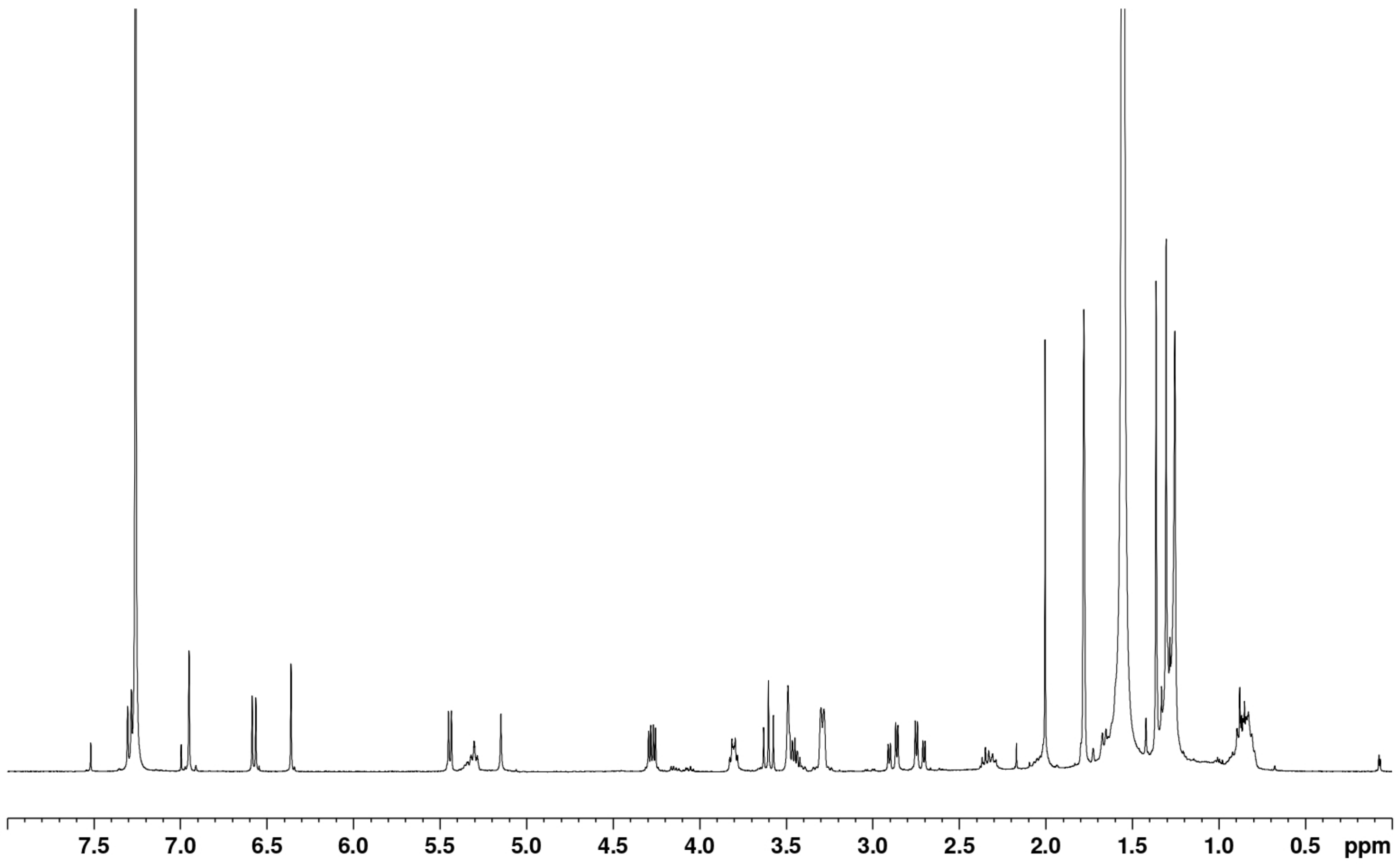

^{1}H NMR spectrum of bituminarin B (**2**) (400 MHz, $CDCl_3$)

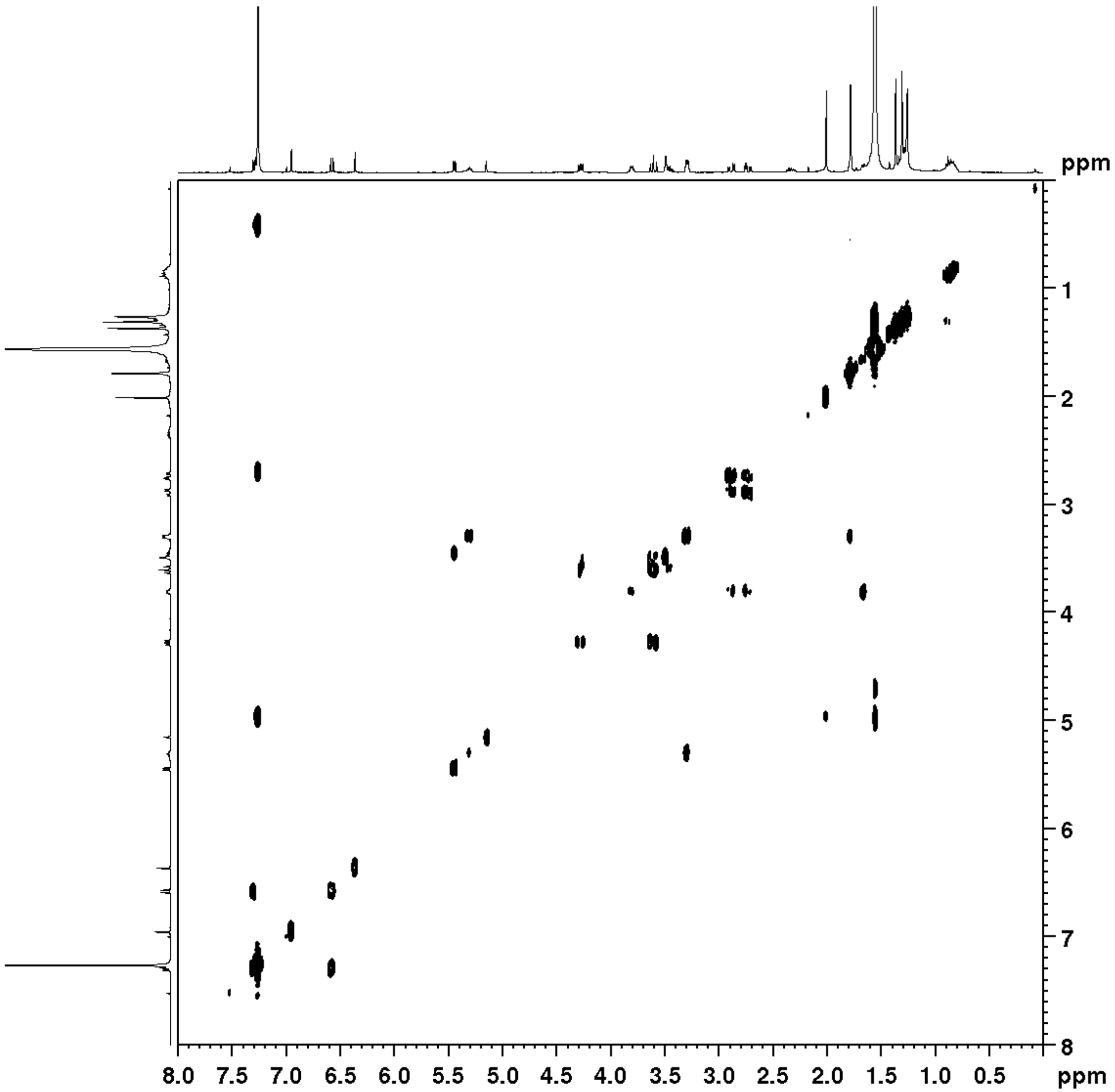

COSY spectrum of bituminarin B (**2**) (400 MHz, $CDCl_3$)

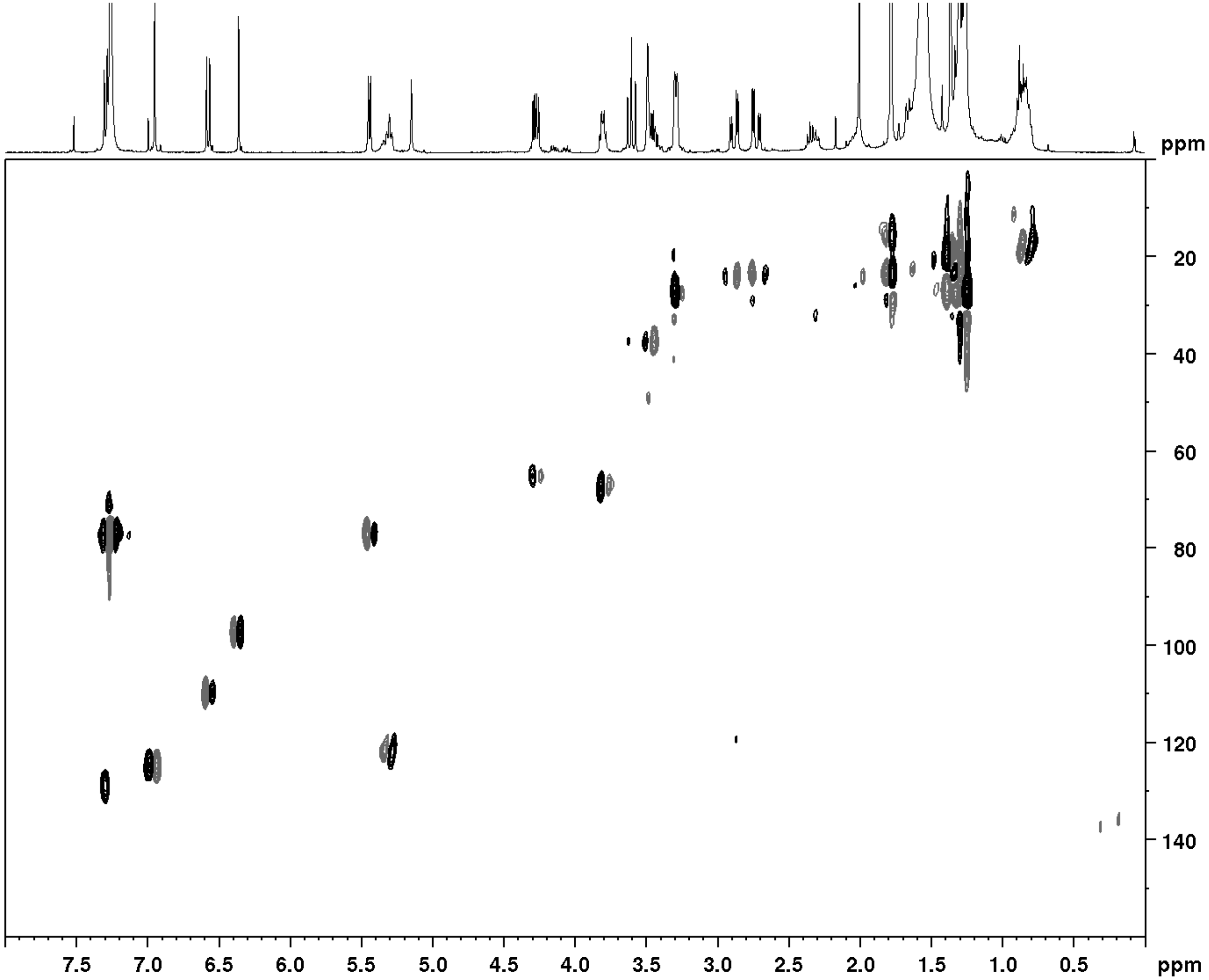

ed-HSQC spectrum of bituminarin B (**2**) (400 MHz, $CDCl_3$)

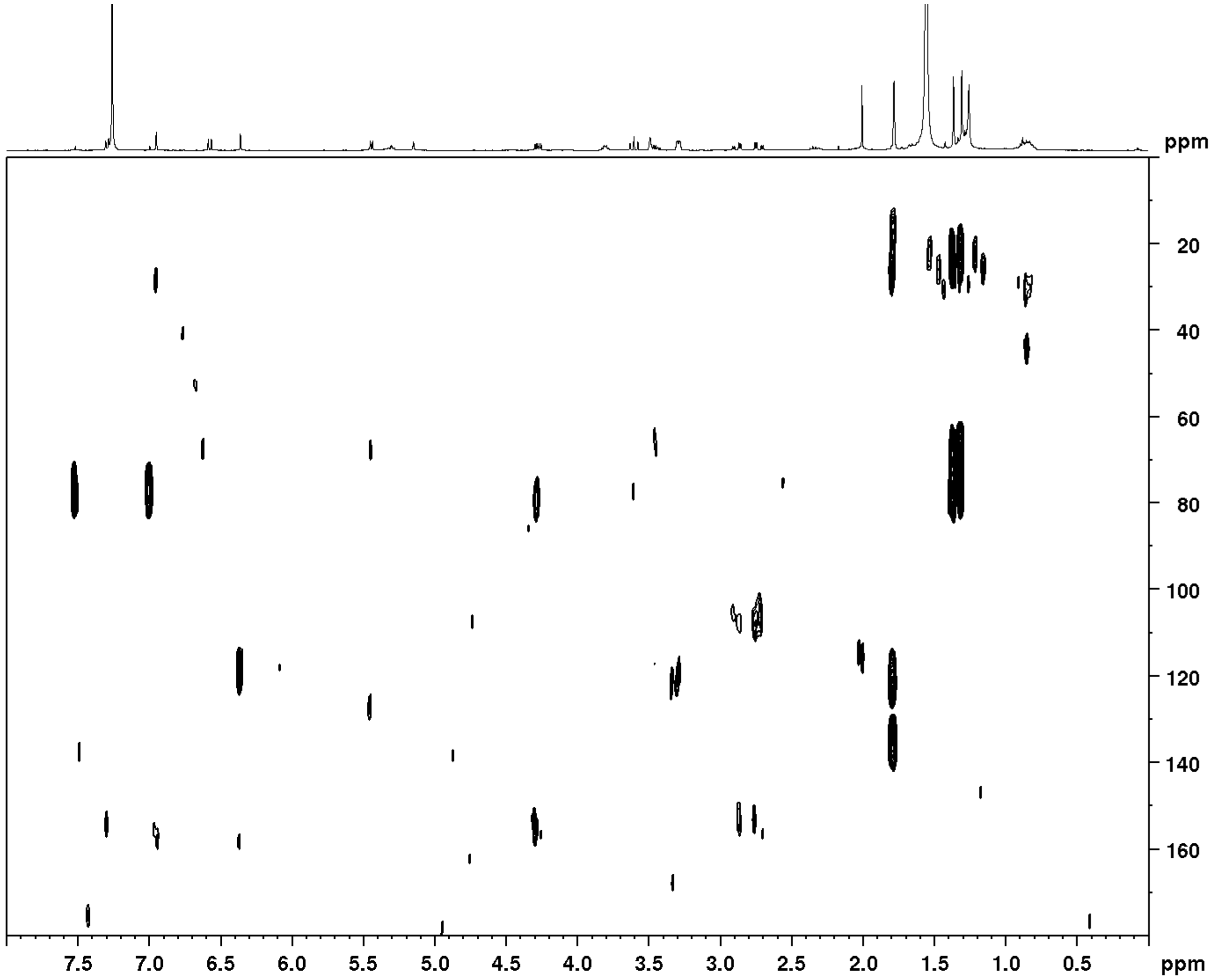

HMBC spectrum of bituminarin B (**2**) (400 MHz, $CDCl_3$)

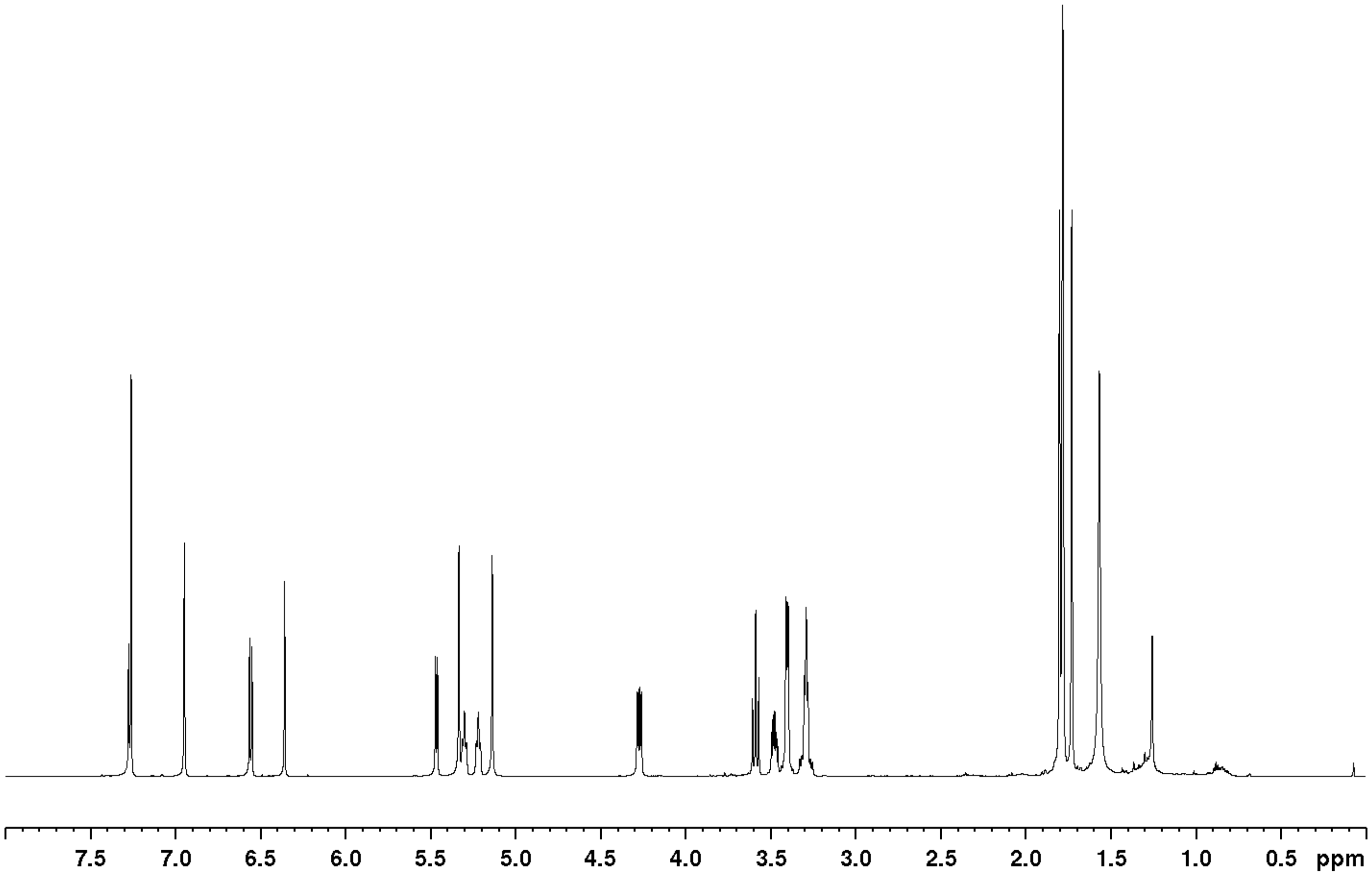

^{1}H NMR spectrum of erybraedin C (**5**) (600 MHz, $CDCl_3$)

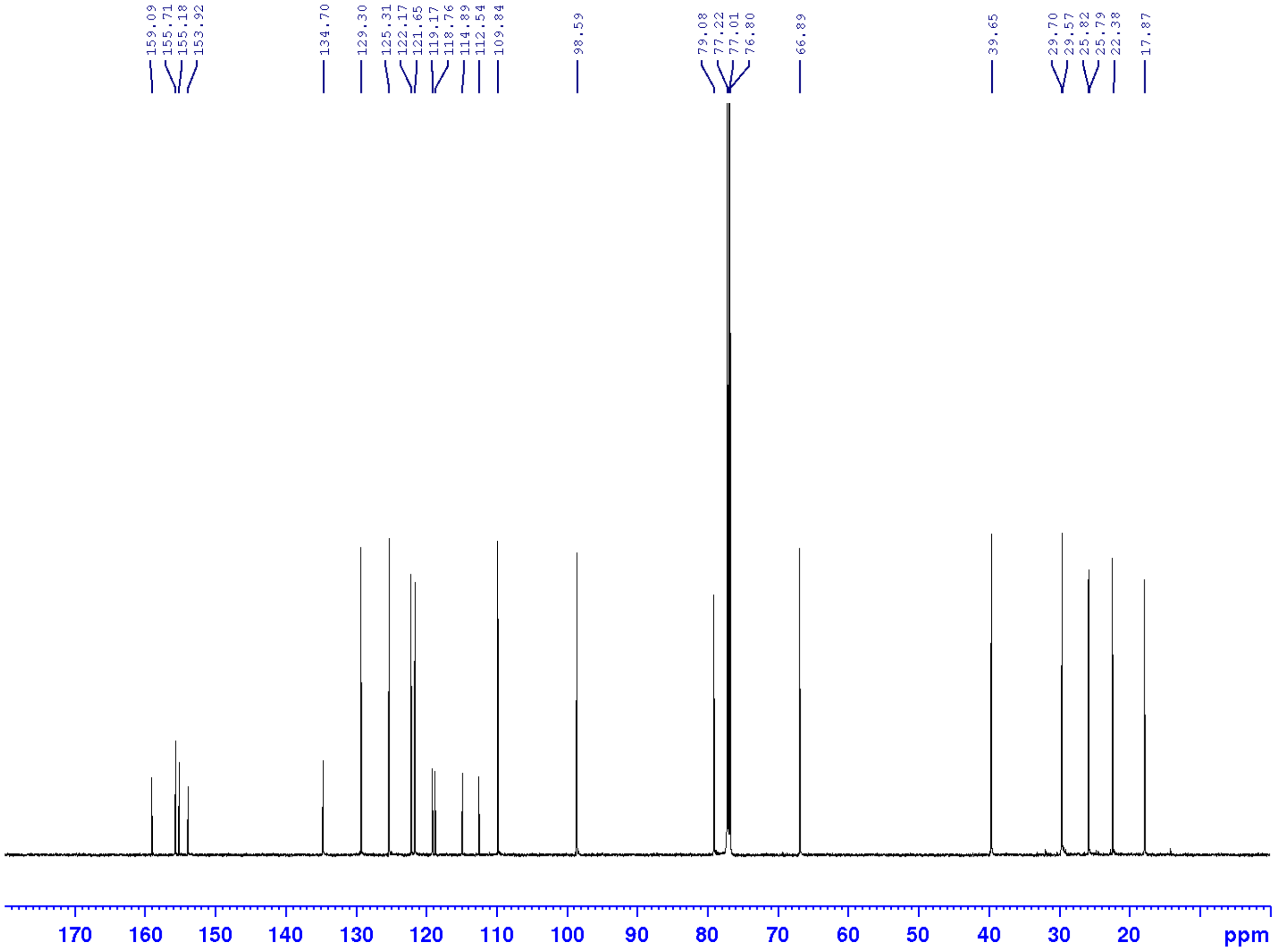

^{13}C NMR spectrum of erybraedin C (**5**) (150 MHz, $CDCl_3$)

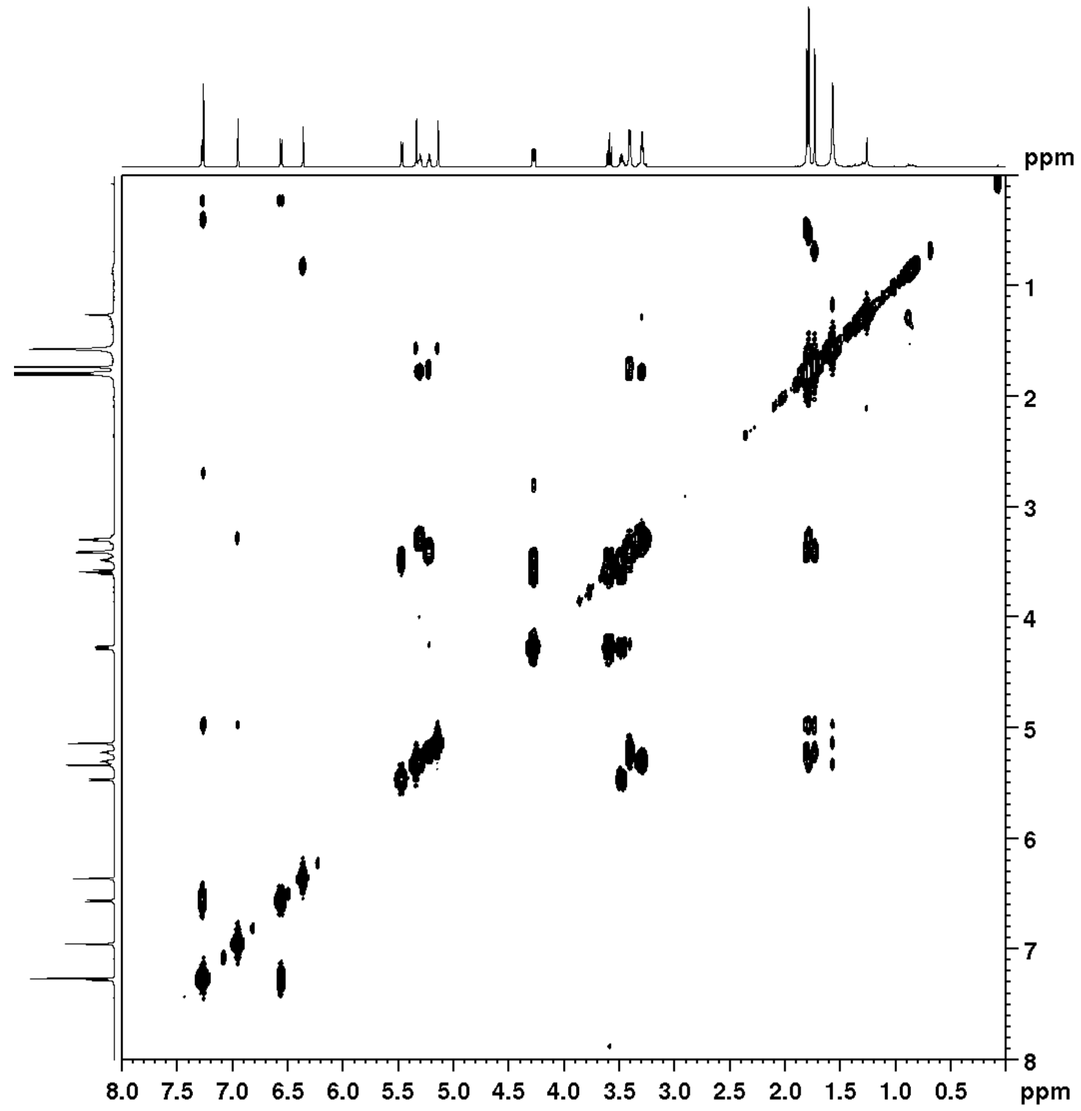

COSY spectrum of erybraedin C (**5**) (600 MHz, $CDCl_3$)

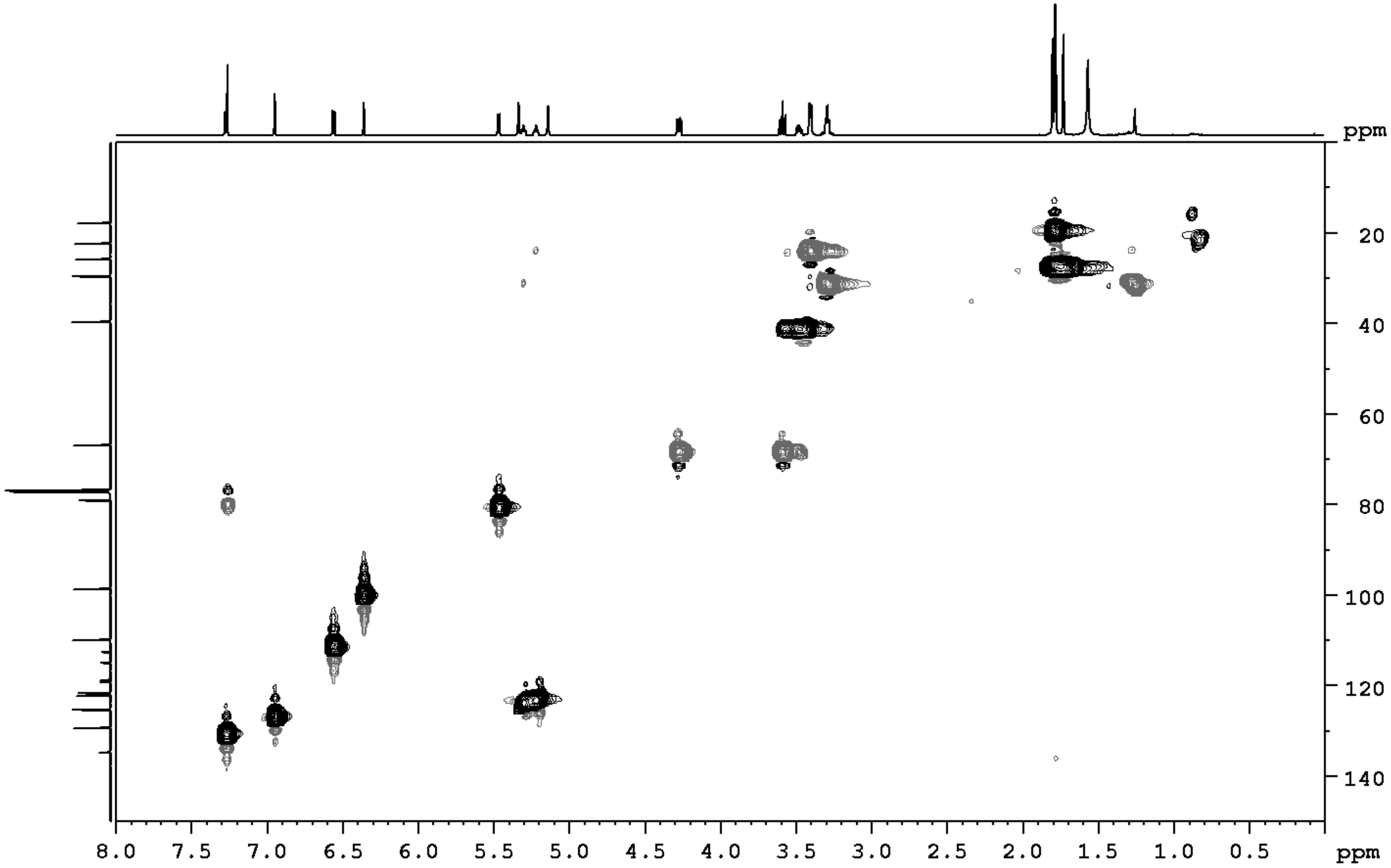

ed-HSQC spectrum of erybraedin C (**5**) (600 MHz, $CDCl_3$)

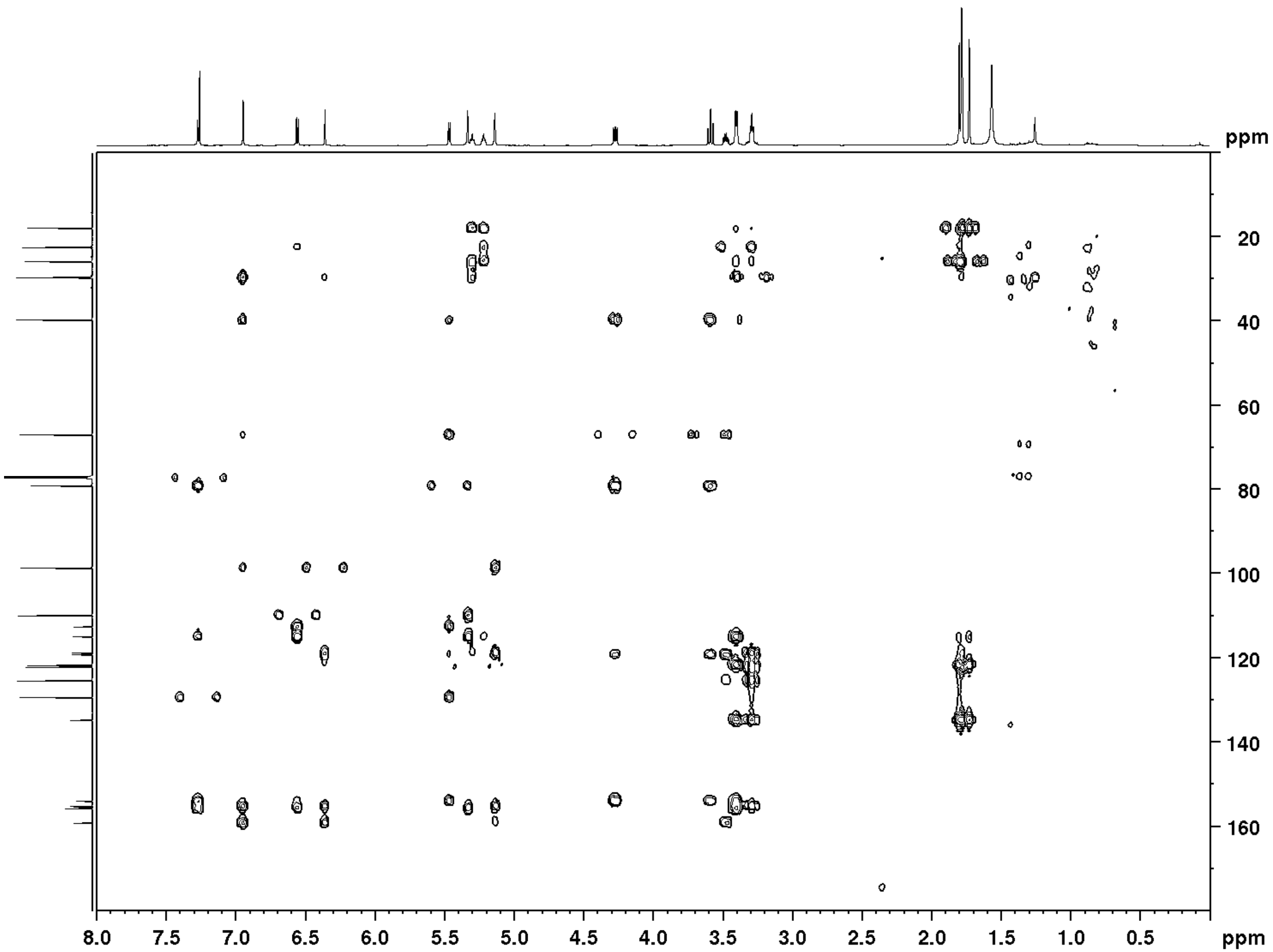

HMBC spectrum of erybraedin C (**5**) (600 MHz, $CDCl_3$)

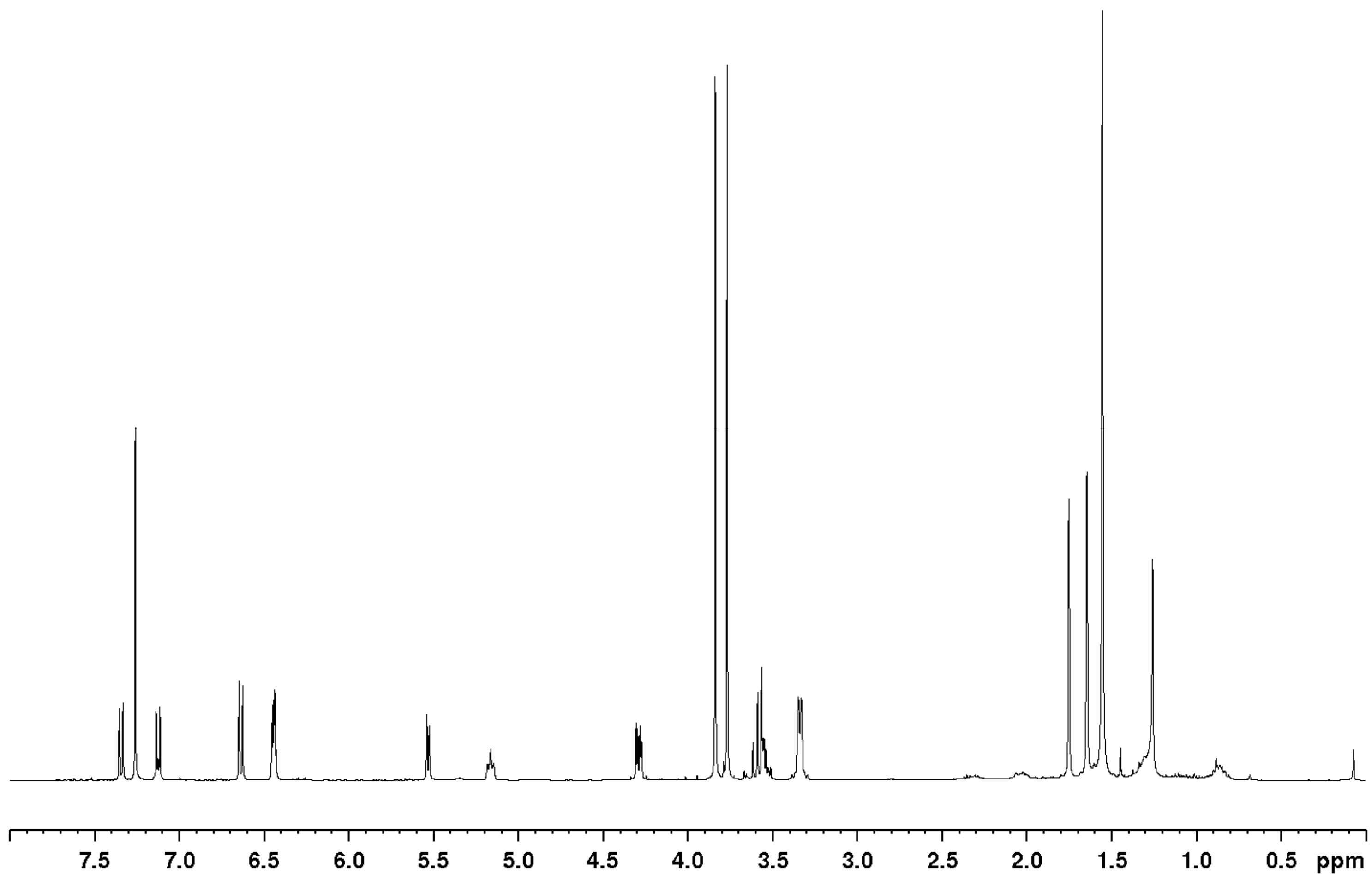

^{1}H NMR spectrum of bitucarpin A (**4**) (400 MHz, $CDCl_3$)

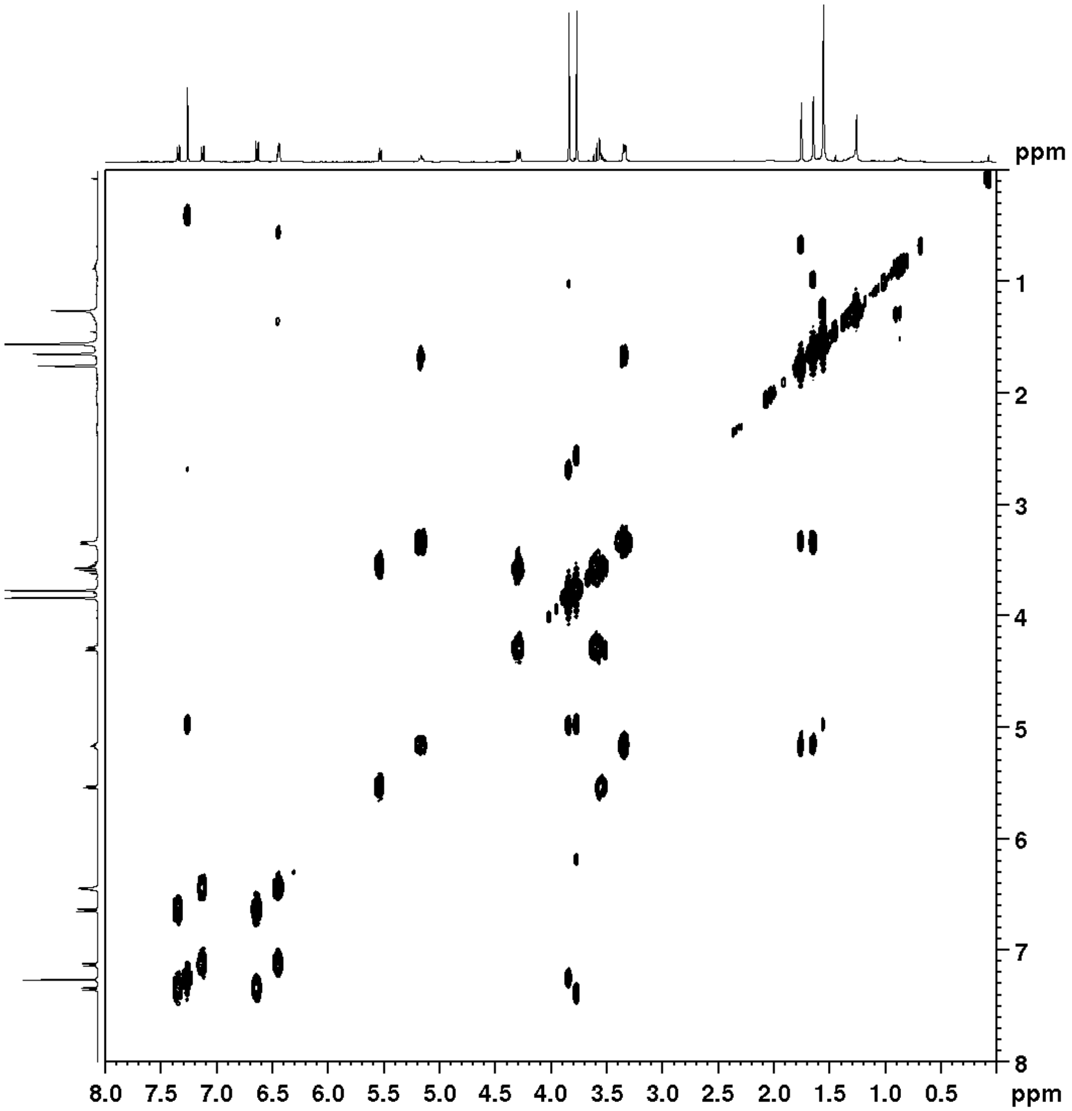

COSY spectrum of bitucarpin A (**4**) (400 MHz, $CDCl_3$)

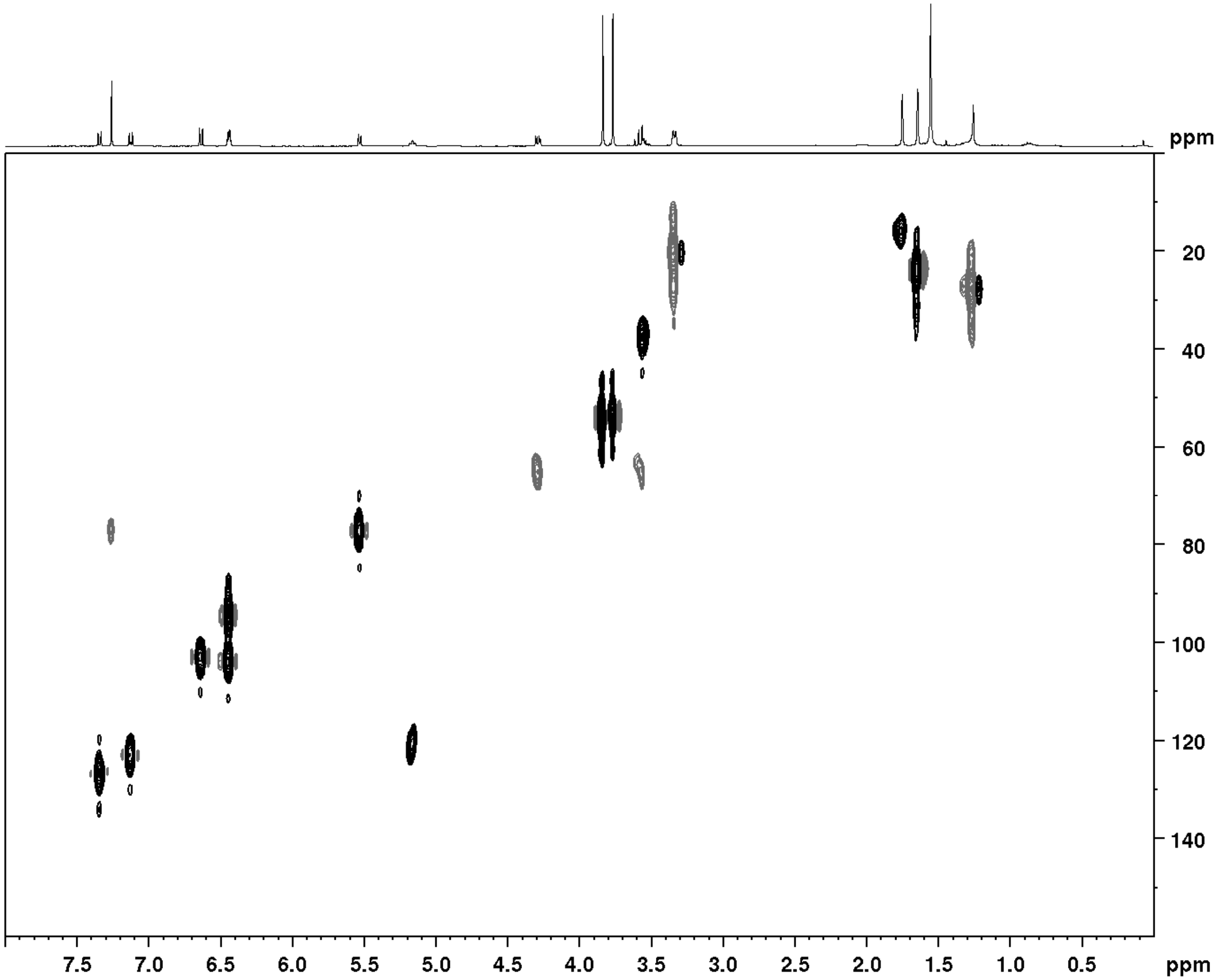

ed-HSQC spectrum of bitucarpin A (**4**) (400 MHz, $CDCl_3$)

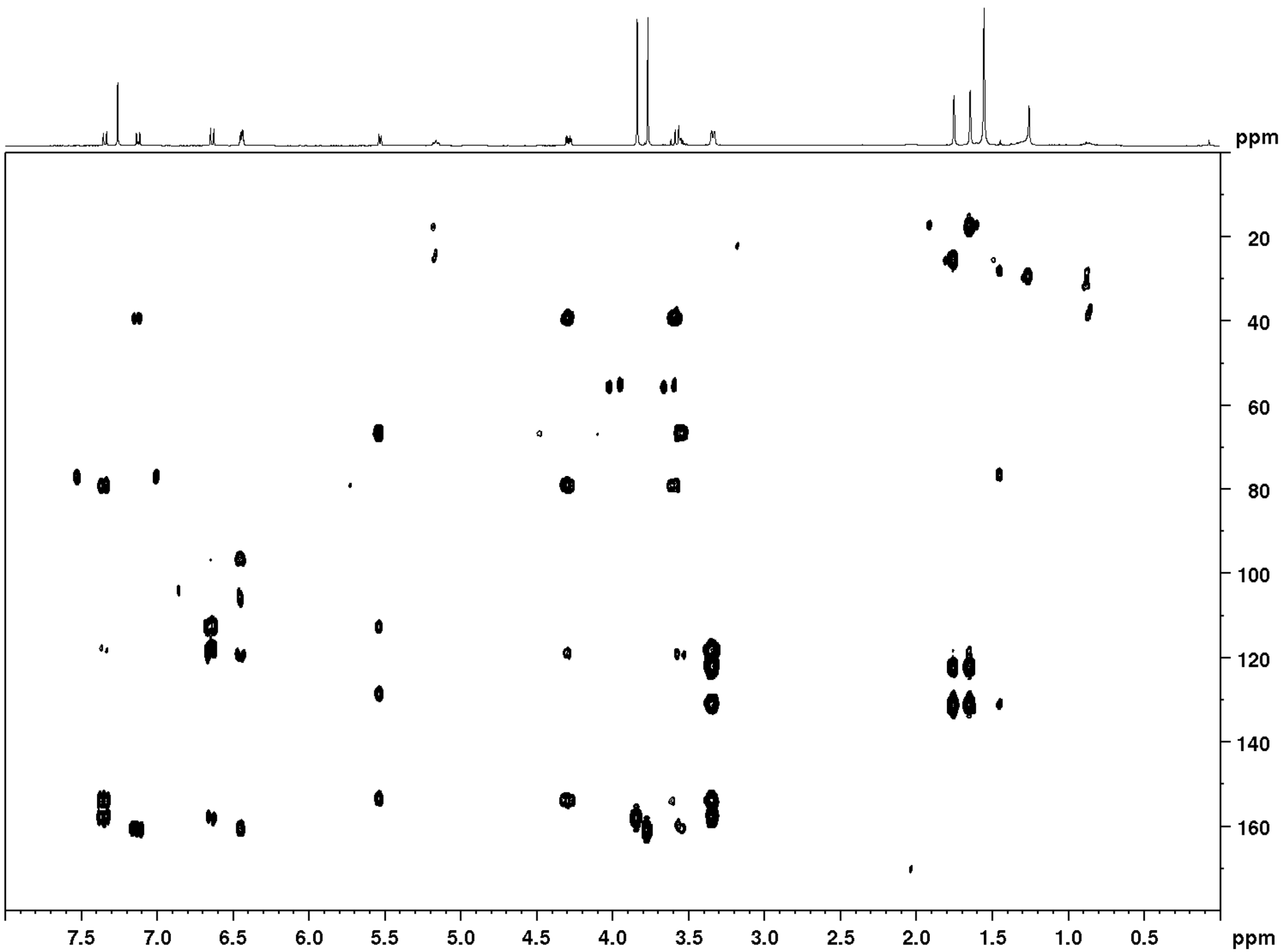

HMBC spectrum of bitucarpin A (**4**) (400 MHz, $CDCl_3$
